# Multi-stage transcriptome analysis identifies hub genes and regulatory mechanisms driving cervical cancer progression

**DOI:** 10.7717/peerj.21255

**Published:** 2026-05-20

**Authors:** Peng Li, Yawen Shao, Junling Wang, Ru Lin

**Affiliations:** 1 School of Public Health, Lanzhou University, Lanzhou, Gansu, China; 2 Cervical Cancer Prevention and Treatment Center, Gansu Provincial Maternity and Child-care Hospital, Lanzhou, Gansu, China

**Keywords:** Cervical cancer, RNA sequencing, Biomarkers, Bioinformatic analysis

## Abstract

**Background:**

Cervical cancer (CC) ranks as the fourth most prevalent cancer in women worldwide. While biomarkers exist, previous studies are often limited by public dataset heterogeneity, incomplete spectrum coverage, or lack of clinical validation. To address these limitations, this study aims to profile fresh tissues spanning the full continuum to systematically identify hub genes with dysregulated expression during malignant progression.

**Methods:**

This study collected cervical epithelial tissue from 10 patients each with low-grade squamous intraepithelial lesions (LSIL), high-grade squamous intraepithelial lesions (HSIL), and squamous cell carcinoma (SCC) for transcriptome sequencing. The differentially expressed genes (DEGs) analysis and Mfuzz clustering were employed to identify candidate genes showing monotonic expression trends. Candidate genes were prioritized using a protein–protein interaction (PPI) network with three centrality algorithms and validated in Gene Expression Omnibus to define hub genes. Subsequently, Gene Set Enrichment Analysis (GSEA), immune infiltration analysis, and regulatory network analyses were performed. Finally, hub gene expression was validated *via* reverse transcription quantitative polymerase chain reaction (RT-qPCR) in an independent clinical cohort.

**Results:**

A total of 1,654 DEGs (HSIL *vs.* LSIL) and 6,635 DEGs (SCC *vs.* HSIL) were identified. Mfuzz clustering and PPI analysis yielded nine candidates, with BUB1B, KIF14, and MELK finalized as hub genes *via* GEO validation. GSEA showed the three hub genes were significantly enriched in multiple shared pathways and exhibited stage-specific differences. Immune infiltration analysis showed no significant association between hub-gene expression and immune cell fractions. Regulatory network prediction implicated miRNAs (*e.g.*, hsa-miR-192-5p, hsa-miR-215-5p, hsa-miR-193b-3p) and TFs (*e.g.*, AF4, E2F1, FOXA1, KDM5B) as potential upstream regulators. The lncRNAs (*e.g.*, KCNQ1OT1, LINC01089, XIST3) were linked to these miRNAs. Finally, RT-qPCR in independent clinical samples confirmed hub-gene expression changes consistent with the bioinformatics findings.

**Conclusion:**

This study found the progressive upregulation of BUB1B, KIF14, and MELK across the LSIL-HSIL-SCC continuum, strengthening existing evidence of their association with cervical oncogenesis and highlighting their potential as candidate biomarkers for CC progression. However, the single-center design, limited sample size, and exploratory nature of the immune infiltration, regulatory network, and drug prediction analyses warrant cautious interpretation and underscore the need for further experimental validation.

## Introduction

Cervical cancer (CC) ranks as the fourth most common malignancy among women worldwide, following breast, lung, and colorectal cancers (Bray et al., 2024). According to estimates from the latest global cancer statistics, approximately 661,021 new cases and 348,189 deaths occurred in 2022, with age-standardized incidence and mortality rates of 14.1 and 7.1 per 100,000 women, respectively (Bray et al., 2024). The majority of cases were concentrated in Asia and Africa, imposing a substantial burden on local healthcare systems (Bray et al., 2024; Ferlay et al., 2021). Persistent high-risk human papillomavirus (hrHPV) infection is established as the principal driver of CC. By integrating into the host genome and disrupting cell-cycle regulation, hrHPV induces abnormal proliferation of cervical epithelial cells. Without timely intervention, these changes can progress to cervical precancerous lesions (CPLs) and eventually to invasive carcinoma. CPLs are stratified by severity into low-grade squamous intraepithelial lesions (LSIL/CIN 1) and high-grade squamous intraepithelial lesions (HSIL/CIN 2–3). Previous studies have demonstrated that LSIL is largely reversible, with approximately 60–89% of cases regressing spontaneously within 24 months, while only 11% progress to HSIL (Ciavattini et al., 2017; Loopik et al., 2021). In contrast, HSIL carries a markedly higher malignancy risk, with spontaneous regression rates ranging from 32% to 43%. Without intervention, the risk of progression to invasive squamous cell carcinoma (SCC) may increase by 5–22% (Li et al., 2021). Given that SCC development typically follows a stepwise progression from LSIL to HSIL to invasive carcinoma, early identification of distinct disease stages and implementation of targeted interventions are critical for reducing SCC incidence and improving patients’ quality of life and prognosis.

In recent years, high-throughput technologies have been increasingly applied in the field of CC for early diagnosis, treatment monitoring, and prognostic assessment. A previous review study involving multi-omics, such as microbiome, metabolomics, proteomics, and genomic analyses, has reported associations between CC risk and microbial dysbiosis, metabolic rewiring, protein-level alterations, and epigenetic changes, underscoring the biological complexity of cervical malignant transformation (Arip et al., 2022). Additionally, several integrative studies based on publicly available datasets such as the Gene Expression Omnibus (GEO) and The Cancer Genome Atlas (TCGA), as well as a limited number of independently generated datasets, have identified numerous differentially expressed genes and potential hub regulators implicated in cervical lesion progression. In particular, genes involved in cell-cycle regulation, mitotic control, and DNA replication—such as TOP2A, CDKN2A, RFC4, MCM5, MELK, and KIF14—have been reported to be associated with CC development and progression (Alvarado-Camacho et al., 2025; Asadzadeh, Ghorbani & Dastan, 2023; Niu et al., 2017; Xiao et al., 2021). However, despite these advances, several limitations remain in the current literature. First, many transcriptomic investigations rely primarily on retrospective public datasets and microarray platforms, which may involve heterogeneous sample sources, experimental conditions, and data processing pipelines. Second, the biological continuum spanning LSIL, HSIL, and SCC has not been systematically examined in a considerable proportion of studies, resulting in incomplete characterization of molecular alterations across progressive pathological stages. Third, existing analytical strategies rarely focus on identifying genes that exhibit persistent and monotonic dysregulation across the LSIL–HSIL–SCC trajectory, which may represent more robust indicators of disease evolution. Furthermore, validation of candidate biomarkers is often limited to public datasets or single-dimensional analyses, while independent verification using freshly collected clinical tissues remains limited. Therefore, it remains necessary to systematically investigate genes exhibiting persistently monotonic dysregulation during LSIL–HSIL–SCC progression within a unified framework of clinical sampling and pathological grading, and to perform dual validation using both public datasets and independent fresh tissues, thereby more accurately delineating core molecular events underlying the evolution from cervical lesions to invasive carcinoma.

In summary, the occurrence and progression of CC are a multi-staged complex process driven by persistent viral infection. This study aims to systematically identify and validate key hub genes associated with SCC progression through transcriptomic sequencing of cervical epithelial samples from patients with LSIL, HSIL, and SCC. We will also elucidate the expression dynamics, biological pathways, immune microenvironment influences, and drug sensitivities of these genes, exploring their potential as biomarkers. The findings are expected to provide theoretical insights and practical guidance for early classification, risk assessment, and targeted interventions in CC.

## Materials and Methods

### Data collection

The transcriptomic dataset used in this study was derived from 30 cervical lesion patients, including 10 cases each of LSIL, HSIL, and SCC. Cervical epithelial tissue samples were obtained *via* surgical resection or biopsy, immediately snap-frozen in liquid nitrogen following collection, and stored at −80 °C in an ultra-low temperature freezer to preserve RNA integrity.

Grouping criteria are based on histopathological diagnoses confirmed by specialized pathologists: (1) LSIL group: Lesions characterized by mild epithelial dysplasia, with maturation of the upper two-thirds of the squamous epithelium and mild nuclear atypia, while atypical cells and mitotic figures are confined mainly to the lower one-third of the epithelium. LSIL corresponds to cervical intraepithelial neoplasia grade 1 (CIN1). (2) HSIL group: Lesions showing moderate to severe dysplasia, with loss of epithelial maturation and atypical proliferating cells occupying more than one-third of the epithelial thickness, often accompanied by nuclear pleomorphism and pathological mitotic figures. HSIL corresponds to CIN2–CIN3. (3) SCC group: Samples diagnosed as invasive squamous cell carcinoma, characterized by epithelial cells breaching the basement membrane and infiltrating the underlying stroma, with variable degrees of keratinization or intercellular bridges depending on differentiation.

This study protocol was approved by the Ethics Review Committee of Gansu Provincial Maternity and Child-care Hospital (No. 2024GSFY04). All participants signed informed consent forms.

### Transcriptome sequencing and data preprocessing

The RNA was isolated and purified *via* the TRIzol (Invitrogen, Waltham, MA, USA) according to the scheme. Total RNA of each sample was quantified *via* NanoDrop ND-1000 (NanoDrop, Wilmington, DE, USA). Additionally, RNA integrity was verified *via* agarose electrophoresis and the Bioanalyzer 2100 (Agilent). Concentrations >50 ng/µL, OD260/280 >1.8, RNA integrity number (RIN) >7.0, and total RNA concentrations >1 µg were the filter criteria. Poly (A) RNA was purified from 1 µg total RNA utilizing Dynabeads Oligo (dT)25-61005 (Thermo Fisher Scientific, Waltham, MA, USA), and the mRNA was captured utilizing two rounds of purification. Then, the poly(A) RNA was fragmented into small pieces using Magnesium RNA Fragmentation Module (NEB, cat. e6150, USA) at 94 °C, and the time was set to 5–7 min. Reverse transcriptase then converted the cut RNA into cDNA using the reverse transcriptase. RNase H and Escherichia coli DNA polymerase I were then used to create cDNA duplexes. PCR, junction ligation, and poly-A tail addition were used to obtain the library from the double-stranded cDNA following magnetic bead purification (Invitrogen SuperScript™ II Reverse Transcriptase, cat. 1896649). Then, two-strand synthesis was carried out using *E. coli* DNA polymerase I (catalog number m0209; NEB), RNase H (NEB, catalog number m0297, USA), and dUTP Solution (cat.R0133; Thermo Fisher Scientific). The fragment size was then screened and purified using magnetic beads. The double-stranded DNA was digested with UDG enzyme (catalog number m0280; NEB). Then, through PCR (pre-denaturation at 95 °C for 3 min, denaturation at 98 °C for a total of 8 cycles, each lasting 15 s, annealing at 60 °C for 15 s, extension at 72 °C for 30 s, and final extension at 72 °C for 5 min), a library with fragment sizes of 300 bp ± 50 bp was formed. Finally, the Illumina Novaseq™ 6000 (LC Bio Technology CO., Ltd., Hangzhou, China) was utilized to perform paired-end sequencing on it according to the standard operation, with the sequencing mode being PE150. In the end, Fastp (https://github.com/OpenGene/fastp) was utilized to perform quality control on raw data. HISAT2 database (https://ccb.jhu.edu/software/hisat2) was utilized to compare sequencing data to the genome (Homo sapiens, GRCh38). Finally, StringTie software (v 2.2.3) (https://ccb.jhu.edu/software/stringtie/) was utilized to assemble and quantify the genes with FPKM.

This study included 10 biological replicates per group (LSIL, HSIL, and SCC), with each sample undergoing RNA sequencing only once. The statistical power of this experimental design is 0.86, calculated using the RNASeqPower online tool (https://rodrigo-arcoverde.shinyapps.io/rnaseq_power_calc/).

### Principal component analysis and correlation analysis

To ensure data quality and the reliability of inter-sample variability, principal component analysis (PCA) and Spearman correlation analysis were performed on the transcriptomic dataset. The former was employed to assess the overall distribution of samples, while the latter was used to evaluate the similarity of expression patterns within and between groups.

### Differentially expressed genes analysis

The differentially expressed genes (DEGs) analyses for cervical lesion samples were performed using the “DESeq2” package (version 1.42.0). Significantly DEGs were identified by applying thresholds of —log2FC— > 0.5 and adjusted *P*-value < 0.05, yielding two distinct gene sets: DEG1 (HSIL *vs.* LSIL) and DEG2 (SCC *vs.* HSIL). Volcano plots were generated to visualise the overall expression profiles for each comparison, whereas heat-maps illustrated clustering patterns of significantly dysregulated genes. Finally, a Venn diagram analysis was conducted to extract the intersecting genes between DEG1 and DEG2, which were defined as candidate genes associated with disease progression for downstream analyses.

### Cluster analysis

This study employed the MFuzz algorithm for clustering analysis of candidate genes. The optimal number of clusters was determined by applying the elbow method through the *k-means* function in the “cluster” package (version 2.1.6). Subsequently, the optimal fuzzy coefficient for the clustering model was automatically estimated using the *mestimate* function from the “MFuzz” package (version 2.62.0). Based on the above parameter settings, clustering analysis was conducted on the candidate genes, and expression patterns of each cluster were visualized using the *mfuzz.plot2* function.

### Gene Ontology and Kyoto Encyclopedia of Genes and Genomes functional enrichment analysis

Gene Ontology (GO) and Kyoto Encyclopedia of Genes and Genomes (KEGG) enrichment analyses were performed to clarify the biological functions and signaling pathways in which the key candidate genes participate during cervical lesion progression. GO enrichment was interrogated across three domains, biological processes (BPs), cellular components (CCs), and molecular functions (MFs), to characterise the functional attributes of the candidates. KEGG enrichment analysis was applied to identify signalling pathways and biological networks significantly over-represented among these genes. All analyses were implemented with the “clusterProfiler” package (version 4.8.3), and statistical significance was defined as *P* < 0.05.

### Protein-protein interaction network analysis and hub gene identification

The protein-protein interaction (PPI) network was constructed using the STRING database (http://string-db.org/), with an interaction confidence score threshold set to >0.400. Subsequently, the Degree, Closeness, and Radiality centrality algorithms were independently applied to select the top 10 of core node genes from the network. A Venn diagram was then used to identify the key intersection genes commonly identified by all three algorithms.

### Validation of hub gene expression

The GEO dataset GSE63514 was selected as an external validation set. This dataset comprises 104 cervical lesion tissue samples, including 36 LSIL (14 CIN1 and 22 CIN2 cases), 40 HSIL (CIN3 cases), and 28 SCC cases. To ensure comparability, GSE63514 was processed using platform-appropriate preprocessing, while downstream analyses were performed under the same gene-level annotation strategy and statistical criteria as those used in our transcriptomic dataset. Subsequently, we employed this validation dataset to externally validate the expression patterns of the hub genes, thereby assessing the reliability and consistency of expression differences for the candidate hub genes across different stages of cervical lesions.

### Gene Set Enrichment Analysis

Human gene sets were systematically retrieved from the MSigDB database (http://software.broadinstitute.org/gsea/msigdb/index.jsp) using the “msigdbr” package (version 7.5.1). The sequencing data were divided into low-expression and high-expression groups by comparing the expression levels of Hub genes between HSIL and LSIL, and between SCC and HSIL, with logFC values calculated to quantify gene differential expression. Then, the correlation between each biomarker and other genes was calculated by the *cor* function of the “WGCNA” package (version 1.72-5), and the genes were sorted by the correlation coefficients in descending order. Finally, Gene Set Enrichment Analysis (GSEA) was executed between HSIL and LSIL and between SCC and HSIL samples of the sequencing data *via* the “clusterProfiler” package (version 4.8.3), respectively, and the top five pathways were visualized based on *P* value *via* the *gseaNb* function of the “GseaVis” package (version 0.0.5).

### Immune infiltration analysis

To systematically characterize the dynamic evolution of the immune microenvironment during cervical lesion progression, we quantified the relative abundance of 22 immune cells in all samples using the CIBERSORT algorithm and designated those whose abundance changed in tandem with disease advancement as key immune cells. In order to further reveal the interaction networks between key immune cells and hub genes, as well as among key immune cells (screening threshold: —*cor*— > 0.3, *P* < 0.05), we used Spearman correlation analysis and visualized their co-variation trends *via* heatmaps.

### Construction of a molecular regulatory network

To investigate the regulatory mechanisms of hub genes, we built both a competing endogenous RNA (ceRNA) network and a miRNA-transcription factor (TF) network. First, potential MicroRNAs (miRNAs) targeting the hub genes were predicted with NetworkAnalyst (https://www.networkanalyst.ca) in conjunction with miRTarBase v9.0 (https://ngdc.cncb.ac.cn/databasecommons/database/id/167). Subsequently, long non-coding RNAs (lncRNAs) (Length > 300 nt and number of exons > 2) linked to miRNAs were screened using the Starbase database (https://rnasysu.com/encori/), and a gene-miRNA-lncRNA ceRNA regulatory network was constructed. Furthermore, we utilized the ChEA3 database (https://maayanlab.cloud/chea3/) to predict potential TFs for hub genes. Based on the predicted target miRNAs, a regulatory network was constructed encompassing hub genes, miRNAs, and TFs. Finally, the regulatory network was visualized using Cytoscape (version 3.9.1) to reveal the complex regulatory mechanisms of hub genes in CC.

### Prediction of drugs

The GeneCards (https://www.genecards.org) was utilized to forecast drugs, which integrates drug annotations from the MalaCards module (https://www.malacards.org) together with multiple curated resources (*e.g.*, DrugBank, DGIdb, ClinicalTrials.gov, PharmGKB, and ApexBio) and text-mining–based inference. Drug–gene associations for each hub gene were retrieved and ranked by drug approval status and the number/quality of supporting sources, and were used for hypothesis generation only without experimental validation.

### Reverse transcription quantitative polymerase chain reaction analysis

To validate the identified hub genes, we performed reverse transcription quantitative polymerase chain reaction (RT-qPCR) analysis on an additional 15 cervical epithelial tissue samples, including five cases each of LSIL, HSIL, and SCC. Total RNA was extracted from approximately 50 mg of frozen cervical tissue using TRIzol reagent (R401-01; Vazyme) following the manufacturer’s protocol. RNA concentration and purity were determined using a NanoDrop spectrophotometer (Thermo Fisher Scientific, USA), and only samples with appropriate A260/A280 ratios were used for further analysis. Complementary DNA (cDNA) was synthesized from 2 µg of total RNA in a 20 µL reaction volume using the HP All-in-one qRT Master Mix II kit (RT203-Ver.1; Kunming YunGen Biotech), according to the manufacturer’s instructions, under the following thermal conditions: 50 °C for 10 min, 85 °C for 5 s, and hold at 4 °C. Quantitative PCR was performed using a CFX Connect Real-Time PCR Detection System (BIO-RAD, USA) with 2 × Universal Blue SYBR Green qPCR Master Mix (G3326-05; Saiweier). Each 10 µL reaction contained 3 µL of cDNA, 5 µL of master mix, and 1 µL each of forward and reverse primers (10 µM). The amplification program consisted of an initial denaturation at 95 °C for 1 min, followed by 40 cycles of 95 °C for 20 s, 55 °C for 20 s, and 72 °C for 30 s. Melting-curve analysis was performed to confirm the specificity of each amplification. Each sample was analyzed in triplicate, and no-template controls were included to monitor contamination. The forward and reverse primer sequences for the hub genes and the reference gene (GAPDH) used in RT-qPCR are listed in Table S1. The composition of the RT-qPCR reaction is detailed in Table S2, and the thermal cycling parameters in Table S3. Relative mRNA expression levels were calculated using the 2^−^ΔΔCt method with GAPDH as the internal control. Both technical and biological replicates were included to ensure reproducibility. Data were analyzed using GraphPad Prism 10.1.2 (GraphPad Software). All procedures followed the MIQE guidelines to ensure accuracy, reproducibility, and transparency of the qPCR results.

### Statistical analysis

Bioinformatics analyses were executed using R (v 4.3.1; R Core Team, 2023). The *Wilcoxon* test or Student’s *t* test was utilized to compare the disparities between two groups. *P* < 0.05 was considered notably significant.

## Results

### Quality control

**Figure 1:**
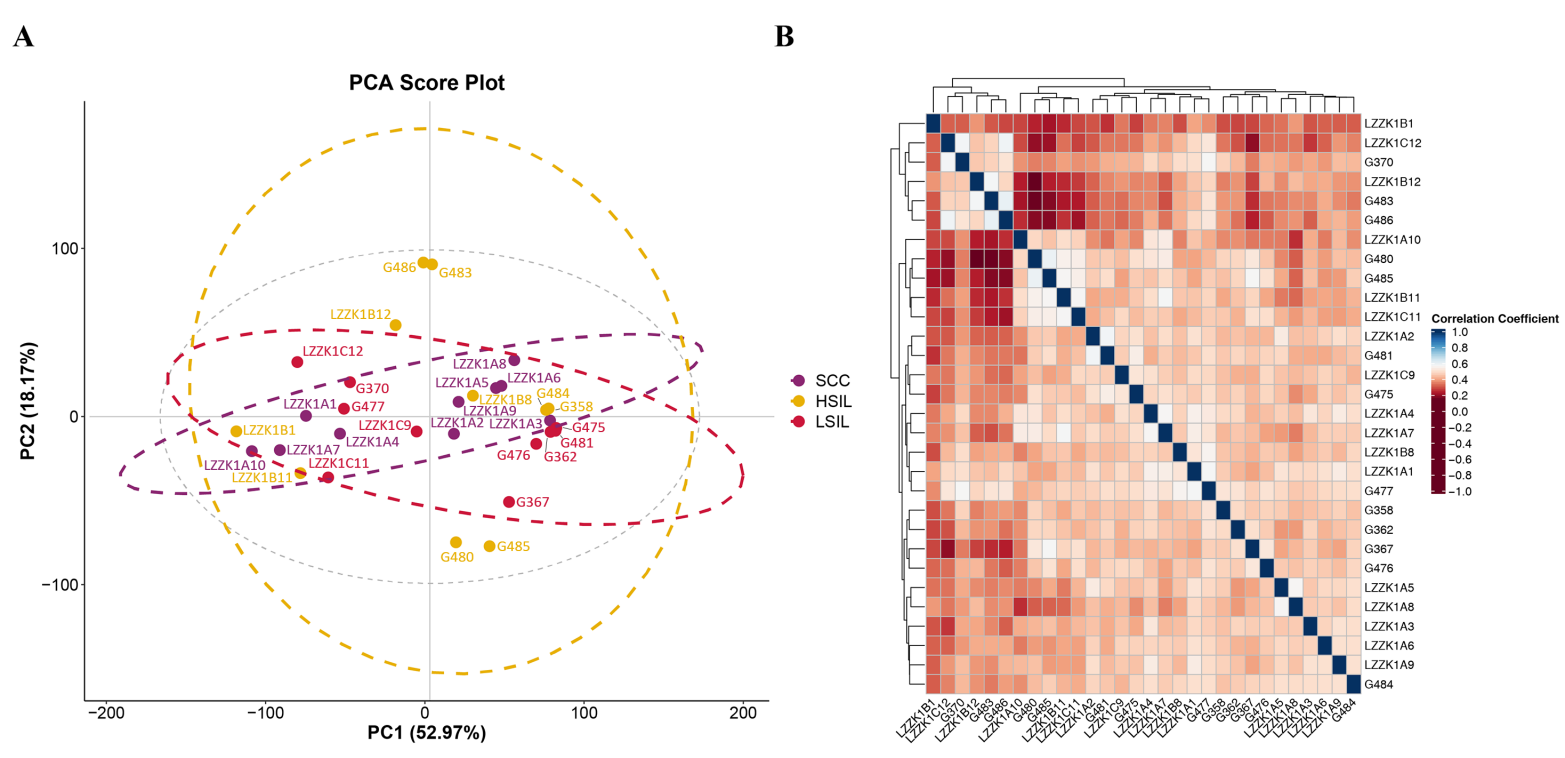
Data quality assessment. (A) PCA analysis of 30 cervical samples showing distinct clustering by disease stage. (B) Spearman correlation heatmap demonstrating stronger within-group correlations than between-group correlations (*P* < 0.05).

PCA revealed that PC1 and PC2 accounted for 52.97% and 18.17% of the variance, respectively, with a cumulative explained variance of 71.14% (Fig. 1A). Spearman correlation analysis demonstrated a significant clustering trend among samples within the same phenotypic group (*P* < 0.05), while weaker correlations were observed between different phenotypic groups (Fig. 1B). These results indicate high data quality and distinct phenotypic discriminability.

### The key candidate gene screening and identification

**Figure 2:**
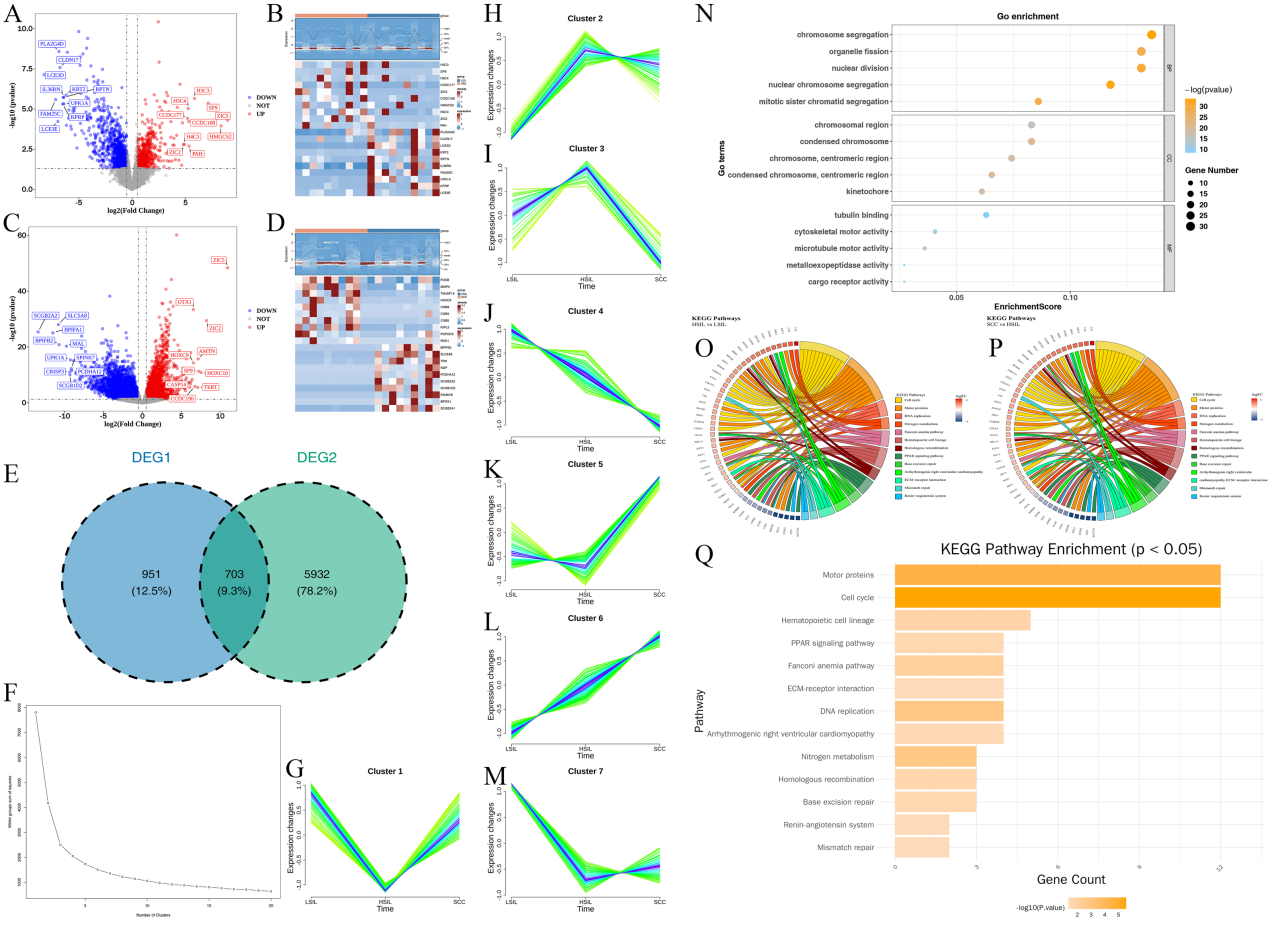
Key candidate gene screening and functional enrichment analysis. (A) Volcano plot of DEGs between HSIL and LSIL. Red and blue dots represent up- and down-regulated genes, respectively. (B) Hierarchical clustering heatmap of genes between HSIL and LSIL. The upper section presents a heatmap of expression density for the top 10 differentially expressed genes (upregulated/downregulated) in the HSIL group. The lower section displays an expression heatmap for 20 differentially expressed genes. (C) Volcano plot of DEGs between SCC and HSIL. (D) Hierarchical clustering heatmap of genes between SCC and HSIL. (E) Venn diagram shows the differentially expressed genes common to both the DEG1 and DEG2 sets. (F) K-means clustering analysis inflection point diagram. (G–M) Cluster expression change plot. (O) KEGG enrichment string diagram for HSIL *vs* LSIL. (P) KEGG enrichment string diagram for SCC *vs* HSIL. (Q) KEGG pathway enrichment analysis bar chart.

A total of 1,654 differentially expressed genes were identified in HSIL *vs* LSIL samples (DEG1), comprising 1,036 down-regulated and 618 up-regulated genes (Figs. 2A, 2B). 6,635 differentially expressed genes were identified in the SCC *vs* HSIL samples (DEG2), comprising 2,524 down-regulated and 4,111 up-regulated genes (Figs. 2C, 2D). After that, 703 candidate genes were obtained (Fig. 2E). Then, the MFuzz clustering results found that the optimal number of clusters was 7 (Fig. 2F). Among these, the expression levels of genes in Cluster 4 showed a continuous decline with increasing severity of cervical lesions (Fig. 2J), while those in Cluster 6 exhibited a persistent increasing trend (Fig. 2L). Therefore, this study selected genes from Clusters 4 and 6 for subsequent analysis, which demonstrated distinct expression patterns. Ultimately, 237 candidate genes were screened. GO analysis revealed that 237 candidate genes were enriched across 174 terms, including 137 BPs such as chromosome segregation, organelle fission, and nuclear division; 22 CCs including chromosomal region, condensed chromosome, and chromosome, centromeric region; 15 MFs such as tubulin binding, cytoskeletal motor activity, and microtubule motor activity (*P* < 0.05) (Fig. 2N, Table S4). In KEGG analysis, the candidate genes were enriched in 13 KEGG pathways, of which 12 key candidate genes were enriched in both the cell cycle and motor protein pathways (Figs. 2O–2Q).

### Identification of biomarkers

**Figure 3:**
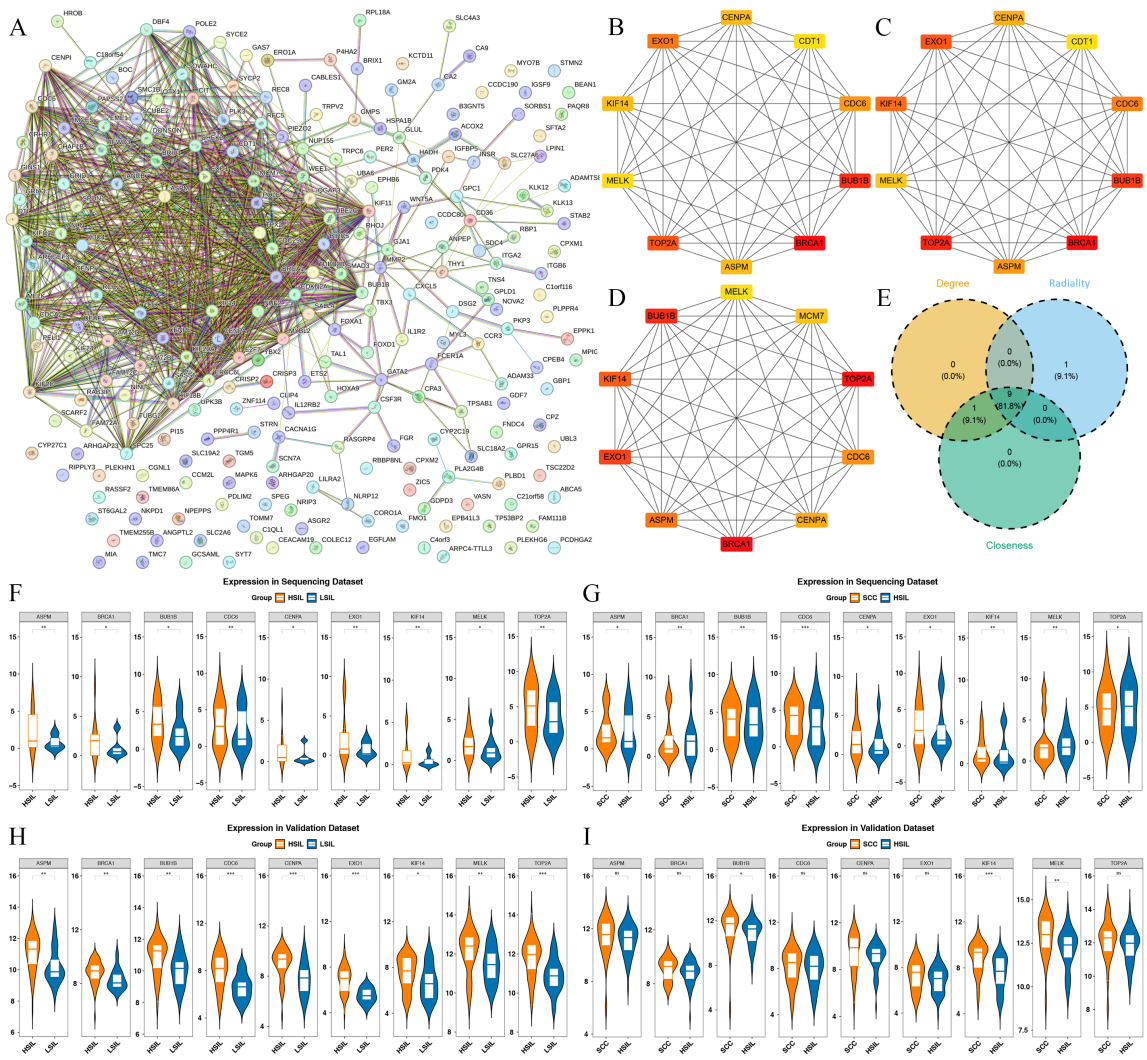
Identification of hub genes. (A) Candidate gene PPI network diagram. (B) Top 10 gene network diagrams ranked by degree algorithm. (C) Top 10 gene network diagrams ranked by the closeness algorithm. (D) Top 10 gene network diagrams ranked by the radicality algorithm. (E) Venn diagram shows the common genes present in the top 10 of the degree, closeness, and radiality algorithms. (F) Expression of hub genes in HSIL *vs.* LSIL groups from transcriptomic datasets. (G) Expression of hub genes in SCC *vs.* HSIL groups from transcriptomic datasets. (H) Expression of hub genes in HSIL *vs.* LSIL groups from the GSE63514 dataset. (I) Expression of hub genes in SCC *vs.* HSIL groups from the GSE63514 dataset. *P < 0.001, **P < 0.01, ***P < 0.05, ns indicates *P* > 0.05.

The key candidate genes were introduced to the STRING database to construct a PPI network, revealing interactions among 87 of these genes (Fig. 3A). Subsequently, the top 10 genes were independently identified using three centrality algorithms—Degree, Closeness, and Radiality (Figs. 3B–3D). Intersecting these results yielded nine candidate biomarkers (BRCA1, BUB1B, TOP2A, EXO1, CDC6, ASPM, CENPA, KIF14, MELK) (Fig. 3E). Further validation using the GSE63514 dataset revealed that BUB1B, KIF14, and MELK showed notable differences in both HSIL *vs.* LSIL and SCC *vs.* HSIL samples, showing an up-regulation trend (Figs. 3F–3I).

### Enrichment pathways of biomarkers

**Figure 4:**
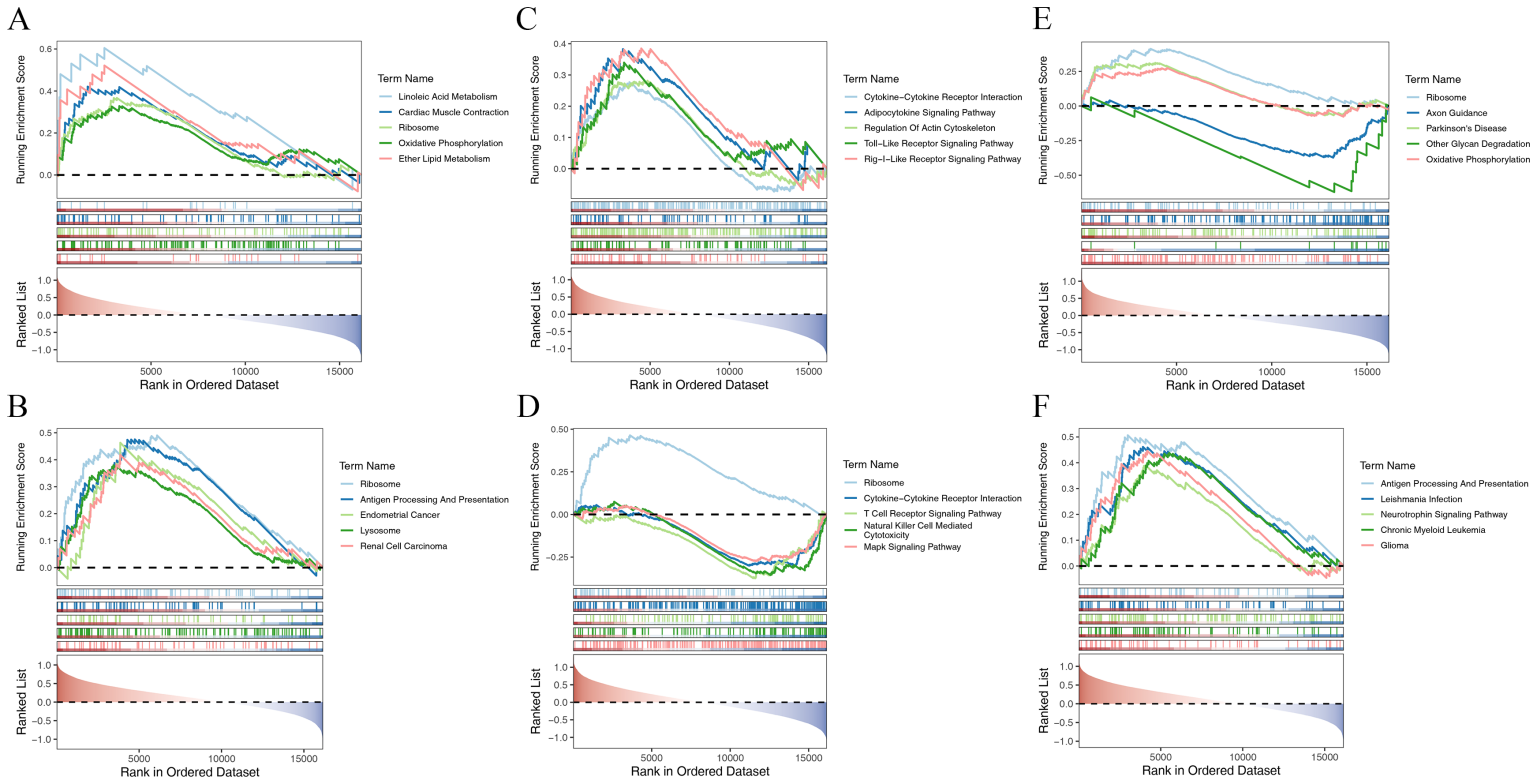
Gene Set Enrichment Analysis (GSEA) of hub genes. (A) GSEA results for BUB1B in HSIL *vs.* LSIL comparison, showing the top five significantly enriched pathways ranked by *P*-value. Each pathway is represented by an enrichment plot displaying the enrichment score (ES) and normalized enrichment score (NES). (B) GSEA results for BUB1B in SCC *vs.* HSIL comparison, displaying the top five enriched pathways. (C) GSEA results for KIF14 in HSIL *vs.* LSIL comparison, highlighting the most significantly enriched pathways including immune-related signaling cascades. (D) GSEA results for KIF14 in SCC *vs.* HSIL comparison, showing enrichment in multiple cellular signaling pathways. (E) GSEA results for MELK in HSIL *vs.* LSIL comparison, demonstrating enrichment in RNA processing and immune-related pathways. (F) GSEA results for MELK in SCC *vs.* HSIL comparison, showing continued enrichment in T-cell signaling and autoimmune-related pathways. The enrichment plots show the running enrichment score across the ranked gene list, with positive scores indicating upregulation and negative scores indicating downregulation in the respective comparisons.

BUB1B was significantly enriched in 18 and 16 pathways in the HSIL *vs.* LSIL and SCC *vs.* HSIL comparisons, respectively, with shared pathways comprising linoleic acid metabolism, ribosome, metabolism of xenobiotics by cytochrome P450, drug metabolism–cytochrome P450, adherens junction, and other glycan degradation (Figs. 4A–4B, Tables S5–S6). KIF14 exhibited enrichment in 14 and 32 pathways in the same two contrasts, and the overlapping pathways included cytokine–cytokine receptor interaction, adipocytokine signalling, regulation of actin cytoskeleton, Toll-like receptor, RIG-I-like receptor and T-cell receptor signalling, ribosome, chemokine signalling, and MAPK signalling (Figs. 4C–4D, Tables S7–S8). MELK was enriched in 18 and 20 pathways, respectively, with spliceosome, T-cell receptor signalling, and systemic lupus erythematosus emerging as common pathways (Figs. 4E–4F, Table S9–S10). Collectively, these significantly enriched pathways may underpin the initiation and progression of CC.

### Immune infiltration analysis in the CC

**Figure 5:**
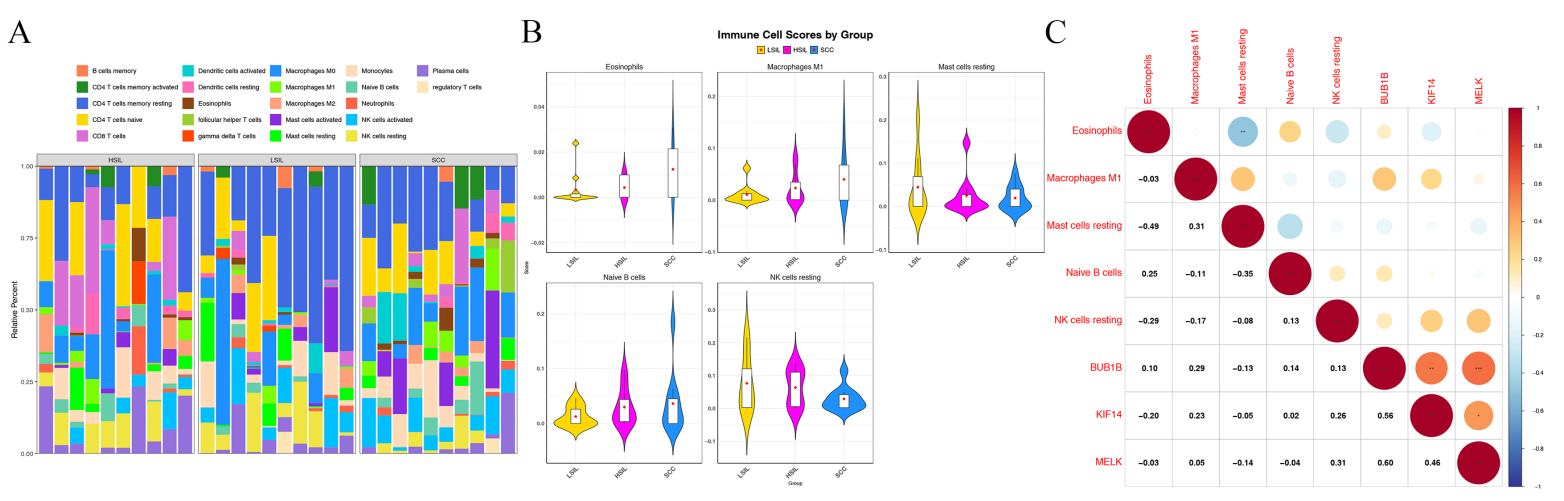
Immune cell infiltration analysis and correlation with hub genes. (A) Stacked bar chart showing the relative abundance of 22 immune cell types across LSIL, HSIL, and SCC groups. Each color represents a different immune cell type, and the height of each segment indicates the proportion of that cell type within each sample group. (B) Infiltration levels of five immune cell types in LSIL, HSIL, and SCC samples. (C) Correlation matrix diagram of five immune cell types. *P < 0.001, **P < 0.01, ***P < 0.05.

Immune cell infiltration analysis was performed on samples from the LSIL, HSIL, and SCC groups, obtaining the abundance of 22 immune cell types in each group (Fig. 5A). Eosinophils, M1 macrophages, and naive B cells showed progressively higher estimated proportions with increasing lesion severity, whereas resting mast cells and resting natural killer cells showed the opposite pattern (Fig. 5B). However, correlation analysis showed no significant associations between BUB1B, KIF14, and MELK expression and the infiltration levels of these immune cells (*P* > 0.05) (Fig. 5C).

### The upstream molecules and drugs related to biomarkers

**Figure 6:**
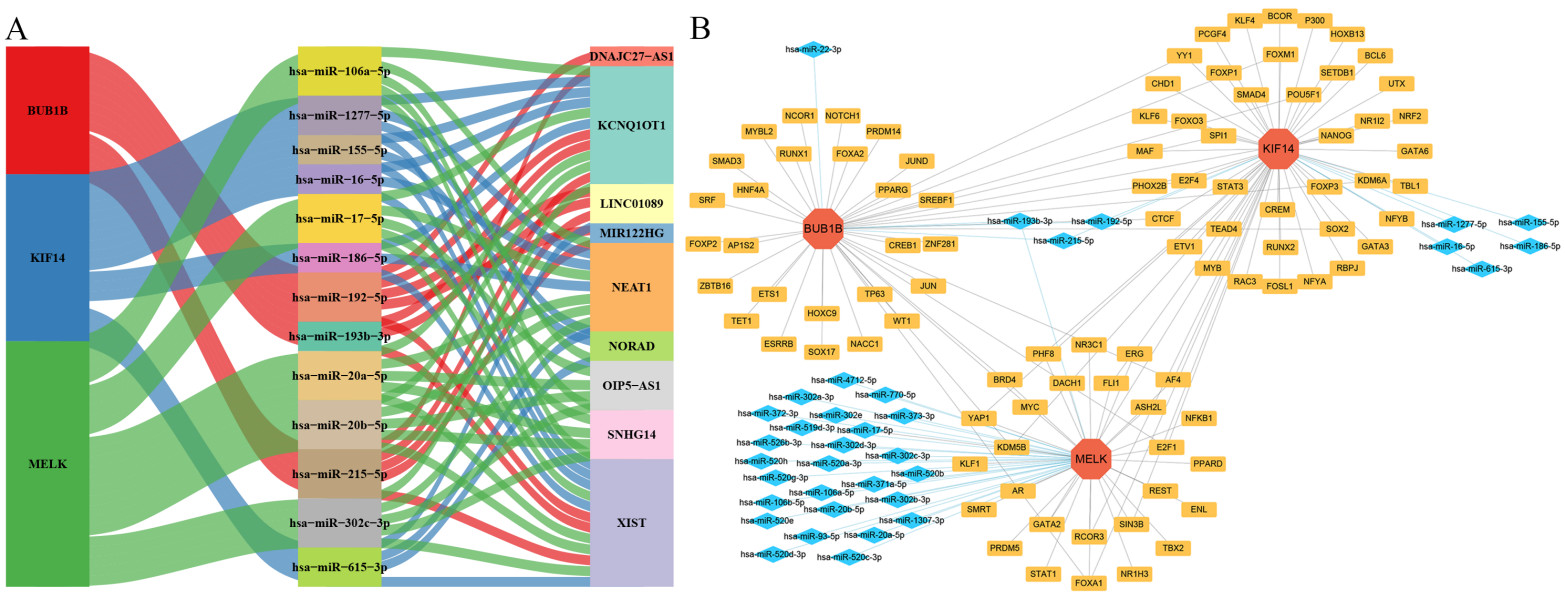
Molecular regulatory networks associated with hub genes. (A) ceRNA network showing interactions between three hub genes, 35 miRNAs, and 100 lncRNAs. (B) Integrated regulatory network of hub genes, miRNAs, and transcription factors (132 nodes, 157 edges). Key regulatory molecules include shared miRNAs and four common transcription factors.

In the ceRNA regulatory network analysis, we constructed a putative network comprising three hub genes, 35 miRNAs, and 100 lncRNAs based on public databases. Among these, two miRNAs, hsa-miR-192-5p and hsa-miR-215-5p, were predicted to interact with BUB1B and KIF14, whereas hsa-miR-193b-3p was predicted to interact with BUB1B and MELK. KCNQ1OT1, LINC01089, and XIST were predicted as candidate lncRNAs linked to these three miRNAs (Fig. 6A, Table S11). Further prediction identified 94 candidate TFs associated with hub genes, among which AF4, E2F1, FOXA1, and KDM5B were found to be associated with three hub genes. By integrating these predicted relationships, we established a comprehensive regulatory network consisting of 132 nodes and 157 edges (Fig. 6B, Table S12). Finally, drug prediction for the hub genes identified 8 drugs associated with BUB1B (Niflumic acid, Adenosine disphosphate, Lycorine chloride, Moniliformin, Doxorubicin, Paclitaxel, Vinblastine, and Nocodazole), and six drugs for MELK (Fostamatinib, SB 220025, Cenisertib, Dovitinib, Ilorasertib, and Leucine), while no drugs were predicted for KIF14.

**Figure 7:**
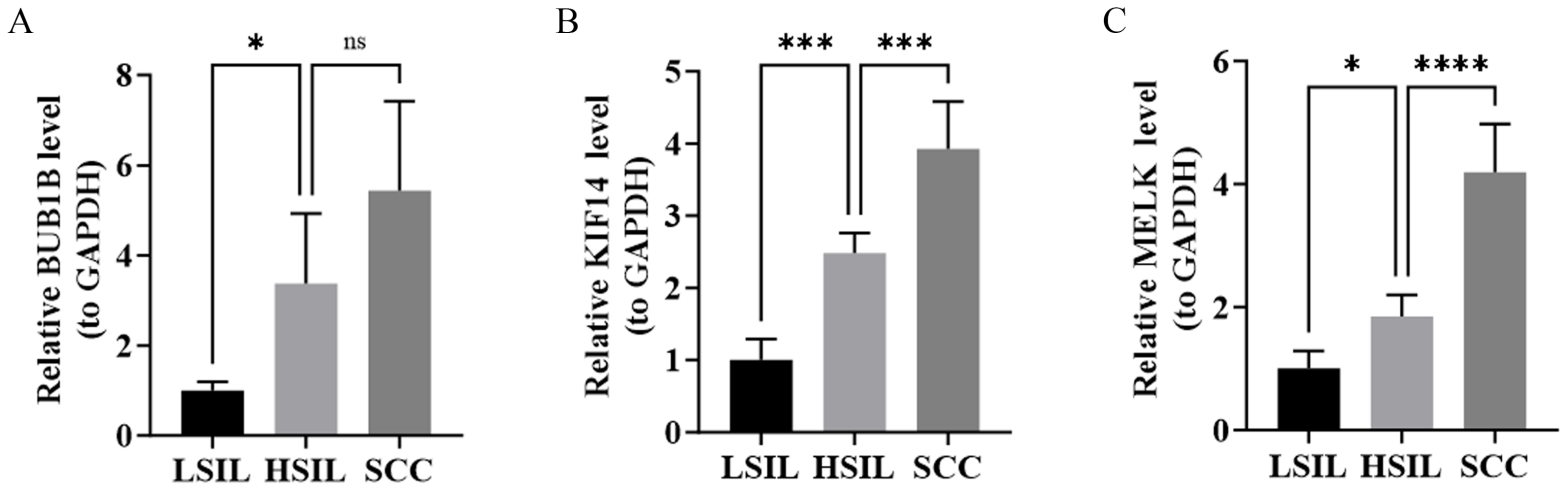
RT-qPCR validation of hub gene expression in clinical samples. (A) Expression levels of BUB1B in three sample groups. (B) Expression levels of KIF14 in three sample groups. (C) Expression levels of MELK in three sample groups. * *P* < 0.05, *** *P* < 0.001, **** *P* < 0.0001; ns > 0.05.

### RT-qPCR experiments of biomarkers

RT-qPCR analysis of clinical samples demonstrated that BUB1B expression was significantly higher in HSIL than in LSIL, whereas the increase from HSIL to SCC did not reach statistical significance, despite an apparent upward trend (Fig. 7A). The expression levels of KIF14 and MELK were significantly up-regulated in both comparisons (HSIL *vs.* LSIL and SCC *vs.* HSIL) (Figs. 7B–7C). Overall, these experimental results are directionally consistent with our transcriptomic profiling, reinforcing the association between the upregulation of these candidate biomarkers and cervical lesion severity.

## Discussions

Biomarker identification holds significant importance for the early diagnosis, treatment decision-making, and prognostic evaluation of CC and CPL, providing critical support for the advancement of precision medicine (Arip et al., 2022). This study systematically constructed a disease-progression continuum spanning the LSIL-HSIL-SCC, identifying three hub genes (BUB1B, KIF14, and MELK) that were consistently up-regulated across different stages. These genes were significantly enriched in several critical pathways, such as linoleic acid metabolism, ribosome, and systemic lupus erythematosus. Immune infiltration analysis showed trends in the abundance of five immune cells associated with disease progression, but did not reach statistical significance. Regulatory network analysis identified several regulatory relationships involving specific miRNAs, lncRNAs, and TFs. Furthermore, Drug prediction identified multiple potential compounds associated with BUB1B and MELK. Collectively, this work strengthens molecular stratification along the LSIL–HSIL–SCC axis and provides prioritized, biologically grounded candidates to support future precision-diagnostic and mechanistic studies in cervical lesions.

BUB1B, located at chromosome 15q15.1, is a pivotal mitotic regulator that activates, maintains, and silences the spindle assembly checkpoint (SAC), regulates chromosome-spindle attachment, and controls mitotic timing (Bloom & North, 2021). Previous studies have demonstrated heterogeneous effects of BUB1B across different cancers, where its aberrant expression may lead to chromosomal instability and increased cancer incidence (Dominguez-Brauer et al., 2015). For instance, down-regulation of BUB1B has been associated with the development of colorectal cancer (Park et al., 2007), while its up-regulation has been linked to higher risks of lung adenocarcinoma (Ding et al., 2024), breast cancer (Koyuncu et al., 2021), ovarian cancer (Feng et al., 2019), and endometrial cancer (Zhang, Li & Lu, 2024). In this work, BUB1B was consistently up-regulated from LSIL-HSIL-SCC, suggesting its potential key role in CC progression, although the exact mechanisms require further investigation. Furthermore, although RT-qPCR did not show a statistically significant difference in BUB1B expression between HSIL and SCC, which may reflect the limited validation sample size and the inherent inter-tumor heterogeneity characteristic of invasive cervical squamous cell carcinoma, the observed upward trend was consistent with the transcriptomic data, suggesting that BUB1B may be associated with lesion severity.

KIF14, mapped to chromosome 1q32.1, belongs to the kinesin superfamily proteins that broadly participate in cell division, proliferation, and migration (Hu et al., 2024). Up-regulation of KIF14 has been demonstrated to exhibit potential oncogenic properties across various cancers, such as retinoblastoma, lung cancer, breast cancer, gastric cancer, ovarian cancer, and cholangiocarcinoma (Jiang et al., 2023a; Liu et al., 2022; Yang et al., 2019; Yu & Feng, 2010). Its oncogenic potential is primarily attributed to disruptions in cytokinesis and chromosome segregation, and such dysregulation of cell cycle control is considered a critical factor in cancer development (Li & Stanger, 2020). In CC, the carcinogenic mechanisms of KIF14 have been elucidated by multiple studies, including inhibiting p27^Kip1^ degradation to induce cell cycle arrest (Zhang et al., 2022), and activating the PI3K-AKT-mTOR pathway through interaction with Reticulocalbin 1 (RCN1) to promote carcinogenic signaling (Li et al., 2025), thereby facilitating malignant transformation in CC cells. Moreover, KIF14 upregulation has also been associated with increased chemoresistance in CC, ultimately resulting in reduced patient survival time (Wang et al., 2016). Our findings further support the potential role of KIF14 in the diagnosis and treatment of CC, providing evidence for the development of novel therapeutic strategies.

MELK is located at chromosome 9p13.2 and belongs to the AMP-activated protein kinase family of serine-threonine kinases. Its physiological functions primarily involve cell proliferation, apoptosis, cell cycle regulation, spliceosome assembly, stem cell self-renewal, and metabolism (Sun et al., 2021; Thangaraj et al., 2020). MELK overexpression has been demonstrated to have significant cancer-promoting effects in various cancer types, such as breast, lung, endometrial, prostate, ovarian cancer, uterine leiomyosarcoma, leukemia, and myeloma, as detailed in the comprehensive review by Thangaraj et al. (2020). In CC, MELK has similarly been found to be significantly upregulated and to profoundly impact the proliferation and colony formation ability of various CC cell lines (Wang et al., 2018). Knockdown of MELK significantly inhibits cell proliferation and increases apoptosis in CC cells (Wang et al., 2018). Moreover, MELK overexpression reprograms the immune microenvironment by activating NF-κB signaling and promoting IL-6 secretion, shifting the Th1/Th2 immune balance toward Th2 dominance. This immunomodulatory effect weakens anti-tumor immune responses, enabling CC cells to evade immune surveillance. In addition, the reduced antitumor activity of CD8+ T cells is also associated with MELK overexpression, which could diminish the immune system’s clearance ability against CC cells and accelerate tumor growth (Wang et al., 2024).

GSEA enrichment analysis revealed that BUB1B, KIF14, and MELK were significantly enriched in several shared pathways and exhibited stage-specific differences during the progression from LSIL-HSIL-SCC. BUB1B-related pathways involved cellular metabolic reprogramming and dynamic regulation of cell adhesion. In early lesions, BUB1B up-regulates cytochrome P450-related metabolic pathways (linoleic acid metabolism, metabolism of xenobiotics by cytochrome P450, and drug metabolism—cytochrome P450), promoting metabolic adaptation during malignant transformation (Abdelmonem et al., 2024; Manikandan & Nagini, 2018). Conversely, during SCC, the down-regulation of these pathways directly diminishes the antitumor efficacy of SCC-related chemotherapeutic drugs, such as hindering the activation of the cyclophosphamide (El-Serafi et al., 2015), and decreasing the metabolic rate of taxane drugs, leading to drug accumulation and increased toxic side effects (Cresteil et al., 2002). Concurrently, down-regulation of the adherens junction and other glycan degradation pathways in the HSIL *vs.* LSIL group reduces cell adhesion and weakens the cervical epithelial barrier, which helps lesion cells develop greater local migration and abnormal proliferation capabilities (Cai et al., 2018; Li et al., 2016). Notably, during the HSIL-SCC, these pathways are reactivated to facilitate tumor cell aggregation, matrix invasion, and anti-apoptotic capabilities. In addition, the sustained up-regulation of the Ribosome pathway answers the urgent demand for protein synthesis driven by rapid proliferation throughout the entire lesion process (Kang et al., 2021). KIF14 was predominantly enriched in immune response and signal transduction pathways, with its enrichment changes revealing the ongoing remodeling of the tumor immune microenvironment. During the transition from LSIL to HSIL, the up-regulation of multiple signaling pathways (cytokine-cytokine receptor interaction, regulation of actin cytoskeleton, and Toll-like receptor signaling pathway) related to immune responses and environmental sensing reflects a host immune response mobilised to eliminate HPV and abnormal cells. In contrast, broad down-regulation of these pathways upon progression to SCC signifies a shift from immune activation to immune suppression, thereby potentiating tumor immune escape, autonomous proliferation, and invasion capacity. For instance, down-regulation of the Toll-like receptor signaling pathway directly impairs the recognition of tumor cells, while suppression of the RIG-I-like receptor signaling pathway inhibits the transcriptional induction of type I/III interferons and other cytokines, thereby weakening anti-tumor immunity (Cen, Liu & Cheng, 2018; Jiang et al., 2023b). Consistent with BUB1B, the ribosome pathway also showed an up-regulation pattern in the KIF14 high-expression group across both stages. The enrichment pathways of MELK suggest its dual roles in RNA processing and immunoregulation: The spliceosome pathway was down-regulated during the LSIL-HSIL but up-regulated during HSIL-SCC. This reversal suggests a functional shift from maintaining cellular homeostasis to promoting malignant evolution. Its up-regulation may drive extensive alternative splicing, generating protein variants that facilitate tumor growth and provide the foundation for tumor cell proliferation, apoptosis, invasion, metastasis, angiogenesis, and metabolism (Zhang et al., 2021). Notably, similar to KIF14, MELK was also significantly enriched in the T cell receptor (TCR) signaling pathway, yet their relationships with TCR signaling were opposite. This may imply distinct and stage-specific contributions in immune-microenvironment modulation. Additionally, systemic lupus erythematosus was consistently upregulated across both stages, which may influence tumor initiation and progression by affecting the tumor microenvironment through processes such as apoptosis, immunosuppression, or immune activation (Wang et al., 2022a).

Immune infiltration analysis revealed that with the malignant progression of cervical lesions, the abundances of eosinophils, M1 macrophages, and naive B cells showed a progressive increase, while resting mast cells and resting natural killer cells exhibited a sustained decline. Similar patterns of immune cell composition changes have been reported in previous studies (Kumar, Bauer & Stewart, 2023; Xia et al., 2025; Zhang et al., 2025; Zhou et al., 2025), suggesting that the development of CC involves not only the abnormal proliferation of tumor cells themselves but also continuous remodeling and functional imbalance of the immune microenvironment. However, no significant correlations were observed between the expression levels of the three hub genes and the infiltration levels of these five immune cell types. Therefore, our data do not support a direct association between the expression of these genes and immune infiltration at the bulk-tissue level. These findings suggest that the hub genes are more likely reflecting proliferative activity, consistent with their established roles in cell-cycle regulation.

Regulatory network analysis provided a hypothesis-generating framework for potential upstream regulation of BUB1B, KIF14, and MELK. Specifically, both hsa-miR-192-5p and hsa-miR-215-5p were predicted to interact with BUB1B and KIF14, while hsa-miR-193b-3p was predicted to interact with BUB1B and MELK. Although direct experimental evidence supporting these miRNA–hub gene regulatory relationships in cervical lesions is currently lacking, existing studies indicate that these miRNAs are associated with cell-cycle–related or tumor-suppressive phenotypes in CC or other malignancies: hsa-miR-192-5p suppresses CC cell proliferation and invasion by targeting transient receptor potential melastatin-subfamily member 7 (TRPM7) (Dong et al., 2021); hsa-miR-215-5p inhibits cell invasion in breast cancer through targeting SRY-Box 9 (SOX9) (Gao, Zhu & Zhu, 2019); and miR-193b blocks cell cycle progression in CC by targeting Cyclin D1 (CCND1) (Huang et al., 2021). Given the known roles of BUB1B, KIF14, and MELK in cell cycle regulation, we speculate that hsa-miR-192-5p, hsa-miR-215-5p, and hsa-miR-193b-3p might represent candidate components of a tumor-suppressive network in CC. However, lncRNAs including KCNQ1OT1, LINC01089, and XIST were identified as candidate ceRNAs linked to these three miRNAs, and may be involved in ceRNA-like regulatory relationships that could influence miRNA-mediated repression. Notably, several of these lncRNA–miRNA interactions have been reported in previous studies, including KCNQ1OT1–miR-192-5p, LINC01089–miR-193b-3p, XIST–miR-215-5p, and XIST–miR-192-5p (Cheng & Wang, 2020; Liang et al., 2025; Sun et al., 2020). These observations provide indirect support for the plausibility of the predicted regulatory framework, although their functional relevance in CC remains to be experimentally validated. TFs exert critical control over gene regulation, orchestrating the conversion of genetic information into functional molecules (Caputo et al., 2023). We predicted 94 candidate TFs associated with hub gene regulation, with AF4, E2F1, FOXA1, and KDM5B prioritized because they were linked to all three hub genes in the TF prediction analysis. Among these, AF4 serves as the molecular foundation of super-elongation complexes (SECs) and has been implicated in transcriptional regulation (Scholz et al., 2015). E2F1 is a tumor suppressor factor regulating cell cycle transition (G1/S phase), cell proliferation, and apoptosis (Navarro et al., 2013). FOXA1 promotes cancer cell proliferation, migration, invasion, and metastasis (Yuan et al., 2024), while KDM5B is implicated in cellular proliferation, migration, and apoptosis (Zhou et al., 2017). The roles of the latter three TFs in CC progression have been previously documented (Sun et al., 2021; Wang et al., 2022b; Zhu et al., 2024).

Drug prediction analysis identified several compounds associated with the hub genes, including microtubule inhibitors (Paclitaxel, Vinblastine, Nocodazole) for BUB1B, aurora kinase inhibitors (Cenisertib, Ilorasertib) and multi-kinase inhibitors (Dovitinib, Fostamatinib) for MELK. BUB1B’s involvement in spindle assembly checkpoint regulation and chromosome segregation provides a rationale for microtubule-targeting agents (Bloom & North, 2021), while MELK’s role in cell cycle progression and proliferation offers biological plausibility for the potential efficacy of aurora and multi-kinase inhibitors (Thangaraj et al., 2020). Although no drugs were predicted for KIF14, its participation in cytokinesis and PI3K-AKT-mTOR signaling may guide future exploration of pathway-targeted compounds (Li et al., 2025). Overall, these predictions offer mechanistically informed hypotheses for future experimental validation in CC.

This study has several limitations. First, the clinical samples were obtained from a single center, which may limit the representativeness and generalizability of our findings. therefore, validation in independent, multi-center populations is warranted. Second, the sample sizes used in this study are relatively small, including both the primary transcriptomic sequencing dataset (*n* = 10 per group) and the subsequent RT-qPCR validation dataset (*n* = 5 per group). While these numbers allowed us to observe consistent expression trends, the limited sample size may introduce sampling bias and restrict the statistical robustness of our validation. Therefore, larger independent validation studies are warranted. Third, although we systematically analyzed the potential functions and regulatory mechanisms of the hub genes using multiple bioinformatics approaches, these findings remain primarily predictive and correlative. In particular, immune infiltration estimates and therapeutic/drug predictions were derived from computational inference and database mining and should be considered hypothesis-generating until validated by orthogonal assays and functional experiments. Thus, further experimental validation through *in vitro* and *in vivo* studies is essential to elucidate the biological relevance of these key genes in CC development and progression. Finally, HPV infection status and genotype were not available in this study; thus, we could not perform HPV-stratified or HPV-adjusted analyses. Importantly, our analyses were anchored to histopathologically defined lesion grades, and the progressive up-regulation of BUB1B, KIF14, and MELK was observed consistently across LSIL, HSIL, and SCC at the tissue level, with external transcriptomic support and RT-qPCR confirmation. Given that persistent high-risk HPV infection is the necessary etiologic basis for high-grade lesions and invasive carcinoma, the molecular changes captured here may reflect downstream, clinically relevant consequences of HPV-driven carcinogenesis. While specific HPV genotypes could potentially modulate the magnitude of these signals, the stage-associated expression patterns we report remain linked to the pathological phenotype and therefore retain potential value for risk stratification and clinical monitoring.

## Conclusion

In summary, BUB1B, KIF14, and MELK were associated with cervical lesion severity and exhibited a sustained upregulation trend from LSIL to HSIL and SCC. These findings consolidate the evidence supporting these genes as progression-associated candidates in CC, nominating them as potential biomarkers whose expression patterns may inform future strategies for early risk stratification and clinical monitoring.

##  Supplemental Information

10.7717/peerj.21255/supp-1 Supplemental Information 1 Raw data

10.7717/peerj.21255/supp-2 Supplemental Information 2 Supplementary Tables and Figures

10.7717/peerj.21255/supp-3 Supplemental Information 3 MIQE checklist

## Additional Information and Declarations

### Data Availability

The following information was supplied regarding data availability: The raw data is available in the Supplemental File. The raw sequence data is available at the National Genomics Data Center Genome Sequence Archive: HRA014345.
