# Supplementary material for: Multi-stage transcriptome analysis identifies hub genes and regulatory mechanisms driving cervical cancer progression
Source: PeerJ. 2026 May 20;14:e21255. doi: 10.7717/peerj.21255 (PMC13198200; doi:10.7717/peerj.21255)
Supplement: Supplemental Information 2 [file peerj-14-21255-s002.docx]

**Supplementary Table 1.** Forward and reverse primers for RT-qPCR

| **Gene** | **Accession no.** | **Primer** | **Sequence (5′→3′)** | **Amplicon length (bp)** |
| --- | --- | --- | --- | --- |
| BUB1B | NM_001211.6 | Forward | AGGTCTTCTGGGATGGGTCC | 240 |
|  |  | Reverse | GAAAGAGCAAAGCCCCAGGA | 240 |
| KIF14 | NM_001305792.1 | Forward | TGTCCAAAGAAGAGCTTAGGG | 299 |
|  |  | Reverse | AGGGGCATGTCTGCTGTTTT | 299 |
| MELK | XM_054364313.1 | Forward | GCTCAGCCGTGCCCTC | 227 |
|  |  | Reverse | TGATCCGGGGCAAATCACTC | 227 |
| GAPDH (reference) | NM_001256799.3 | Forward | ATGGGCAGCCGTTAGGAAAG | 135 |
|  |  | Reverse | AGGAAAAGCATCACCCGGAG | 135 |

**Supplementary Table 2. RT-qPCR reaction system**

| **component** | **Volume** |
| --- | --- |
| cDNA | 3ul |
| 2xUniversal Blue SYBR Green gPCR Master Mix | 5ul |
| PCR forward primer (10 µM) | 1ul |

**Supplementary Table 3. RT-qPCR thermal cycling parameters**

| **Step** | **Temperature** | **Time** |
| --- | --- | --- |
| Initial denaturation | 95 °C | 1 min |
| Denaturation (40 cycles) | 95 °C | 20 s |
| Annealing | 55 °C | 20 s |
| Extension | 72 °C | 30 s |


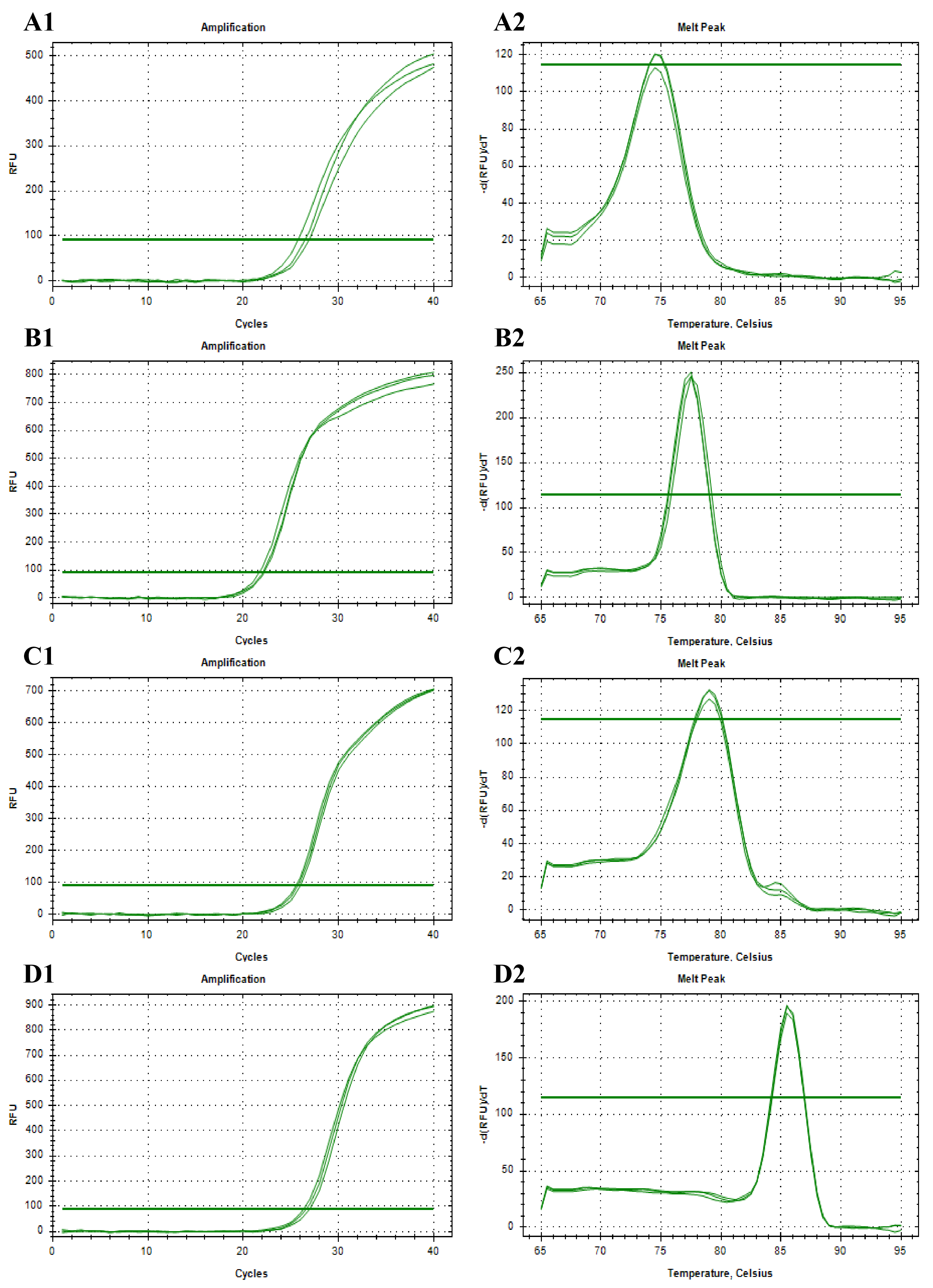


**Supplementary** Figure 1. Amplification and melting curves for BUB1B, KIF14, MELK, and GAPDH.

1. BUB1B; (B) KIF14; (C) MELK; (D) GAPDH. For each gene, 1 shows the amplification curve, and 2 shows the corresponding melting curve. All amplification curves exhibit typical sigmoidal trajectories with clear exponential phases. All melting curves display single, sharp peaks, confirming amplification specificity.

**Supplementary Table 4.** GO analysis of 237 key candidate genes

| Ontology | ID | Description | GeneRatio | BgRatio | p-value | p.adjust | q-value | Gene ID | Count |
| --- | --- | --- | --- | --- | --- | --- | --- | --- | --- |
| BP | GO:0007059 | chromosome segregation | 30/221 | 424/18870 | 2.59E-15 | 6.67E-12 | 5.88E-12 | SYCP2/MYBL2/KIF11/TUBG2/KIF18B/CDC6/KIF2C/CENPI/ASPM/TPX2/SYCE2/UBE2C/KIF14/NUF2/BRIP1/CENPH/BUB1B/KIFC1/CHEK2/RCC2/CDCA2/SPC25/SASS6/TTK/REC8/CDT1/BIRC5/CENPE/BRCA1/HSPA1B | 30 |
| BP | GO:0098813 | nuclear chromosome segregation | 26/221 | 312/18870 | 4.50E-15 | 6.67E-12 | 5.88E-12 | SYCP2/MYBL2/KIF11/TUBG2/KIF18B/CDC6/KIF2C/CENPI/ASPM/TPX2/SYCE2/UBE2C/KIF14/NUF2/BRIP1/BUB1B/KIFC1/CHEK2/RCC2/SPC25/TTK/REC8/CDT1/BIRC5/CENPE/HSPA1B | 26 |
| BP | GO:0000280 | nuclear division | 29/221 | 441/18870 | 5.16E-14 | 5.09E-11 | 4.49E-11 | SYCP2/MYBL2/KIF11/TUBG2/KIF18B/KIF2C/CENPI/ASPM/TPX2/SYCE2/UBE2C/KIF14/ANLN/NUF2/BRIP1/BUB1B/KIFC1/CHEK2/EME1/CDCA2/SPC25/TTK/REC8/CDT1/INSR/BIRC5/CENPE/HSPA1B/WNT5A | 29 |
| BP | GO:0000070 | mitotic sister chromatid segregation | 19/221 | 184/18870 | 5.70E-13 | 3.94E-10 | 3.47E-10 | MYBL2/KIF11/TUBG2/KIF18B/KIF2C/CENPI/TPX2/UBE2C/KIF14/NUF2/BUB1B/KIFC1/CHEK2/SPC25/TTK/CDT1/BIRC5/CENPE/HSPA1B | 19 |
| BP | GO:0048285 | organelle fission | 29/221 | 488/18870 | 6.65E-13 | 3.94E-10 | 3.47E-10 | SYCP2/MYBL2/KIF11/TUBG2/KIF18B/KIF2C/CENPI/ASPM/TPX2/SYCE2/UBE2C/KIF14/ANLN/NUF2/BRIP1/BUB1B/KIFC1/CHEK2/EME1/CDCA2/SPC25/TTK/REC8/CDT1/INSR/BIRC5/CENPE/HSPA1B/WNT5A | 29 |
| BP | GO:0140014 | mitotic nuclear division | 22/221 | 274/18870 | 1.36E-12 | 6.72E-10 | 5.93E-10 | MYBL2/KIF11/TUBG2/KIF18B/KIF2C/CENPI/TPX2/UBE2C/KIF14/ANLN/NUF2/BUB1B/KIFC1/CHEK2/CDCA2/SPC25/TTK/CDT1/INSR/BIRC5/CENPE/HSPA1B | 22 |
| BP | GO:0000819 | sister chromatid segregation | 20/221 | 225/18870 | 2.29E-12 | 9.70E-10 | 8.56E-10 | MYBL2/KIF11/TUBG2/KIF18B/CDC6/KIF2C/CENPI/TPX2/UBE2C/KIF14/NUF2/BUB1B/KIFC1/CHEK2/SPC25/TTK/CDT1/BIRC5/CENPE/HSPA1B | 20 |
| BP | GO:1902850 | microtubule cytoskeleton organization involved in mitosis | 17/221 | 163/18870 | 8.40E-12 | 3.11E-09 | 2.74E-09 | MYBL2/KIF11/TUBG2/TPX2/NUF2/GJA1/CENPH/KIFC1/CHEK2/SPC25/SASS6/TTK/PLK3/CENPA/BIRC5/CENPE/HSPA1B | 17 |
| BP | GO:1901987 | regulation of cell cycle phase transition | 26/221 | 456/18870 | 2.85E-11 | 9.36E-09 | 8.26E-09 | CDKN2A/E2F7/CDC6/DTL/UBE2C/KIF14/ANLN/CDC25A/DONSON/NUF2/WEE1/BRIP1/RFWD3/BUB1B/CHEK2/EME1/RCC2/DBF4/SPC25/SASS6/TTK/PLK3/CDT1/BIRC5/CENPE/BRCA1 | 26 |
| BP | GO:0007052 | mitotic spindle organization | 15/221 | 130/18870 | 3.42E-11 | 1.01E-08 | 8.94E-09 | MYBL2/KIF11/TUBG2/TPX2/NUF2/CENPH/KIFC1/CHEK2/SPC25/SASS6/TTK/PLK3/BIRC5/CENPE/HSPA1B | 15 |
| BP | GO:0044772 | mitotic cell cycle phase transition | 26/221 | 470/18870 | 5.54E-11 | 1.49E-08 | 1.32E-08 | CDKN2A/E2F7/CDC6/DTL/IQGAP3/UBE2C/KIF14/ANLN/CDC25A/DONSON/NUF2/WEE1/MELK/RFWD3/BUB1B/CHEK2/RCC2/DBF4/SPC25/SASS6/TTK/PLK3/BIRC5/CENPE/BRCA1/POLE | 26 |
| BP | GO:1901990 | regulation of mitotic cell cycle phase transition | 22/221 | 355/18870 | 2.17E-10 | 5.35E-08 | 4.72E-08 | CDKN2A/E2F7/CDC6/DTL/UBE2C/KIF14/ANLN/CDC25A/DONSON/NUF2/WEE1/RFWD3/BUB1B/CHEK2/RCC2/SPC25/SASS6/TTK/PLK3/BIRC5/CENPE/BRCA1 | 22 |
| BP | GO:0007051 | spindle organization | 16/221 | 202/18870 | 2.13E-09 | 4.84E-07 | 4.27E-07 | MYBL2/KIF11/TUBG2/ASPM/TPX2/NUF2/CENPH/KIFC1/CHEK2/SPC25/SASS6/TTK/PLK3/BIRC5/CENPE/HSPA1B | 16 |
| BP | GO:0000075 | cell cycle checkpoint signaling | 15/221 | 192/18870 | 8.28E-09 | 1.75E-06 | 1.55E-06 | CDC6/DTL/DONSON/NUF2/BRIP1/RFWD3/BUB1B/CHEK2/EME1/SPC25/TTK/PLK3/CDT1/BIRC5/BRCA1 | 15 |
| BP | GO:0007093 | mitotic cell cycle checkpoint signaling | 13/221 | 142/18870 | 1.26E-08 | 2.49E-06 | 2.20E-06 | CDC6/DTL/DONSON/NUF2/RFWD3/BUB1B/CHEK2/EME1/SPC25/TTK/PLK3/BIRC5/BRCA1 | 13 |
| BP | GO:1901988 | negative regulation of cell cycle phase transition | 17/221 | 277/18870 | 3.07E-08 | 5.64E-06 | 4.97E-06 | E2F7/CDC6/DTL/DONSON/NUF2/WEE1/BRIP1/RFWD3/BUB1B/CHEK2/EME1/SPC25/TTK/PLK3/CDT1/BIRC5/BRCA1 | 17 |
| BP | GO:0006260 | DNA replication | 17/221 | 278/18870 | 3.23E-08 | 5.64E-06 | 4.97E-06 | LPIN1/E2F7/CHAF1B/GINS1/CDC6/DTL/FAM111B/DONSON/MCM7/RFWD3/EME1/DBF4/RFC5/CDT1/POLE2/BRCA1/POLE | 17 |
| BP | GO:0006261 | DNA-templated DNA replication | 13/221 | 161/18870 | 5.68E-08 | 9.35E-06 | 8.25E-06 | E2F7/GINS1/CDC6/DONSON/MCM7/RFWD3/EME1/DBF4/RFC5/CDT1/POLE2/BRCA1/POLE | 13 |
| BP | GO:1901991 | negative regulation of mitotic cell cycle phase transition | 14/221 | 196/18870 | 8.15E-08 | 1.27E-05 | 1.12E-05 | E2F7/CDC6/DTL/DONSON/NUF2/WEE1/RFWD3/BUB1B/CHEK2/SPC25/TTK/PLK3/BIRC5/BRCA1 | 14 |
| BP | GO:0010948 | negative regulation of cell cycle process | 17/221 | 320/18870 | 2.45E-07 | 3.47E-05 | 3.06E-05 | E2F7/CDC6/DTL/DONSON/NUF2/WEE1/BRIP1/RFWD3/BUB1B/CHEK2/EME1/SPC25/TTK/PLK3/CDT1/BIRC5/BRCA1 | 17 |
| BP | GO:0045930 | negative regulation of mitotic cell cycle | 15/221 | 248/18870 | 2.46E-07 | 3.47E-05 | 3.06E-05 | E2F7/CDC6/DTL/DONSON/NUF2/WEE1/RFWD3/BUB1B/CHEK2/EME1/SPC25/TTK/PLK3/BIRC5/BRCA1 | 15 |
| BP | GO:0045786 | negative regulation of cell cycle | 19/221 | 407/18870 | 3.49E-07 | 4.70E-05 | 4.14E-05 | CDKN2A/E2F7/CDC6/DTL/TP53BP2/DONSON/NUF2/WEE1/BRIP1/RFWD3/BUB1B/CHEK2/EME1/SPC25/TTK/PLK3/CDT1/BIRC5/BRCA1 | 19 |
| BP | GO:0051983 | regulation of chromosome segregation | 11/221 | 131/18870 | 4.11E-07 | 5.29E-05 | 4.67E-05 | CDC6/KIF2C/UBE2C/NUF2/BUB1B/RCC2/CDCA2/SPC25/TTK/BIRC5/CENPE | 11 |
| BP | GO:0000086 | G2/M transition of mitotic cell cycle | 11/221 | 140/18870 | 8.01E-07 | 9.89E-05 | 8.72E-05 | CDC6/DTL/KIF14/CDC25A/DONSON/WEE1/MELK/CHEK2/RCC2/PLK3/BRCA1 | 11 |
| BP | GO:0008608 | attachment of spindle microtubules to kinetochore | 7/221 | 49/18870 | 1.55E-06 | 0.000184184 | 0.000162459 | KIF2C/NUF2/RCC2/SPC25/CDT1/BIRC5/CENPE | 7 |
| BP | GO:0051310 | metaphase chromosome alignment | 9/221 | 96/18870 | 1.89E-06 | 0.000215265 | 0.000189875 | KIF2C/KIF14/NUF2/KIFC1/RCC2/SPC25/CDT1/BIRC5/CENPE | 9 |
| BP | GO:0045787 | positive regulation of cell cycle | 16/221 | 334/18870 | 2.17E-06 | 0.000231829 | 0.000204485 | E2F7/CIT/CDC6/DTL/UBE2C/TBX3/KIF14/CDC25A/TAL1/RCC2/DBF4/SASS6/INSR/BIRC5/FOXA1/WNT5A | 16 |
| BP | GO:0044839 | cell cycle G2/M phase transition | 11/221 | 155/18870 | 2.19E-06 | 0.000231829 | 0.000204485 | CDC6/DTL/KIF14/CDC25A/DONSON/WEE1/MELK/CHEK2/RCC2/PLK3/BRCA1 | 11 |
| BP | GO:0051315 | attachment of mitotic spindle microtubules to kinetochore | 5/221 | 21/18870 | 3.68E-06 | 0.000375642 | 0.000331335 | KIF2C/NUF2/CDT1/BIRC5/CENPE | 5 |
| BP | GO:0031570 | DNA integrity checkpoint signaling | 10/221 | 135/18870 | 4.36E-06 | 0.000422278 | 0.00037247 | CDC6/DTL/DONSON/BRIP1/RFWD3/CHEK2/EME1/PLK3/CDT1/BRCA1 | 10 |
| BP | GO:0007080 | mitotic metaphase chromosome alignment | 7/221 | 57/18870 | 4.42E-06 | 0.000422278 | 0.00037247 | KIF2C/KIF14/NUF2/KIFC1/CDT1/BIRC5/CENPE | 7 |
| BP | GO:0051303 | establishment of chromosome localization | 9/221 | 108/18870 | 5.05E-06 | 0.000467377 | 0.00041225 | KIF2C/KIF14/NUF2/KIFC1/RCC2/SPC25/CDT1/BIRC5/CENPE | 9 |
| BP | GO:0051321 | meiotic cell cycle | 14/221 | 287/18870 | 7.76E-06 | 0.000696108 | 0.000614002 | SYCP2/SMC1B/TUBG2/ASPM/SYCE2/EXO1/CDC25A/NUF2/BRIP1/EME1/TTK/REC8/INSR/WNT5A | 14 |
| BP | GO:0044774 | mitotic DNA integrity checkpoint signaling | 8/221 | 87/18870 | 8.23E-06 | 0.000717375 | 0.00063276 | CDC6/DTL/DONSON/RFWD3/CHEK2/EME1/PLK3/BRCA1 | 8 |
| BP | GO:0000082 | G1/S transition of mitotic cell cycle | 13/221 | 252/18870 | 9.07E-06 | 0.000746883 | 0.000658788 | CDKN2A/E2F7/CDC6/IQGAP3/KIF14/CDC25A/WEE1/RFWD3/CHEK2/DBF4/SASS6/PLK3/POLE | 13 |
| BP | GO:0050000 | chromosome localization | 9/221 | 116/18870 | 9.08E-06 | 0.000746883 | 0.000658788 | KIF2C/KIF14/NUF2/KIFC1/RCC2/SPC25/CDT1/BIRC5/CENPE | 9 |
| BP | GO:0030071 | regulation of mitotic metaphase/anaphase transition | 8/221 | 90/18870 | 1.06E-05 | 0.000848593 | 0.000748501 | CDC6/UBE2C/NUF2/BUB1B/SPC25/TTK/BIRC5/CENPE | 8 |
| BP | GO:1902099 | regulation of metaphase/anaphase transition of cell cycle | 8/221 | 93/18870 | 1.35E-05 | 0.001043456 | 0.000920381 | CDC6/UBE2C/NUF2/BUB1B/SPC25/TTK/BIRC5/CENPE | 8 |
| BP | GO:0090068 | positive regulation of cell cycle process | 13/221 | 262/18870 | 1.37E-05 | 0.001043456 | 0.000920381 | E2F7/CIT/CDC6/DTL/UBE2C/KIF14/CDC25A/RCC2/DBF4/SASS6/INSR/BIRC5/WNT5A | 13 |
| BP | GO:0007091 | metaphase/anaphase transition of mitotic cell cycle | 8/221 | 94/18870 | 1.46E-05 | 0.001082959 | 0.000955223 | CDC6/UBE2C/NUF2/BUB1B/SPC25/TTK/BIRC5/CENPE | 8 |
| BP | GO:0044784 | metaphase/anaphase transition of cell cycle | 8/221 | 97/18870 | 1.84E-05 | 0.001331133 | 0.001174126 | CDC6/UBE2C/NUF2/BUB1B/SPC25/TTK/BIRC5/CENPE | 8 |
| BP | GO:0090307 | mitotic spindle assembly | 7/221 | 71/18870 | 1.94E-05 | 0.001364732 | 0.001203762 | MYBL2/KIF11/TPX2/KIFC1/CHEK2/BIRC5/HSPA1B | 7 |
| BP | GO:0044843 | cell cycle G1/S phase transition | 13/221 | 279/18870 | 2.66E-05 | 0.001834066 | 0.001617738 | CDKN2A/E2F7/CDC6/IQGAP3/KIF14/CDC25A/WEE1/RFWD3/CHEK2/DBF4/SASS6/PLK3/POLE | 13 |
| BP | GO:0051225 | spindle assembly | 9/221 | 133/18870 | 2.73E-05 | 0.001837425 | 0.001620701 | MYBL2/KIF11/ASPM/TPX2/KIFC1/CHEK2/SASS6/BIRC5/HSPA1B | 9 |
| BP | GO:0033045 | regulation of sister chromatid segregation | 8/221 | 104/18870 | 3.06E-05 | 0.002015373 | 0.001777659 | CDC6/UBE2C/NUF2/BUB1B/SPC25/TTK/BIRC5/CENPE | 8 |
| BP | GO:0051656 | establishment of organelle localization | 17/221 | 469/18870 | 4.03E-05 | 0.002593975 | 0.002288015 | KIF2C/MYO7B/KIF14/NUF2/GJA1/KIFC1/RCC2/SPC25/SDC4/CDT1/CENPA/BIRC5/CENPE/FCER1A/SLC18A2/FGR/GATA2 | 17 |
| BP | GO:1903046 | meiotic cell cycle process | 11/221 | 214/18870 | 4.62E-05 | 0.002910821 | 0.00256749 | SYCP2/TUBG2/ASPM/SYCE2/CDC25A/NUF2/BRIP1/EME1/TTK/REC8/WNT5A | 11 |
| BP | GO:0044773 | mitotic DNA damage checkpoint signaling | 7/221 | 83/18870 | 5.37E-05 | 0.003310879 | 0.00292036 | DTL/DONSON/RFWD3/CHEK2/EME1/PLK3/BRCA1 | 7 |
| BP | GO:0051783 | regulation of nuclear division | 9/221 | 146/18870 | 5.68E-05 | 0.003435531 | 0.003030309 | UBE2C/NUF2/BUB1B/CDCA2/SPC25/TTK/INSR/BIRC5/WNT5A | 9 |
| BP | GO:0010965 | regulation of mitotic sister chromatid separation | 6/221 | 59/18870 | 6.47E-05 | 0.003834772 | 0.00338246 | UBE2C/NUF2/BUB1B/SPC25/TTK/BIRC5 | 6 |
| BP | GO:0051302 | regulation of cell division | 10/221 | 186/18870 | 7.04E-05 | 0.004090373 | 0.003607913 | E2F7/CIT/KIF18B/CDC6/ASPM/KIF20A/KIF14/TAL1/PLK3/BIRC5 | 10 |
| BP | GO:0007088 | regulation of mitotic nuclear division | 8/221 | 118/18870 | 7.56E-05 | 0.004303921 | 0.003796272 | UBE2C/NUF2/BUB1B/CDCA2/SPC25/TTK/INSR/BIRC5 | 8 |
| BP | GO:0000910 | cytokinesis | 10/221 | 188/18870 | 7.70E-05 | 0.004303921 | 0.003796272 | E2F7/CIT/CDC6/IQGAP3/KIF20A/KIF14/ANLN/PLK3/CENPA/BIRC5 | 10 |
| BP | GO:0031589 | cell-substrate adhesion | 14/221 | 356/18870 | 8.30E-05 | 0.004553424 | 0.004016347 | CDKN2A/CD36/THY1/KIF14/SMAD3/RCC2/ITGA2/CORO1A/GBP1/CCDC80/SDC4/ITGB6/SORBS1/EGFLAM | 14 |
| BP | GO:0051306 | mitotic sister chromatid separation | 6/221 | 62/18870 | 8.58E-05 | 0.004619586 | 0.004074705 | UBE2C/NUF2/BUB1B/SPC25/TTK/BIRC5 | 6 |
| BP | GO:0051383 | kinetochore organization | 4/221 | 21/18870 | 9.37E-05 | 0.004956404 | 0.004371795 | NUF2/CENPH/CENPA/CENPE | 4 |
| BP | GO:0045132 | meiotic chromosome segregation | 7/221 | 91/18870 | 9.66E-05 | 0.005022359 | 0.004429971 | SYCP2/ASPM/SYCE2/NUF2/BRIP1/TTK/REC8 | 7 |
| BP | GO:0140694 | non-membrane-bounded organelle assembly | 15/221 | 408/18870 | 9.85E-05 | 0.005028506 | 0.004435393 | MYBL2/KIF11/ASPM/TPX2/ANLN/CENPH/KIFC1/CHEK2/SASS6/CENPA/BIRC5/CENPE/BRIX1/BRCA1/HSPA1B | 15 |
| BP | GO:0032465 | regulation of cytokinesis | 7/221 | 93/18870 | 0.000110912 | 0.005568152 | 0.004911387 | E2F7/CIT/CDC6/KIF20A/KIF14/PLK3/BIRC5 | 7 |
| BP | GO:0000077 | DNA damage checkpoint signaling | 8/221 | 126/18870 | 0.00011984 | 0.005916081 | 0.005218278 | DTL/DONSON/BRIP1/RFWD3/CHEK2/EME1/PLK3/BRCA1 | 8 |
| BP | GO:0045931 | positive regulation of mitotic cell cycle | 8/221 | 128/18870 | 0.000133728 | 0.006493461 | 0.005727556 | CDC6/DTL/UBE2C/CDC25A/TAL1/RCC2/SASS6/FOXA1 | 8 |
| BP | GO:0044786 | cell cycle DNA replication | 5/221 | 44/18870 | 0.000157663 | 0.007532227 | 0.006643799 | E2F7/GINS1/DONSON/DBF4/CDT1 | 5 |
| BP | GO:0007019 | microtubule depolymerization | 5/221 | 45/18870 | 0.000175695 | 0.008260431 | 0.007286112 | KIF18B/KIF2C/TPX2/KIF24/STMN2 | 5 |
| BP | GO:0120162 | positive regulation of cold-induced thermogenesis | 7/221 | 101/18870 | 0.000186086 | 0.008612293 | 0.007596472 | YBX2/LPIN1/CD36/TRPV2/GJA1/PER2/HADH | 7 |
| BP | GO:0007094 | mitotic spindle assembly checkpoint signaling | 5/221 | 46/18870 | 0.000195258 | 0.008632154 | 0.00761399 | NUF2/BUB1B/SPC25/TTK/BIRC5 | 5 |
| BP | GO:0071173 | spindle assembly checkpoint signaling | 5/221 | 46/18870 | 0.000195258 | 0.008632154 | 0.00761399 | NUF2/BUB1B/SPC25/TTK/BIRC5 | 5 |
| BP | GO:0071174 | mitotic spindle checkpoint signaling | 5/221 | 46/18870 | 0.000195258 | 0.008632154 | 0.00761399 | NUF2/BUB1B/SPC25/TTK/BIRC5 | 5 |
| BP | GO:0031577 | spindle checkpoint signaling | 5/221 | 47/18870 | 0.000216439 | 0.009291188 | 0.008195291 | NUF2/BUB1B/SPC25/TTK/BIRC5 | 5 |
| BP | GO:0090329 | regulation of DNA-templated DNA replication | 5/221 | 47/18870 | 0.000216439 | 0.009291188 | 0.008195291 | E2F7/MCM7/DBF4/RFC5/CDT1 | 5 |
| BP | GO:0010389 | regulation of G2/M transition of mitotic cell cycle | 7/221 | 104/18870 | 0.00022314 | 0.009397231 | 0.008288826 | CDC6/DTL/KIF14/CDC25A/DONSON/RCC2/BRCA1 | 7 |
| BP | GO:1905818 | regulation of chromosome separation | 6/221 | 74/18870 | 0.000230053 | 0.009397231 | 0.008288826 | UBE2C/NUF2/BUB1B/SPC25/TTK/BIRC5 | 6 |
| BP | GO:0033046 | negative regulation of sister chromatid segregation | 5/221 | 48/18870 | 0.000239324 | 0.009397231 | 0.008288826 | NUF2/BUB1B/SPC25/TTK/BIRC5 | 5 |
| BP | GO:0033048 | negative regulation of mitotic sister chromatid segregation | 5/221 | 48/18870 | 0.000239324 | 0.009397231 | 0.008288826 | NUF2/BUB1B/SPC25/TTK/BIRC5 | 5 |
| BP | GO:0045841 | negative regulation of mitotic metaphase/anaphase transition | 5/221 | 48/18870 | 0.000239324 | 0.009397231 | 0.008288826 | NUF2/BUB1B/SPC25/TTK/BIRC5 | 5 |
| BP | GO:2000816 | negative regulation of mitotic sister chromatid separation | 5/221 | 48/18870 | 0.000239324 | 0.009397231 | 0.008288826 | NUF2/BUB1B/SPC25/TTK/BIRC5 | 5 |
| BP | GO:0048732 | gland development | 15/221 | 443/18870 | 0.000241117 | 0.009397231 | 0.008288826 | E2F7/IGFBP5/IQGAP3/TBX3/CRHR1/SMAD3/HOXA9/ITGA2/INSR/MMP2/GDF7/FOXA1/DEAF1/WNT5A/GATA2 | 15 |
| BP | GO:0048332 | mesoderm morphogenesis | 6/221 | 77/18870 | 0.000285991 | 0.010758305 | 0.009489361 | TBX3/TAL1/GJA1/SMAD3/ITGA2/WNT5A | 6 |
| BP | GO:0051985 | negative regulation of chromosome segregation | 5/221 | 50/18870 | 0.000290569 | 0.010758305 | 0.009489361 | NUF2/BUB1B/SPC25/TTK/BIRC5 | 5 |
| BP | GO:1902100 | negative regulation of metaphase/anaphase transition of cell cycle | 5/221 | 50/18870 | 0.000290569 | 0.010758305 | 0.009489361 | NUF2/BUB1B/SPC25/TTK/BIRC5 | 5 |
| BP | GO:1905819 | negative regulation of chromosome separation | 5/221 | 50/18870 | 0.000290569 | 0.010758305 | 0.009489361 | NUF2/BUB1B/SPC25/TTK/BIRC5 | 5 |
| BP | GO:2000045 | regulation of G1/S transition of mitotic cell cycle | 9/221 | 183/18870 | 0.000315019 | 0.011519599 | 0.01016086 | CDKN2A/E2F7/CDC6/KIF14/WEE1/RFWD3/CHEK2/SASS6/PLK3 | 9 |
| BP | GO:0051304 | chromosome separation | 6/221 | 81/18870 | 0.000376586 | 0.013591813 | 0.011988656 | UBE2C/NUF2/BUB1B/SPC25/TTK/BIRC5 | 6 |
| BP | GO:0120161 | regulation of cold-induced thermogenesis | 8/221 | 150/18870 | 0.000394956 | 0.013591813 | 0.011988656 | YBX2/LPIN1/CD36/TRPV2/GJA1/PER2/HADH/NOVA2 | 8 |
| BP | GO:0034508 | centromere complex assembly | 4/221 | 30/18870 | 0.000395067 | 0.013591813 | 0.011988656 | CENPI/CENPH/CENPA/CENPE | 4 |
| BP | GO:0042770 | signal transduction in response to DNA damage | 9/221 | 189/18870 | 0.000399098 | 0.013591813 | 0.011988656 | E2F7/DTL/DONSON/BRIP1/RFWD3/CHEK2/EME1/PLK3/BRCA1 | 9 |
| BP | GO:0106106 | cold-induced thermogenesis | 8/221 | 151/18870 | 0.000412923 | 0.013591813 | 0.011988656 | YBX2/LPIN1/CD36/TRPV2/GJA1/PER2/HADH/NOVA2 | 8 |
| BP | GO:0048853 | forebrain morphogenesis | 3/221 | 13/18870 | 0.000415596 | 0.013591813 | 0.011988656 | OTX1/GDF7/WNT5A | 3 |
| BP | GO:0060100 | positive regulation of phagocytosis, engulfment | 3/221 | 13/18870 | 0.000415596 | 0.013591813 | 0.011988656 | CD36/ITGA2/GATA2 | 3 |
| BP | GO:1905155 | positive regulation of membrane invagination | 3/221 | 13/18870 | 0.000415596 | 0.013591813 | 0.011988656 | CD36/ITGA2/GATA2 | 3 |
| BP | GO:2000105 | positive regulation of DNA-templated DNA replication | 3/221 | 13/18870 | 0.000415596 | 0.013591813 | 0.011988656 | E2F7/DBF4/CDT1 | 3 |
| BP | GO:0033047 | regulation of mitotic sister chromatid segregation | 5/221 | 54/18870 | 0.000417574 | 0.013591813 | 0.011988656 | NUF2/BUB1B/SPC25/TTK/BIRC5 | 5 |
| BP | GO:1902749 | regulation of cell cycle G2/M phase transition | 7/221 | 116/18870 | 0.000435207 | 0.014011757 | 0.012359068 | CDC6/DTL/KIF14/CDC25A/DONSON/RCC2/BRCA1 | 7 |
| BP | GO:0140013 | meiotic nuclear division | 9/221 | 194/18870 | 0.000482562 | 0.015269752 | 0.013468682 | SYCP2/ASPM/SYCE2/NUF2/BRIP1/EME1/TTK/REC8/WNT5A | 9 |
| BP | GO:0048041 | focal adhesion assembly | 6/221 | 85/18870 | 0.000488208 | 0.015269752 | 0.013468682 | THY1/SMAD3/RCC2/ITGA2/SDC4/SORBS1 | 6 |
| BP | GO:0045005 | DNA-templated DNA replication maintenance of fidelity | 5/221 | 56/18870 | 0.000494901 | 0.015269752 | 0.013468682 | DONSON/RFWD3/EME1/BRCA1/POLE | 5 |
| BP | GO:0045839 | negative regulation of mitotic nuclear division | 5/221 | 56/18870 | 0.000494901 | 0.015269752 | 0.013468682 | NUF2/BUB1B/SPC25/TTK/BIRC5 | 5 |
| BP | GO:0000281 | mitotic cytokinesis | 6/221 | 88/18870 | 0.000587634 | 0.017944047 | 0.015827543 | CIT/IQGAP3/KIF20A/ANLN/CENPA/BIRC5 | 6 |
| BP | GO:0006275 | regulation of DNA replication | 7/221 | 124/18870 | 0.000649464 | 0.018870594 | 0.016644805 | LPIN1/E2F7/CDC6/MCM7/DBF4/RFC5/CDT1 | 7 |
| BP | GO:0006271 | DNA strand elongation involved in DNA replication | 3/221 | 15/18870 | 0.000649831 | 0.018870594 | 0.016644805 | GINS1/MCM7/POLE | 3 |
| BP | GO:0007567 | parturition | 3/221 | 15/18870 | 0.000649831 | 0.018870594 | 0.016644805 | CRHR1/PLA2G4B/MMP2 | 3 |
| BP | GO:0060099 | regulation of phagocytosis, engulfment | 3/221 | 15/18870 | 0.000649831 | 0.018870594 | 0.016644805 | CD36/ITGA2/GATA2 | 3 |
| BP | GO:1905153 | regulation of membrane invagination | 3/221 | 15/18870 | 0.000649831 | 0.018870594 | 0.016644805 | CD36/ITGA2/GATA2 | 3 |
| BP | GO:0007292 | female gamete generation | 8/221 | 163/18870 | 0.000685286 | 0.01970695 | 0.017382512 | SYCP2/YBX2/ASPM/HROB/PAQR8/TTK/REC8/MMP2 | 8 |
| BP | GO:0001704 | formation of primary germ layer | 7/221 | 128/18870 | 0.000784142 | 0.02233298 | 0.019698801 | TAL1/GJA1/SMAD3/ITGA2/MMP2/ETS2/WNT5A | 7 |
| BP | GO:0032731 | positive regulation of interleukin-1 beta production | 5/221 | 62/18870 | 0.000792128 | 0.022345547 | 0.019709886 | CD36/SMAD3/LILRA2/NLRP12/WNT5A | 5 |
| BP | GO:1902806 | regulation of cell cycle G1/S phase transition | 9/221 | 209/18870 | 0.000822855 | 0.022993354 | 0.020281284 | CDKN2A/E2F7/CDC6/KIF14/WEE1/RFWD3/CHEK2/SASS6/PLK3 | 9 |
| BP | GO:1990845 | adaptive thermogenesis | 8/221 | 168/18870 | 0.000834778 | 0.023108525 | 0.02038287 | YBX2/LPIN1/CD36/TRPV2/GJA1/PER2/HADH/NOVA2 | 8 |
| BP | GO:0051784 | negative regulation of nuclear division | 5/221 | 63/18870 | 0.000852321 | 0.023375682 | 0.020618516 | NUF2/BUB1B/SPC25/TTK/BIRC5 | 5 |
| BP | GO:0007044 | cell-substrate junction assembly | 6/221 | 95/18870 | 0.000880735 | 0.023933364 | 0.021110419 | THY1/SMAD3/RCC2/ITGA2/SDC4/SORBS1 | 6 |
| BP | GO:0003161 | cardiac conduction system development | 4/221 | 37/18870 | 0.000893034 | 0.024046967 | 0.021210623 | TBX3/GJA1/DSG2/CACNA1G | 4 |
| BP | GO:0032274 | gonadotropin secretion | 3/221 | 17/18870 | 0.000954531 | 0.025243949 | 0.022266421 | TBX3/GJA1/FOXD1 | 3 |
| BP | GO:0051382 | kinetochore assembly | 3/221 | 17/18870 | 0.000954531 | 0.025243949 | 0.022266421 | CENPH/CENPA/CENPE | 3 |
| BP | GO:0048608 | reproductive structure development | 11/221 | 305/18870 | 0.000977389 | 0.025473731 | 0.0224691 | SYCP2/ASPM/TBX3/BRIP1/HOXA9/REC8/INSR/MMP2/GDF7/FOXA1/WNT5A | 11 |
| BP | GO:0030879 | mammary gland development | 7/221 | 133/18870 | 0.000982538 | 0.025473731 | 0.0224691 | IGFBP5/IQGAP3/TBX3/HOXA9/ITGA2/DEAF1/WNT5A | 7 |
| BP | GO:0045740 | positive regulation of DNA replication | 4/221 | 38/18870 | 0.000989021 | 0.025473731 | 0.0224691 | LPIN1/E2F7/DBF4/CDT1 | 4 |
| BP | GO:0031109 | microtubule polymerization or depolymerization | 7/221 | 134/18870 | 0.001026575 | 0.026213054 | 0.02312122 | TUBG2/KIF18B/KIF2C/TPX2/KIF24/STMN2/HSPA1B | 7 |
| BP | GO:1901992 | positive regulation of mitotic cell cycle phase transition | 6/221 | 98/18870 | 0.001036237 | 0.02622029 | 0.023127602 | CDC6/DTL/UBE2C/CDC25A/RCC2/SASS6 | 6 |
| BP | GO:0046661 | male sex differentiation | 8/221 | 174/18870 | 0.001047619 | 0.02622029 | 0.023127602 | SYCP2/ASPM/TBX3/BRIP1/HOXA9/REC8/INSR/WNT5A | 8 |
| BP | GO:0051445 | regulation of meiotic cell cycle | 5/221 | 66/18870 | 0.001053415 | 0.02622029 | 0.023127602 | ASPM/CDC25A/TTK/INSR/WNT5A | 5 |
| BP | GO:0061458 | reproductive system development | 11/221 | 309/18870 | 0.001085814 | 0.026801508 | 0.023640266 | SYCP2/ASPM/TBX3/BRIP1/HOXA9/REC8/INSR/MMP2/GDF7/FOXA1/WNT5A | 11 |
| BP | GO:0007498 | mesoderm development | 7/221 | 136/18870 | 0.001119299 | 0.027378356 | 0.024149074 | TBX3/TAL1/GJA1/SMAD3/ITGA2/ETS2/WNT5A | 7 |
| BP | GO:0001655 | urogenital system development | 5/221 | 67/18870 | 0.00112767 | 0.027378356 | 0.024149074 | HOXA9/MMP2/FOXA1/WNT5A/GATA2 | 5 |
| BP | GO:0010810 | regulation of cell-substrate adhesion | 9/221 | 220/18870 | 0.001179888 | 0.028264664 | 0.024930842 | CDKN2A/CD36/THY1/SMAD3/RCC2/GBP1/CCDC80/SDC4/EGFLAM | 9 |
| BP | GO:0000731 | DNA synthesis involved in DNA repair | 4/221 | 40/18870 | 0.001202346 | 0.028264664 | 0.024930842 | DTL/HROB/POLE2/POLE | 4 |
| BP | GO:0033260 | nuclear DNA replication | 4/221 | 40/18870 | 0.001202346 | 0.028264664 | 0.024930842 | GINS1/DONSON/DBF4/CDT1 | 4 |
| BP | GO:0071459 | protein localization to chromosome, centromeric region | 4/221 | 40/18870 | 0.001202346 | 0.028264664 | 0.024930842 | BUB1B/RCC2/TTK/CENPA | 4 |
| BP | GO:0036293 | response to decreased oxygen levels | 11/221 | 315/18870 | 0.001266927 | 0.0295134 | 0.026032289 | ERO1A/NPEPPS/VASN/PLEKHN1/SMAD3/BRIP1/ITGA2/PLK3/CPEB4/MMP2/CA9 | 11 |
| BP | GO:0150115 | cell-substrate junction organization | 6/221 | 102/18870 | 0.001275393 | 0.0295134 | 0.026032289 | THY1/SMAD3/RCC2/ITGA2/SDC4/SORBS1 | 6 |
| BP | GO:0001659 | temperature homeostasis | 8/221 | 183/18870 | 0.001446117 | 0.033204648 | 0.029288154 | YBX2/LPIN1/CD36/TRPV2/GJA1/PER2/HADH/NOVA2 | 8 |
| BP | GO:0060216 | definitive hemopoiesis | 3/221 | 20/18870 | 0.001559338 | 0.035528907 | 0.031338266 | TAL1/HOXA9/GATA2 | 3 |
| BP | GO:0032732 | positive regulation of interleukin-1 production | 5/221 | 73/18870 | 0.001657341 | 0.037473617 | 0.033053598 | CD36/SMAD3/LILRA2/NLRP12/WNT5A | 5 |
| BP | GO:0001707 | mesoderm formation | 5/221 | 75/18870 | 0.001868848 | 0.041935813 | 0.036989476 | TAL1/GJA1/SMAD3/ITGA2/WNT5A | 5 |
| BP | GO:0007229 | integrin-mediated signaling pathway | 6/221 | 111/18870 | 0.001966405 | 0.043793165 | 0.038627754 | THY1/MPIG6B/RCC2/ITGA2/ITGB6/FGR | 6 |
| BP | GO:0086001 | cardiac muscle cell action potential | 5/221 | 76/18870 | 0.001981705 | 0.043804555 | 0.0386378 | SLC4A3/NUP155/GJA1/DSG2/CACNA1G | 5 |
| BP | GO:0007160 | cell-matrix adhesion | 9/221 | 239/18870 | 0.002084279 | 0.045730622 | 0.040336687 | CDKN2A/CD36/THY1/SMAD3/RCC2/ITGA2/SDC4/ITGB6/SORBS1 | 9 |
| BP | GO:0007548 | sex differentiation | 10/221 | 288/18870 | 0.002176434 | 0.047401451 | 0.041810442 | SYCP2/CENPI/ASPM/TBX3/BRIP1/HOXA9/REC8/INSR/MMP2/WNT5A | 10 |
| BP | GO:0032467 | positive regulation of cytokinesis | 4/221 | 47/18870 | 0.002201571 | 0.047598925 | 0.041984623 | CIT/CDC6/KIF14/BIRC5 | 4 |
| CC | GO:0000793 | condensed chromosome | 19/229 | 281/19886 | 7.14E-10 | 2.09E-07 | 1.80E-07 | SYCP2/SMC1B/KIF2C/CENPI/SYCE2/ERCC6L/NUF2/CENPH/BUB1B/RASSF2/SPC25/TTK/PLK3/REC8/CDT1/CENPA/BIRC5/CENPE/BRCA1 | 19 |
| CC | GO:0000779 | condensed chromosome, centromeric region | 15/229 | 182/19886 | 3.23E-09 | 4.73E-07 | 4.06E-07 | KIF2C/CENPI/ERCC6L/NUF2/CENPH/BUB1B/RASSF2/SPC25/TTK/PLK3/REC8/CDT1/CENPA/BIRC5/CENPE | 15 |
| CC | GO:0000775 | chromosome, centromeric region | 17/229 | 258/19886 | 8.54E-09 | 8.34E-07 | 7.16E-07 | SMC1B/KIF2C/CENPI/ERCC6L/NUF2/CENPH/BUB1B/RCC2/RASSF2/SPC25/TTK/PLK3/REC8/CDT1/CENPA/BIRC5/CENPE | 17 |
| CC | GO:0000776 | kinetochore | 14/229 | 171/19886 | 1.19E-08 | 8.73E-07 | 7.49E-07 | KIF2C/CENPI/ERCC6L/NUF2/CENPH/BUB1B/RASSF2/SPC25/TTK/PLK3/REC8/CDT1/BIRC5/CENPE | 14 |
| CC | GO:0098687 | chromosomal region | 19/229 | 399/19886 | 2.03E-07 | 1.19E-05 | 1.02E-05 | SMC1B/KIF2C/CENPI/ERCC6L/MCM7/NUF2/CENPH/BUB1B/CHEK2/RCC2/RASSF2/SPC25/TTK/PLK3/REC8/CDT1/CENPA/BIRC5/CENPE | 19 |
| CC | GO:0005819 | spindle | 18/229 | 431/19886 | 2.80E-06 | 0.000136783 | 0.000117446 | KIF11/TUBG2/KIF18B/CDC6/KIF2C/ASPM/TUBB2B/TPX2/PLEKHG6/KIF20A/KIF14/BUB1B/KIFC1/RCC2/TTK/PLK3/BIRC5/CENPE | 18 |
| CC | GO:0000800 | lateral element | 4/229 | 14/19886 | 1.57E-05 | 0.000578891 | 0.000497054 | SYCP2/SMC1B/REC8/BRCA1 | 4 |
| CC | GO:0005871 | kinesin complex | 6/229 | 47/19886 | 1.58E-05 | 0.000578891 | 0.000497054 | KIF11/KIF18B/KIF2C/KIF20A/KIF14/KIFC1 | 6 |
| CC | GO:0051233 | spindle midzone | 5/229 | 37/19886 | 6.26E-05 | 0.002036817 | 0.001748875 | KIF18B/CDC6/KIF14/RCC2/CENPE | 5 |
| CC | GO:0000228 | nuclear chromosome | 11/229 | 228/19886 | 7.08E-05 | 0.002075367 | 0.001781975 | SYCP2/SMC1B/GINS1/SYCE2/MCM7/EME1/REC8/BIRC5/POLE2/BRCA1/POLE | 11 |
| CC | GO:0000795 | synaptonemal complex | 5/229 | 40/19886 | 9.18E-05 | 0.002242159 | 0.001925188 | SYCP2/SMC1B/SYCE2/REC8/BRCA1 | 5 |
| CC | GO:0099086 | synaptonemal structure | 5/229 | 40/19886 | 9.18E-05 | 0.002242159 | 0.001925188 | SYCP2/SMC1B/SYCE2/REC8/BRCA1 | 5 |
| CC | GO:0005874 | microtubule | 16/229 | 467/19886 | 0.000109061 | 0.002458059 | 0.002110566 | KIF11/TUBG2/KIF18B/KIF2C/ASPM/TUBB2B/NINL/TPX2/KIF24/KIF20A/KIF14/KIFC1/RCC2/BIRC5/CENPE/CLIP4 | 16 |
| CC | GO:0072686 | mitotic spindle | 9/229 | 184/19886 | 0.000291145 | 0.006093244 | 0.005231849 | KIF11/KIF18B/CDC6/ASPM/TUBB2B/TPX2/KIFC1/RCC2/CENPE | 9 |
| CC | GO:1990023 | mitotic spindle midzone | 3/229 | 14/19886 | 0.000499495 | 0.009756801 | 0.008377494 | KIF18B/RCC2/CENPE | 3 |
| CC | GO:1990752 | microtubule end | 4/229 | 34/19886 | 0.000605682 | 0.011091554 | 0.009523555 | KIF18B/KIF2C/ASPM/CLIP4 | 4 |
| CC | GO:0000940 | outer kinetochore | 3/229 | 20/19886 | 0.001486583 | 0.025621689 | 0.021999582 | NUF2/BUB1B/SPC25 | 3 |
| CC | GO:0009897 | external side of plasma membrane | 12/229 | 387/19886 | 0.001842918 | 0.029998617 | 0.025757749 | CD36/THY1/CCR3/SLC4A3/ANPEP/ITGA2/CSF3R/INSR/ITGB6/ASGR2/IL12RB2/STAB2 | 12 |
| CC | GO:0005875 | microtubule associated complex | 7/229 | 156/19886 | 0.002238831 | 0.033979514 | 0.029175872 | KIF11/KIF18B/KIF2C/KIF20A/KIF14/KIFC1/BIRC5 | 7 |
| CC | GO:0030666 | endocytic vesicle membrane | 8/229 | 202/19886 | 0.002423947 | 0.033979514 | 0.029175872 | CD36/COLEC12/CSF3R/GRIA3/SYT7/CORO1A/WNT5A/STAB2 | 8 |
| CC | GO:0000794 | condensed nuclear chromosome | 5/229 | 81/19886 | 0.002441458 | 0.033979514 | 0.029175872 | SYCP2/SMC1B/SYCE2/REC8/BRCA1 | 5 |
| CC | GO:0035371 | microtubule plus-end | 3/229 | 24/19886 | 0.002551363 | 0.033979514 | 0.029175872 | KIF18B/KIF2C/CLIP4 | 3 |
| MF | GO:0003777 | microtubule motor activity | 8/222 | 68/18496 | 1.51E-06 | 0.000714946 | 0.0006412 | KIF11/KIF18B/KIF2C/KIF24/KIF20A/KIF14/KIFC1/CENPE | 8 |
| MF | GO:0003774 | cytoskeletal motor activity | 9/222 | 112/18496 | 8.28E-06 | 0.001958788 | 0.001756741 | KIF11/KIF18B/KIF2C/MYO7B/KIF24/KIF20A/KIF14/KIFC1/CENPE | 9 |
| MF | GO:0038024 | cargo receptor activity | 6/222 | 64/18496 | 0.000117242 | 0.018485111 | 0.01657839 | CD36/SCARF2/COLEC12/INSR/ASGR2/STAB2 | 6 |
| MF | GO:0008235 | metalloexopeptidase activity | 6/222 | 68/18496 | 0.000164475 | 0.019449223 | 0.017443055 | CPXM2/NPEPPS/CPXM1/CPA3/ANPEP/CPZ | 6 |
| MF | GO:0015631 | tubulin binding | 14/222 | 379/18496 | 0.000205671 | 0.019456486 | 0.017449569 | KIF11/KIF18B/KIF2C/KIF24/KIF20A/KIF14/GJA1/KIFC1/RCC2/STMN2/BIRC5/CENPE/BRCA1/CLIP4 | 14 |
| MF | GO:0005044 | scavenger receptor activity | 4/222 | 27/18496 | 0.000285392 | 0.021190724 | 0.019004922 | CD36/SCARF2/COLEC12/STAB2 | 4 |
| MF | GO:0032036 | myosin heavy chain binding | 3/222 | 12/18496 | 0.000346446 | 0.021190724 | 0.019004922 | PDLIM2/CORO1A/MYL3 | 3 |
| MF | GO:0001968 | fibronectin binding | 4/222 | 29/18496 | 0.000379041 | 0.021190724 | 0.019004922 | IGFBP5/CCDC80/SDC4/MMP2 | 4 |
| MF | GO:0008237 | metallopeptidase activity | 9/222 | 185/18496 | 0.000407382 | 0.021190724 | 0.019004922 | CPXM2/NPEPPS/CPXM1/CPA3/ANPEP/ADAM33/ADAMTS8/MMP2/CPZ | 9 |
| MF | GO:0008017 | microtubule binding | 11/222 | 273/18496 | 0.000479421 | 0.021190724 | 0.019004922 | KIF11/KIF18B/KIF2C/KIF24/KIF20A/KIF14/KIFC1/RCC2/BIRC5/CENPE/CLIP4 | 11 |
| MF | GO:0004181 | metallocarboxypeptidase activity | 4/222 | 31/18496 | 0.000492808 | 0.021190724 | 0.019004922 | CPXM2/CPXM1/CPA3/CPZ | 4 |
| MF | GO:0008574 | plus-end-directed microtubule motor activity | 3/222 | 16/18496 | 0.000851126 | 0.033548552 | 0.030088052 | KIF11/KIF18B/KIF14 | 3 |
| MF | GO:0030169 | low-density lipoprotein particle binding | 3/222 | 17/18496 | 0.001024398 | 0.037272324 | 0.033427721 | CD36/COLEC12/STAB2 | 3 |
| MF | GO:0008238 | exopeptidase activity | 6/222 | 102/18496 | 0.001444198 | 0.046649335 | 0.041837503 | CPXM2/NPEPPS/CPXM1/CPA3/ANPEP/CPZ | 6 |
| MF | GO:0016887 | ATP hydrolysis activity | 13/222 | 412/18496 | 0.001479366 | 0.046649335 | 0.041837503 | SMC1B/KIF18B/CDC6/KIF2C/ERCC6L/KIF20A/KIF14/MCM7/BRIP1/KIFC1/RFC5/ABCA5/HSPA1B | 13 |

**Supplementary Table 5.** GSEA of BUB1B in the LSIL vs. HSIL group

| Description | Set Size | Enrichment Score | NES | *p*-value | *p*.adjust | *q*-value | Rank | Leading edge | Core enrichment |
| --- | --- | --- | --- | --- | --- | --- | --- | --- | --- |
| Linoleic acid metabolism | 21 | 0.60459805050553 | 2.01797989517157 | 0.00119450462831828 | 0.00119450462831828 | 0.0937361444487224 | 2528 | tags=38%, list=16%, signal=32% | CYP2C19/PLA2G4E/PLA2G2F/JMJD7-PLA2G4B/CYP2C8/PLA2G2D/AKR1B10/PLA2G4A |
| Cardiac muscle contraction | 61 | 0.421115093131915 | 1.79520921202339 | 0.0013175018896951 | 0.0013175018896951 | 0.0937361444487224 | 1642 | tags=26%, list=10%, signal=24% | UQCRQ/ATP1B1/TNNT2/CACNA2D4/COX5B/COX7A2/CACNA1D/MYL3/UQCRHL/COX7C/FXYD2/TPM3/ATP2A2/MT-CO2/TNNC1/COX6B2 |
| Ribosome | 85 | 0.366852948734168 | 1.67052753028573 | 0.00185933202266099 | 0.00185933202266099 | 0.0937361444487224 | 3188 | tags=36%, list=20%, signal=29% | RPL22/RPL13/RPS29/RPL37/RPL3L/RPL8/RPS6/RPL36A/RPL11/RPS15A/RPS9/RPL39/RPS15/RPL13A/RPL36/RPL26L1/RPL26/RPS27/RPL29/FAU/RPS20/RPL9/RPL17/RPL32/RPS27L/RPL18/RPS4X/RPS18/RPS2/RPL30/RPL34 |
| Oxidative phosphorylation | 123 | 0.328140624927685 | 1.5752000575194 | 0.00213291825691704 | 0.00213291825691704 | 0.0937361444487224 | 3318 | tags=27%, list=21%, signal=21% | NDUFB10/NDUFA3/UQCRQ/NDUFB4/ATP6V0D1/SDHB/COX5B/COX7A2/NDUFS4/ATP6V0C/NDUFA8/UQCRHL/COX7C/MT-ND6/NDUFV2/NDUFB8/MT-CO2/COX6B2/NDUFC2/ATP6V1E1/MT-ND4L/ATP6V1A/NDUFS5/ATP5ME/COX6C/MT-ND1/COX17/ATP5PB/NDUFB6/MT-CYB/UQCRB/ATP6V1B1/UQCRFS1 |
| Ether lipid metabolism | 28 | 0.521410593506631 | 1.85443447391992 | 0.00302988060655747 | 0.00302988060655747 | 0.106524223430547 | 2528 | tags=36%, list=16%, signal=30% | PLA2G4E/LPCAT1/PLA2G2F/JMJD7-PLA2G4B/PAFAH2/PLA2G2D/ENPP6/PLD2/PLA2G7/PLA2G4A |
| Parkinson's disease | 121 | 0.311622222980051 | 1.49042673836738 | 0.00483516644981716 | 0.00483516644981716 | 0.141483146149879 | 2916 | tags=27%, list=18%, signal=23% | NDUFB10/NDUFA3/UQCRQ/NDUFB4/UBE2J2/SDHB/COX5B/COX7A2/SLC6A3/UBE2L6/SLC25A6/NDUFS4/NDUFA8/UQCRHL/UCHL1/COX7C/MT-ND6/UBB/NDUFV2/NDUFB8/MT-CO2/COX6B2/NDUFC2/CYCS/MT-ND4L/UBE2G1/NDUFS5/COX6C/MT-ND1/ATP5PB/PARK7/NDUFB6/MT-CYB |
| Metabolism of xenobiotics by cytochrome P450 | 43 | 0.424548724630797 | 1.66934487531253 | 0.00563390971195626 | 0.00563390971195626 | 0.141483146149879 | 2571 | tags=35%, list=16%, signal=29% | CYP2C19/ALDH3B2/GSTM5/GSTM1/GSTM4/CYP2C8/ADH7/UGT1A6/GSTA1/MGST1/CYP2S1/ADH1C/GSTM3/AKR1C3/AKR1C1 |
| Complement and coagulation cascades | 58 | -0.388247843 | -1.562600538 | 0.00907916523267923 | 0.00907916523267923 | 0.199502709718083 | 3054 | tags=31%, list=19%, signal=25% | C4BPA/CFB/FGA/VWF/C1QA/C8G/F10/PLAUR/CD59/SERPINA1/A2M/C5/C4B/FGB/F8/F13A1/F2R/PROS1 |
| Drug metabolism - cytochrome P450 | 45 | 0.398480775935909 | 1.58517836434892 | 0.0132858932734329 | 0.0132858932734329 | 0.230771152865573 | 1070 | tags=24%, list=7%, signal=23% | CYP2C19/ALDH3B2/GSTM5/GSTM1/GSTM4/FMO3/CYP2C8/ADH7/UGT1A6/GSTA1/MGST1 |
| Other glycan degradation | 14 | -0.58152299 | -1.653179501 | 0.0137045668682978 | 0.0137045668682978 | 0.230771152865573 | 2599 | tags=50%, list=16%, signal=42% | FUCA1/ENGASE/MAN2C1/FUCA2/MANBA/MAN2B1/HEXA |
| Adherens junction | 72 | -0.359006527 | -1.495532127 | 0.0144404703439834 | 0.0144404703439834 | 0.230771152865573 | 2876 | tags=32%, list=18%, signal=26% | FER/INSR/TCF7/IGF1R/FGFR1/ACTG1/WASL/TJP1/WASF3/SNAI1/CREBBP/YES1/PTPN6/WASF1/MAPK1/FARP2/TGFBR2/ACTB/CTNNA3/NECTIN3/CTNNA1/NLK/PTPRM |
| alpha-Linolenic acid metabolism | 14 | 0.561772804343621 | 1.68967910465259 | 0.0251481498073639 | 0.0251481498073639 | 0.368398334897349 | 1250 | tags=29%, list=8%, signal=26% | PLA2G4E/PLA2G2F/JMJD7-PLA2G4B/PLA2G2D |
| Bladder cancer | 42 | 0.376185260965601 | 1.46826810986592 | 0.0430070449111969 | 0.0430070449111969 | 0.472247672695601 | 3680 | tags=43%, list=23%, signal=33% | MMP1/CXCL8/E2F2/ERBB2/BRAF/VEGFC/KRAS/TP53/MAP2K1/THBS1/DAPK2/RPS6KA5/FGFR3/E2F1/MAP2K2/CDKN2A/EGF/TYMP |
| Cell cycle | 122 | 0.272566912017618 | 1.31042093040267 | 0.044759812095337 | 0.044759812095337 | 0.472247672695601 | 3596 | tags=30%, list=22%, signal=23% | BUB1/CDC20/ANAPC11/CDC25C/CCNB2/E2F2/CCNB3/CDK1/BUB3/PCNA/GADD45A/ORC3/CCNB1/TP53/ANAPC10/CDKN2B/PTTG1/SKP1/ESPL1/YWHAQ/CCNE1/CCND2/YWHAZ/SKP2/ZBTB17/TTK/E2F1/CCNA1/CHEK1/SMAD4/SFN/ANAPC1/CDKN1C/CDKN2A/ANAPC4/YWHAG |
| Cytokine-cytokine receptor interaction | 221 | 0.235746267118579 | 1.24462532688837 | 0.0478005601996398 | 0.0478005601996398 | 0.472247672695601 | 3482 | tags=29%, list=22%, signal=23% | IL21R/TNFRSF9/TNF/TNFRSF14/CXCL10/TNFRSF10B/CTF1/CXCL8/CCL22/CXCL11/TNFSF4/IL22RA2/IL10/IL18RAP/CCL26/VEGFC/MPL/CX3CR1/FLT3/CCR7/CCL3/IFNGR1/CCL7/KIT/CCL18/IL12B/CXCR6/IFNAR1/TNFRSF21/CCL17/TNFRSF11A/CCL2/XCL2/CSF3R/TNFRSF13C/LTB/CCL4L2/PDGFA/CD40LG/CCR5/IL7R/IL11RA/INHBA/CCL21/CXCL2/TNFRSF6B/CXCL16/IL15/IL15RA/TNFRSF13B/IL13RA1/XCL1/TNFRSF17/CCL4/IFNE/CNTFR/OSM/CCL8/IL22RA1/IL10RB/IL12A/IL18/IL1RAP/EGF/IL6ST |
| p53 signaling pathway | 68 | 0.315546845825358 | 1.37885126209463 | 0.0481249521181575 | 0.0481249521181575 | 0.472247672695601 | 4047 | tags=38%, list=25%, signal=29% | CCNB2/TNFRSF10B/CCNB3/CDK1/GADD45A/TSC2/CCNB1/TP53/THBS1/CCNE1/CCND2/CYCS/IGFBP3/PMAIP1/GTSE1/RRM2B/CHEK1/PERP/SFN/COP1/CDKN2A/RRM2/TP53AIP1/CCNG1/EI24/APAF1 |
| Maturity onset diabetes of the young | 14 | -0.536794954 | -1.526024643 | 0.0485981308411215 | 0.0485981308411215 | 0.472247672695601 | 2561 | tags=50%, list=16%, signal=42% | MAFA/PAX6/FOXA3/BHLHA15/HNF1B/HNF4G/FOXA2 |
| N-Glycan biosynthesis | 46 | -0.378346561 | -1.443078718 | 0.0487804878048781 | 0.0487804878048781 | 0.472247672695601 | 4363 | tags=41%, list=27%, signal=30% | DPAGT1/DPM2/RPN1/MGAT5/MAN1A2/MAN2A1/ALG13/ALG3/MGAT4A/ALG12/ALG9/TUSC3/ST6GAL1/RFT1/ALG6/B4GALT1/MGAT3/MAN1A1/MGAT5B |

**Supplementary Table 6.** GSEA of BUB1B in the HSIL vs. SCC group

| Description | Set Size | Enrichment Score | NES | *p*-value | *p*.adjust | *q*-value | Rank | Leading edge | Core enrichment |
| --- | --- | --- | --- | --- | --- | --- | --- | --- | --- |
| Ribosome | 85 | 0.490298447449707 | 1.84642821531467 | 1.84223078004422e-05 | 1.84223078004422e-05 | 0.00327723159818393 | 6087 | tags=62%, list=38%, signal=39% | RPS16/RPL3/RPL5/RPS21/RPL31/RPSA/RPLP0/RPL12/RPLP1/RPS25/RPS18/RPS20/RPL10A/RPS24/RPL35/RPS8/RPL4/RPL18/RPL14/RPS3A/RPS28/RPS5/RPLP2/RPL11/RPL35A/RPL7/RPS13/RPL22L1/RSL24D1/RPL39/RPL13A/RPS23/RPS17/RPL37/RPL23A/RPS27A/RPL15/RPS11/MRPL13/RPL26/RPL27/RPL30/RPL13/RPS3/RPL9/RPS7/RPL27A/RPS26/UBA52/RPS10/RPS27/RPL10/RPL23 |
| Antigen processing and presentation | 63 | 0.47563865127548 | 1.72667162995629 | 0.00112837885031295 | 0.00112837885031295 | 0.10036632931731 | 4739 | tags=51%, list=29%, signal=36% | NFYC/RFXANK/HLA-F/NFYA/HLA-DMB/CD74/KIR3DL2/HLA-DQA1/HLA-DRA/HLA-DPA1/HLA-B/HSPA4/TAPBP/HLA-DPB1/PSME2/KIR2DL4/CANX/HLA-DQA2/CTSL/CIITA/HLA-DOA/CALR/CD8B/TAP1/HSP90AB1/HLA-DQB1/HSPA5/RFXAP/HSPA1B/HLA-C/LTA/HLA-DRB1 |
| Endometrial cancer | 51 | 0.463123597281703 | 1.63439035417304 | 0.0029159955070867 | 0.0029159955070867 | 0.17291341778865 | 3857 | tags=45%, list=24%, signal=34% | CTNNA1/BAD/TP53/AXIN2/AKT2/AKT1/PTEN/FOXO3/HRAS/PIK3R1/ELK1/PIK3CD/PIK3R3/PIK3R5/CTNNB1/SOS2/SOS1/EGFR/PIK3R2/MAPK3/ARAF/GRB2/ERBB2 |
| Lysosome | 118 | 0.379650986505591 | 1.48447562374545 | 0.00475043064190632 | 0.00475043064190632 | 0.211269152232149 | 3440 | tags=31%, list=21%, signal=25% | AP3M2/LAPTM5/HEXA/GALNS/FUCA1/SUMF1/ATP6V0B/TPP1/CTSD/CTSZ/GGA1/LAMP2/GUSB/MAN2B1/CLTA/AP3S1/SLC17A5/ATP6V0A1/CTNS/ACP5/LIPA/CLTCL1/ARSA/AP1G1/AP4B1/PPT1/AP3B1/CTSF/LAPTM4A/ARSG/CTSK/IGF2R/ABCA2/CTSL/HGSNAT/CTSG/CTSA |
| Renal cell carcinoma | 69 | 0.418312643259428 | 1.53639741360234 | 0.0091306450788748 | 0.0091306450788748 | 0.294650382549232 | 3855 | tags=39%, list=24%, signal=30% | PTPN11/PAK6/VEGFB/CDC42/VHL/PAK4/CREBBP/AKT2/AKT1/HRAS/PIK3R1/RBX1/PIK3CD/PIK3R3/PIK3R5/PAK1/PAK3/CRK/PDGFB/EGLN1/SOS2/JUN/SOS1/PIK3R2/MAPK3/ARAF/GRB2 |
| Chemokine signaling pathway | 175 | 0.346662817574995 | 1.40326028950822 | 0.00993791231083207 | 0.00993791231083207 | 0.294650382549232 | 3985 | tags=34%, list=25%, signal=26% | CX3CL1/CCL19/ADCY6/FGR/CDC42/PRKCZ/GRK3/GNG5/PLCB1/ADCY1/GRK4/STAT5B/GNG11/CXCL3/GRK2/PRKACA/PLCB2/AKT2/AKT1/GRK6/PLCB4/LYN/SHC4/GNG12/FOXO3/HRAS/PIK3R1/GNG7/GNB2/VAV3/PIK3CD/GNG8/PIK3R3/IKBKB/STAT3/CXCL9/PIK3R5/PRKACB/PAK1/CRK/ADCY4/CCL23/RELA/NFKBIA/SOS2/PRKCB/XCR1/SOS1/PIK3R2/GNB5/CCL5/CCR9/CXCR3/MAPK3/TIAM2/GRB2/PRKX/VAV1/CCL14 |
| Other glycan degradation | 14 | 0.627048545179607 | 1.70118250138527 | 0.0123171328098281 | 0.0123171328098281 | 0.31302187140766 | 5104 | tags=64%, list=32%, signal=44% | HEXA/FUCA1/ENGASE/FUCA2/MAN2B1/MAN2C1/MAN2B2/GLB1/AGA |
| Linoleic acid metabolism | 21 | -0.444202508 | -1.666014391 | 0.0152748599048248 | 0.0152748599048248 | 0.339170664614882 | 1048 | tags=24%, list=6%, signal=22% | PLA2G4E/CYP2J2/AKR1B10/CYP2C18/CYP2C19 |
| ErbB signaling pathway | 84 | 0.387142923916403 | 1.45695420502074 | 0.0171592259316996 | 0.0171592259316996 | 0.339170664614882 | 3857 | tags=40%, list=24%, signal=31% | PAK6/MAP2K4/RPS6KB2/NRG4/BAD/STAT5B/BTC/PAK4/AKT2/AKT1/MTOR/SHC4/HRAS/PIK3R1/ELK1/PIK3CD/PIK3R3/PIK3R5/PAK1/PAK3/CRK/SOS2/CAMK2D/PRKCB/JUN/SOS1/EGFR/PIK3R2/CBLC/MAPK3/CAMK2B/ARAF/GRB2/ERBB2 |
| Chronic myeloid leukemia | 73 | 0.380390173415923 | 1.41345854964145 | 0.0230409783204183 | 0.0230409783204183 | 0.409886877489547 | 3855 | tags=40%, list=24%, signal=30% | PTPN11/RB1/BAD/STAT5B/TP53/CTBP2/AKT2/AKT1/SHC4/GAB2/HRAS/PIK3R1/PIK3CD/PIK3R3/IKBKB/PIK3R5/CRK/RELA/NFKBIA/SOS2/SMAD3/SOS1/PIK3R2/CBLC/TGFBR2/SMAD4/MAPK3/ARAF/GRB2 |
| Non-small cell lung cancer | 53 | 0.417660597946405 | 1.4786637740547 | 0.0253450910238318 | 0.0253450910238318 | 0.409887117993069 | 4074 | tags=45%, list=25%, signal=34% | RB1/STK4/BAD/TP53/AKT2/AKT1/FOXO3/HRAS/PIK3R1/PIK3CD/PIK3R3/PIK3R5/SOS2/RASSF5/RXRG/PRKCB/SOS1/EGFR/PIK3R2/MAPK3/ARAF/GRB2/ERBB2/RXRA |
| Prostate cancer | 87 | 0.372787459817668 | 1.41042217501527 | 0.0288625243009861 | 0.0288625243009861 | 0.427874263760232 | 3901 | tags=39%, list=24%, signal=30% | NKX3-1/RB1/IGF1R/BAD/TP53/CREBBP/AKT2/AKT1/PTEN/MTOR/HRAS/PIK3R1/BCL2/PIK3CD/PIK3R3/IKBKB/CREB3L4/PDGFRA/PIK3R5/FGFR1/CTNNB1/PDGFB/RELA/NFKBIA/SOS2/SOS1/EGFR/PIK3R2/ATF4/MAPK3/ARAF/GRB2/ERBB2/HSP90AB1 |
| Drug metabolism - cytochrome P450 | 45 | -0.305881218 | -1.385822619 | 0.0423515788833697 | 0.0423515788833697 | 0.498730789911343 | 1732 | tags=24%, list=11%, signal=22% | GSTM5/FMO2/UGT2B7/GSTA1/GSTA4/UGT1A6/ALDH3A1/GSTM3/GSTM1/CYP2C18/CYP2C19 |
| Adherens junction | 72 | 0.363795203077812 | 1.34831058975975 | 0.0464088397790055 | 0.0464088397790055 | 0.498730789911343 | 5500 | tags=44%, list=34%, signal=29% | FARP2/IGF1R/WASF3/CDC42/CTNNA1/CSNK2A1/YES1/CREBBP/ACTB/ACP1/PTPRM/VCL/ACTN2/TJP1/FGFR1/CTNNB1/INSR/SMAD3/EGFR/TGFBR2/SMAD4/MAPK3/ERBB2/ACTG1/IQGAP1/SMAD2/LEF1/PTPN1/PTPN6/RAC2/LMO7/FER |
| Wnt signaling pathway | 142 | 0.318407238344692 | 1.27255336815231 | 0.047569803516029 | 0.047569803516029 | 0.498730789911343 | 6080 | tags=49%, list=38%, signal=31% | PRICKLE2/PRICKLE1/VANGL1/LRP6/PLCB1/CSNK2A1/FBXW11/DVL2/PRKACA/TP53/SOX17/FRAT2/WNT16/CHD8/CTBP2/FZD4/CREBBP/AXIN2/PLCB2/SFRP2/SIAH1/PLCB4/WNT7B/FRAT1/RBX1/NKD2/PRKACB/NFAT5/CTNNB1/DAAM1/CAMK2D/PRKCB/SMAD3/JUN/PPP3CA/FZD8/CXXC4/SMAD4/CAMK2B/PRKX/FZD2/RUVBL1/FZD9/SFRP1/MMP7/ROCK2/SMAD2/VANGL2/PPP2CB/DAAM2/CTNNBIP1/TBL1XR1/LEF1/LRP5/CAMK2G/WNT5B/RAC2/FZD5/TBL1X/BTRC/NFATC2/NFATC4/AXIN1/CUL1/WNT11/CAMK2A/MAPK9/WNT5A/CSNK2A2 |
| Metabolism of xenobiotics by cytochrome P450 | 43 | -0.30858018 | -1.37541297 | 0.0490372158432449 | 0.0490372158432449 | 0.498730789911343 | 1670 | tags=23%, list=10%, signal=21% | AKR1C2/UGT2B7/GSTA1/GSTA4/UGT1A6/ALDH3A1/GSTM3/GSTM1/CYP2C18/CYP2C19 |

**Supplementary Table 7.** GSEA of KIF14 in the LSIL vs. HSIL group

| Description | Set Size | Enrichment Score | NES | *p*-value | *p*.adjust | *q*-value | Rank | Leading edge | Core enrichment |
| --- | --- | --- | --- | --- | --- | --- | --- | --- | --- |
| Cytokine-cytokine receptor interaction | 221 | 0.277004941125239 | 1.45996455676598 | 0.00348689515246339 | 0.00348689515246339 | 0.277764686110563 | 3692 | tags=33%, list=23%, signal=26% | TNFRSF9/CXCR6/TNFRSF10B/CCL22/CNTFR/IFNGR1/LTB/TNFRSF13B/CCR7/IL21R/MPL/TNF/CCL26/IL6ST/CXCL10/IL18RAP/TNFRSF13C/FLT3/TNFRSF25/IFNE/IL11RA/TNFSF4/CSF2RB/TNFRSF14/CX3CR1/IL12B/CCL17/TNFRSF1A/PF4V1/XCL2/CSF2RA/CTF1/IL20RB/CXCR4/TNFRSF1B/IL17RB/TNFRSF21/IL10/TNFRSF11A/CCR5/IL1A/LTA/CCL21/IFNAR1/IFNAR2/CCL8/IL26/IL22RA1/TGFB3/EGF/CD40/CCL2/XCR1/PDGFC/CSF3R/IL6R/LIF/CCR9/BMP2/LEPR/CXCL16/XCL1/CCL7/CXCL9/CCL18/HGF/BMPR2/TNFSF12/TNFRSF17/CCR4/TNFSF13B/EGFR/CXCL11 |
| Adipocytokine signaling pathway | 59 | 0.382920044082773 | 1.60126481714706 | 0.0048403661429183 | 0.0048403661429183 | 0.277764686110563 | 3286 | tags=36%, list=20%, signal=28% | CD36/ACSL4/MAPK10/TNF/CPT1A/IRS1/NFKBIA/MAPK9/PRKAA2/TNFRSF1A/RXRB/ACSL6/TNFRSF1B/IRS4/AKT3/PRKCQ/CHUK/IKBKG/LEPR/NFKB1/PCK2 |
| Regulation of actin cytoskeleton | 192 | 0.281656560059529 | 1.45949336333625 | 0.00537901062630049 | 0.00537901062630049 | 0.277764686110563 | 4829 | tags=43%, list=30%, signal=31% | FGFR2/MYLK3/FGFR3/SOS2/MYL12A/PIP5K1A/MYL7/DIAPH3/ARHGAP35/RAC3/FGF7/GNA13/ITGB7/NCKAP1/KRAS/CYFIP2/PAK6/FN1/PFN2/VAV2/CFL1/ARPC5L/RDX/PIP4K2B/MYH10/MAP2K1/FGF11/GSN/ITGA7/SLC9A1/ITGAL/PTK2/SSH2/APC/FGF2/MYH9/MYLK/SSH3/ROCK1/RAC1/PPP1R12A/RRAS/ARPC4/ITGAM/ITGB3/ARPC1A/PIK3CA/ITGB2/ITGB1/EGF/VAV1/GNG12/SCIN/PDGFC/FGF22/ABI2/ITGA5/FGF9/MYL5/BCAR1/EGFR/MYL12B/ARPC5/VCL/FGFR4/CYFIP1/BRAF/INSRR/IQGAP3/PDGFRA/ITGAV/ITGB6/SOS1/PDGFB/MSN/ARHGEF7/CSK/IQGAP1/ARHGEF4/ITGA2/CRKL/ITGA3/FGF1 |
| Toll-like receptor signaling pathway | 86 | 0.339641740983646 | 1.53378729303104 | 0.00617254858023473 | 0.00617254858023473 | 0.277764686110563 | 3354 | tags=31%, list=21%, signal=25% | TLR7/MAPK10/TNF/CD86/CXCL10/SPP1/NFKBIA/MAPK9/IL12B/TICAM2/MAP2K1/TAB2/TLR2/AKT3/RAC1/TIRAP/IFNAR1/IFNAR2/IKBKE/PIK3CA/TLR4/CD40/CHUK/IKBKG/NFKB1/CXCL9/MAPK11 |
| RIG-I-like receptor signaling pathway | 53 | 0.384927587408339 | 1.55302500787788 | 0.00811289567229561 | 0.00811289567229561 | 0.292064244202642 | 4439 | tags=45%, list=27%, signal=33% | CASP10/MAPK10/TANK/TNF/CXCL10/NLRX1/IFNE/NFKBIA/MAPK9/IL12B/SIKE1/TBKBP1/IKBKE/DDX3X/RNF125/CHUK/IKBKG/NFKB1/MAPK11/PIN1/OTUD5/TRAF2/ATG5/ATG12 |
| T cell receptor signaling pathway | 102 | 0.309276964219783 | 1.43781315028275 | 0.0127351397358169 | 0.0127351397358169 | 0.360662025267282 | 3423 | tags=30%, list=21%, signal=24% | SOS2/NCK2/TNF/NFAT5/CBLB/KRAS/NFKBIA/PAK6/TEC/CTLA4/MAPK9/VAV2/MAP2K1/NFATC2/GRAP2/IL10/AKT3/CBLC/PRKCQ/FYN/PIK3CA/CD247/RASGRP1/VAV1/CD8A/ITK/CHUK/IKBKG/NFKB1/MAPK11/CD4 |
| Phenylalanine metabolism | 17 | 0.542483537437062 | 1.63917151784395 | 0.0155445228003707 | 0.0155445228003707 | 0.360662025267282 | 2282 | tags=35%, list=14%, signal=30% | ALDH3B2/NAA80/MIF/PRDX6/HPD/ALDH1A3 |
| Ribosome | 85 | -0.346489318 | -1.479714863 | 0.0160294233452125 | 0.0160294233452125 | 0.360662025267282 | 2829 | tags=29%, list=17%, signal=24% | RPS5/RPL4/RPL35A/RPS21/RPL38/RPL35/RPL10A/RPL18A/RPL14/RPL30/RPS13/RPS3A/RPS23/RPS17/RPS28/RPLP1/RPLP2/RPSA/RPL10/UBA52/RPL5/RPL27A/RPL12/RPS16/RPL36AL |
| Chemokine signaling pathway | 175 | 0.263461603248635 | 1.34964276089189 | 0.0247910070532679 | 0.0247910070532679 | 0.495820141065359 | 3397 | tags=30%, list=21%, signal=24% | CXCR6/CCL22/SOS2/DOCK2/CCR7/CCL26/HCK/CXCL10/FOXO3/KRAS/NFKBIA/GNG10/ADCY7/VAV2/GNGT2/CX3CR1/RASGRP2/CCL17/PF4V1/GNB4/MAP2K1/XCL2/PREX1/ADCY9/CXCR4/STAT2/PTK2/GRK7/CCR5/AKT3/ROCK1/RAC1/ARRB1/CCL21/CCL8/PIK3CA/VAV1/CCL2/XCR1/GNG12/ITK/CCR9/CHUK/PTK2B/IKBKG/NFKB1/CXCL16/PLCB4/XCL1/CCL7/CXCL9/CCL18 |
| MAPK signaling pathway | 241 | 0.248289428534837 | 1.31965985100859 | 0.0296841366358476 | 0.0296841366358476 | 0.497094631482891 | 3168 | tags=24%, list=20%, signal=20% | MAP3K12/FGFR2/NGF/FGFR3/SOS2/RASGRP4/RPS6KA5/TP53/PLA2G4A/MAPK10/STMN1/TNF/PPM1B/DAXX/DUSP1/RAC3/FGF7/MAPK8IP2/KRAS/HSPA1L/RPS6KA4/CACNA1I/MAP3K3/MAPK9/FLNB/CACNA1G/RASGRP2/PLA2G4E/CACNA1A/TNFRSF1A/CDC25B/MAP2K1/FGF11/NFATC2/TAB2/FGF2/IL1A/AKT3/RAC1/ARRB1/CACNA2D2/RRAS/MAP3K5/MAPK8IP3/RPS6KA2/PTPN7/MAP4K4/TGFB3/RASGRP1/STK4/EGF/GNG12/CHUK/GADD45A/IKBKG/FGF22/DUSP10/NFKB1 |
| Mismatch repair | 22 | 0.46259094599448 | 1.50889314915263 | 0.0303780052572878 | 0.0303780052572878 | 0.497094631482891 | 1277 | tags=27%, list=8%, signal=25% | PCNA/RFC2/POLD1/RPA1/MLH3/EXO1 |
| Valine, leucine and isoleucine biosynthesis | 10 | 0.593986114817071 | 1.54301479843509 | 0.0368529995891428 | 0.0368529995891428 | 0.535162337502786 | 1830 | tags=40%, list=11%, signal=35% | BCAT1/VARS1/IARS2/PDHA1 |
| Bladder cancer | 42 | 0.380979668752799 | 1.47117512084899 | 0.0386506132640901 | 0.0386506132640901 | 0.535162337502786 | 2771 | tags=33%, list=17%, signal=28% | E2F2/FGFR3/RPS6KA5/TP53/ERBB2/DAPK2/THBS1/KRAS/E2F1/MAP2K1/MMP1/CDH1/RB1/EGF |
| Neuroactive ligand-receptor interaction | 175 | -0.273123974 | -1.303733391 | 0.0488549618320611 | 0.0488549618320611 | 0.628135223555071 | 3401 | tags=27%, list=21%, signal=21% | CHRNA10/GRIA1/MTNR1A/LPAR3/AGTR1/OPRL1/CHRNA3/PTGER4/S1PR2/GPR83/S1PR3/P2RY10/MCHR1/TACR2/NPY1R/OXTR/GALR3/GABRR2/S1PR1/GRIN1/GRIK2/LHB/SSTR2/ADRA2A/VIPR2/GRM4/GABRB3/TRPV1/GRIA4/TSHR/GRID2/PTGER2/CHRM4/CRHR2/AVPR1A/GRM2/S1PR5/GRM7/ADCYAP1R1/CHRM5/RXFP1/GRIN2A/GABRD/GABRG3/GPR35/CHRNB2/F2R |

**Supplementary Table 8.** GSEA of KIF14 in the HSIL vs. SCC group

| Description | Set Size | Enrichment Score | NES | *p*-value | *p*.adjust | *q*-value | Rank | Leading edge | Core enrichment |
| --- | --- | --- | --- | --- | --- | --- | --- | --- | --- |
| Ribosome | 85 | 0.464126358464878 | 2.00996549960186 | 9.79096720484997e-06 | 9.79096720484997e-06 | 0.00157686103404426 | 3615 | tags=41%, list=22%, signal=32% | RPS27/RPL38/RPL30/RPS17/RPL36AL/RPS16/RPS21/RPL27A/RPL35/RPLP2/RPS28/RPL26L1/RPL18A/RPL22L1/RPS9/RPS18/RPL14/RPL35A/RPS25/RPL10/RPS23/RPL15/RPL10A/FAU/RPS24/RPL31/RPL26/RPS27L/RPL9/UBA52/RPS20/RPL37A/RPLP1/RPL8/RPL29 |
| Cytokine-cytokine receptor interaction | 221 | -0.302646556 | -1.593469585 | 0.00021319414473663 | 0.00021319414473663 | 0.0159238282059283 | 1994 | tags=44%, list=12%, signal=39% | CCR5/XCL1/IL20RB/TNFRSF8/TNFSF14/PDGFB/CCR4/TNFSF12/CCL19/CSF2RA/IL12RB1/EPO/CXCL16/CCL8/BMPR2/CSF3R/TGFB2/PF4V1/AMHR2/CCL22/OSMR/TGFB1/CCL21/CD70/OSM/TNFRSF21/TNFRSF13B/FLT1/TNFRSF9/CCL24/VEGFA/IL12B/IFNAR1/CRLF2/IFNE/IFNAR2/KDR/TNFSF13B/HGF/CXCL12/CCL17/CXCL6/IFNG/LTBR/CXCL8/TNFRSF25/BMP2/EGF/IL23A/TNFSF8/MET/CXCL10/EGFR/TNFRSF1B/IL2RG/CCL26/CSF3/CXCL11/IL6ST/IL10/CCR3/IL6R/CCR7/TGFBR2/IL21R/TNFRSF11A/TNFRSF10A/TNF/CD40/TNFSF11/PDGFC/CTF1/TNFRSF12A/IL17A/IL9R/IL2RB/IL26/TNFRSF13C/XCR1/CSF2RB/LIF/ACVR1B/CCL7/FLT3LG/LEPR/TGFBR1/TNFRSF10B/LTB/TGFB3/CXCL9/IL10RA/CCL2/CXCR6/CXCL2/FASLG/IL22RA1/CXCR4/XCL2 |
| T cell receptor signaling pathway | 102 | -0.374641148 | -1.77175476 | 0.000296620329326115 | 0.000296620329326115 | 0.0159238282059283 | 4575 | tags=50%, list=28%, signal=36% | PTPRC/ITK/PRKCQ/MAPK3/CTLA4/CD8B/MAP2K1/CHUK/PIK3R2/NFATC3/CDC42/LAT/IKBKB/RHOA/NFAT5/MAPK9/ICOS/PPP3CA/LCP2/PIK3CB/MALT1/PAK3/IFNG/PIK3CG/NFATC4/HRAS/BCL10/LCK/MAPK14/TEC/NFKBIA/AKT1/PDPK1/GRAP2/IL10/TNF/MAP3K8/CD3E/PAK1/PPP3CC/SOS2/PIK3R5/VAV2/VAV1/NFKB1/CD3G/VAV3/KRAS/CBLC/CD247/PPP3CB |
| Natural killer cell mediated cytotoxicity | 110 | -0.355637043 | -1.700692548 | 0.000712389830825343 | 0.000712389830825343 | 0.0286830642411257 | 3597 | tags=41%, list=22%, signal=32% | LAT/IFNAR1/GZMB/NFAT5/PPP3CA/IFNAR2/LCP2/HCST/PIK3CB/IFNG/PIK3CG/NFATC4/HLA-B/RAC1/HRAS/LCK/KLRC2/HLA-A/ULBP2/TNFRSF10A/FCGR3A/TNF/PAK1/PPP3CC/NCR1/SOS2/KLRC1/PIK3R5/SHC4/VAV2/VAV1/MICB/VAV3/KRAS/TNFRSF10B/ITGAL/CD244/MICA/CD247/PPP3CB/KLRD1/FASLG/KLRC3/PTK2B/KLRK1 |
| MAPK signaling pathway | 241 | -0.273600418 | -1.454589782 | 0.00132507560184285 | 0.00132507560184285 | 0.0426813825435696 | 4535 | tags=39%, list=28%, signal=28% | MEF2C/MAPK3/HSPA1A/MAP2K1/TAB1/TGFB2/MAP4K1/FGF1/HSPB1/NTF3/TAB2/CHUK/TGFB1/CACNA1E/DUSP7/MAPKAPK2/JMJD7-PLA2G4B/CDC42/RRAS/CACNG4/CRKL/CACNA1A/CACNB4/FGFR1/RPS6KA2/IKBKB/TRAF2/RAP1B/MAPK9/PPP3CA/MAP3K3/MAPK8/RPS6KA4/STMN1/CACNA1B/DUSP10/RPS6KA5/NFATC4/RAC1/HRAS/CDC25B/MAPK14/FLNB/MAP3K6/AKT1/DAXX/FGF2/MAPK8IP1/EGF/NR4A1/EGFR/NGF/DUSP3/MECOM/CACNA2D4/FGFR3/TGFBR2/HSPA1L/LAMTOR3/TNF/PRKACB/HSPA8/MAP3K8/PAK1/PPP3CC/SOS2/PTPN7/PPM1B/NTRK2/ATF4/ECSIT/MAPK8IP2/CACNA1I/ARRB2/CACNA2D2/MAPK10/PLA2G2D/PLA2G3/GADD45A/NFKB1/KRAS/TGFBR1/PTPN5/TGFB3/MAP3K4/MAPK8IP3/MKNK2/MAP3K12/PPP3CB/FASLG/DUSP1/FGFR2/CACNA1G |
| Type II diabetes mellitus | 42 | -0.447366968 | -1.741255401 | 0.00254660881921483 | 0.00254660881921483 | 0.0683563419894508 | 4905 | tags=60%, list=30%, signal=42% | SLC2A4/PIK3CA/MAPK3/IRS1/CACNA1E/PIK3R2/PRKCZ/PRKCE/CACNA1A/IKBKB/MAPK9/ABCC8/PIK3CB/MAPK8/CACNA1B/HK3/IRS2/PIK3CG/GCK/TNF/PIK3R5/SOCS1/IRS4/MAPK10/CACNA1G |
| Adipocytokine signaling pathway | 59 | -0.382079304 | -1.613141941 | 0.00532116919141365 | 0.00532116919141365 | 0.118268568075991 | 4655 | tags=47%, list=29%, signal=34% | ACSL3/PRKCQ/IRS1/PCK2/PRKAB1/CHUK/IKBKB/TRAF2/MAPK9/ADIPOR1/MAPK8/CPT1A/IRS2/POMC/NFKBIA/AKT1/RXRG/TNFRSF1B/PRKAA2/CAMKK1/TNF/PRKAG2/IRS4/RXRB/MAPK10/NFKB1/LEPR/ACSL6 |
| Pancreatic cancer | 70 | -0.366885629 | -1.601723545 | 0.00648327980769268 | 0.00648327980769268 | 0.118268568075991 | 4515 | tags=44%, list=28%, signal=32% | MAPK3/MAP2K1/TGFB2/CHUK/TGFB1/PIK3R2/CDC42/PGF/VEGFA/IKBKB/MAPK9/PIK3CB/MAPK8/RB1/PIK3CG/RAC1/AKT1/EGF/EGFR/TGFBR2/BAD/SMAD4/JAK1/PIK3R5/CDK6/MAPK10/E2F1/NFKB1/KRAS/TGFBR1/TGFB3 |
| Chemokine signaling pathway | 175 | -0.279338038 | -1.43241423 | 0.0066200499342427 | 0.0066200499342427 | 0.118268568075991 | 4767 | tags=42%, list=29%, signal=30% | GNGT2/CCR4/CCL19/PIK3CA/CXCL16/ITK/MAPK3/CCL8/MAP2K1/PF4V1/CCL22/CHUK/ROCK2/CCL21/PIK3R2/PRKCZ/STAT5B/PLCB3/CDC42/CRKL/CCL24/IKBKB/WAS/RAP1B/RHOA/FGR/PIK3CB/PREX1/CXCL12/CCL17/GNAI1/CXCL6/PIK3CG/RAC1/CXCL8/HRAS/NFKBIA/AKT1/ADCY4/CXCL10/CCL26/CXCL11/CSK/CCR3/GNG2/CCR7/FOXO3/PRKACB/PAK1/GNB3/ADCY3/SOS2/DOCK2/PIK3R5/PLCB2/SHC4/VAV2/ARRB2/XCR1/VAV1/CCL7/NFKB1/VAV3/KRAS/CXCL9/CCL2/CXCR6/CXCL2/HCK/CXCR4/ADCY7/PTK2B/XCL2 |
| TGF-beta signaling pathway | 81 | -0.349259401 | -1.5684195 | 0.00743342258136567 | 0.00743342258136567 | 0.118268568075991 | 4882 | tags=48%, list=30%, signal=34% | SMURF2/FST/PPP2CA/EP300/MAPK3/BMPR2/TGFB2/TFDP1/COMP/AMHR2/TGFB1/ROCK2/RBX1/CUL1/RHOA/ACVR1C/IFNG/ID4/ZFYVE16/THBS4/PPP2R1B/BMP2/LEFTY2/CHRD/GDF7/TGFBR2/SMAD9/SMAD4/ID3/E2F5/TNF/SP1/PPP2CB/TGFBR1/TGFB3/RPS6KB1/BMP8B/RBL2/THBS1 |
| Neurotrophin signaling pathway | 126 | -0.304959637 | -1.481354107 | 0.00807782049930787 | 0.00807782049930787 | 0.118268568075991 | 4696 | tags=44%, list=29%, signal=32% | PIK3CA/IRAK3/MAP2K5/MAPK3/MAP2K1/YWHAE/IRS1/BCL2/NTF3/NTRK3/MAPKAPK2/PIK3R2/CDC42/CRKL/RPS6KA2/IKBKB/RAP1B/RHOA/MAPK9/MAP3K3/PIK3CB/MAPK8/RPS6KA4/IRS2/RPS6KA5/PIK3CG/RAC1/HRAS/YWHAQ/RIPK2/IRAK1/GAB1/MAPK14/ARHGDIB/CALM3/NFKBIA/AKT1/ARHGDIA/PDPK1/NGF/CSK/FOXO3/SH2B3/BAD/IRAK2/SOS2/PIK3R5/NTRK2/ATF4/SHC4/IRS4/MAPK10/NFKB1/KRAS/FASLG/RAPGEF1 |
| Insulin signaling pathway | 126 | -0.297722879 | -1.446201257 | 0.0118680559020671 | 0.0118680559020671 | 0.12887854951611 | 4602 | tags=40%, list=28%, signal=29% | FLOT1/MAPK3/RHOQ/MAP2K1/IRS1/PCK2/PRKAB1/LIPE/FLOT2/PIK3R2/PRKCZ/PRKAR2B/RPS6/CRKL/IKBKB/MAPK9/PIK3CB/MAPK8/EIF4E/HK3/IRS2/PPP1R3D/PIK3CG/HRAS/CALM3/AKT1/GCK/PDPK1/PTPRF/PPP1R3B/PRKAA2/RHEB/BAD/PRKACB/PRKAG2/SREBF1/SOS2/PIK3R5/SOCS1/SHC4/IRS4/MAPK10/KRAS/GYS1/PHKA1/PYGM/CBLC/RPS6KB1/MKNK2/RAPGEF1 |
| Focal adhesion | 190 | -0.258802186 | -1.335185884 | 0.0127907429967175 | 0.0127907429967175 | 0.12887854951611 | 4696 | tags=40%, list=29%, signal=29% | PIK3CA/CAPN2/XIAP/CAV1/MAPK3/ITGA10/MAP2K1/COMP/BCL2/VWF/CCND2/ITGA5/DOCK1/ROCK2/PIK3R2/ACTN4/FLT1/ITGA1/CDC42/PGF/CRKL/VEGFA/RAP1B/RHOA/MAPK9/ITGB1/PIK3CB/MAPK8/PAK3/KDR/ZYX/HGF/IGF1/PIK3CG/PARVG/COL5A2/VCL/RAC1/HRAS/MYL12A/THBS4/COL11A2/COL6A1/FLNB/CHAD/TLN1/AKT1/COL5A3/TNC/EGF/MET/EGFR/PDPK1/ACTN1/BAD/PAK1/SPP1/PDGFC/SOS2/PDGFD/PIK3R5/CAV2/MYL7/SHC4/ITGB4/VAV2/MYLK3/MAPK10/TNN/VAV1/ITGA7/VAV3/RAPGEF1/MYL5/ITGB7/THBS1 |
| Alzheimer's disease | 157 | 0.286151494947427 | 1.38713197851496 | 0.0127922760686935 | 0.0127922760686935 | 0.12887854951611 | 2262 | tags=23%, list=14%, signal=20% | GNAQ/MAPT/COX5A/CASP7/APH1A/CDK5R1/FADD/ATP5F1C/NDUFS5/NDUFA4/UQCRHL/NDUFA6/LPL/NDUFV2/ADAM17/PLCB1/ATP5PF/CDK5/SDHC/PSENEN/MT-CYB/NDUFA3/COX7B/ATP5PD/CHP2/NDUFA10/ATP5MC3/COX4I1/COX6C/FAS/COX7A2L/GRIN2A/ATP2A1/UQCRFS1/UQCRH/CACNA1C |
| Toll-like receptor signaling pathway | 86 | -0.316718285 | -1.461655437 | 0.0129193858727864 | 0.0129193858727864 | 0.12887854951611 | 4515 | tags=44%, list=28%, signal=32% | MAPK3/IRF7/MAP2K1/TAB1/TAB2/CHUK/PIK3R2/IL12B/IFNAR1/IKBKB/TICAM1/MAPK9/IFNAR2/PIK3CB/MAPK8/PIK3CG/RAC1/CXCL8/IRAK1/TLR4/MAPK14/NFKBIA/AKT1/CD80/CXCL10/CXCL11/TNF/CD40/MAP3K8/SPP1/IKBKE/PIK3R5/MAPK10/TLR8/TLR2/NFKB1/CXCL9/TLR7 |
| Melanoma | 62 | -0.355792868 | -1.503153075 | 0.0131099237667794 | 0.0131099237667794 | 0.12887854951611 | 4975 | tags=48%, list=31%, signal=34% | CDKN1A/PDGFB/FGF10/PIK3CA/FGF7/MAPK3/MAP2K1/FGF1/PIK3R2/CDH1/FGFR1/PIK3CB/HGF/RB1/IGF1/PIK3CG/HRAS/MDM2/AKT1/FGF2/EGF/MET/EGFR/BAD/PDGFC/PDGFD/PIK3R5/CDK6/E2F1/KRAS |
| RIG-I-like receptor signaling pathway | 53 | -0.373977152 | -1.546549342 | 0.0136038468933672 | 0.0136038468933672 | 0.12887854951611 | 3535 | tags=40%, list=22%, signal=31% | IL12B/IKBKB/TRAF2/MAPK9/TBKBP1/IFNE/STING1/MAPK8/TRIM25/CXCL8/RNF125/MAPK14/NFKBIA/CXCL10/TNF/IKBKE/MAPK10/NFKB1/PIN1/TANK/CASP10 |
| Calcium signaling pathway | 149 | -0.278158705 | -1.39617732 | 0.015803641592191 | 0.015803641592191 | 0.141401003719604 | 2284 | tags=24%, list=14%, signal=21% | CALM3/GRM1/DRD1/GNAS/P2RX5/PDE1C/ADCY4/EGFR/NOS1/GNAL/ITPKA/ITPKB/PTGER1/ADORA2A/P2RX1/PRKACB/OXTR/TACR1/TRPC1/PPP3CC/ADCY3/CD38/NOS3/PLCB2/MYLK3/CACNA1I/EDNRB/ATP2B4/NOS2/PHKA1/HTR7/PPP3CB/SLC25A5/ADCY7/PTK2B/CACNA1G |
| Prostate cancer | 87 | -0.319905263 | -1.474573693 | 0.0177353269998445 | 0.0177353269998445 | 0.150332688696743 | 4806 | tags=44%, list=30%, signal=31% | PDGFB/AR/PIK3CA/EP300/MAPK3/CDKN1B/MAP2K1/BCL2/CHUK/PIK3R2/CREB1/FGFR1/IKBKB/PIK3CB/RB1/IGF1/PIK3CG/HRAS/MDM2/NFKBIA/AKT1/EGF/EGFR/PDPK1/CREB5/CDK2/BAD/SRD5A2/PDGFC/SOS2/PDGFD/KLK3/PIK3R5/ATF4/E2F1/NFKB1/KRAS/FGFR2 |
| Chronic myeloid leukemia | 73 | -0.332803783 | -1.467135846 | 0.0216949026456891 | 0.0216949026456891 | 0.174701058146865 | 4515 | tags=42%, list=28%, signal=31% | MAPK3/CDKN1B/MAP2K1/TGFB2/CHUK/TGFB1/PIK3R2/STAT5B/CRKL/IKBKB/PIK3CB/RB1/PIK3CG/HRAS/MDM2/NFKBIA/AKT1/MECOM/TGFBR2/BAD/SMAD4/SOS2/PIK3R5/SHC4/CDK6/E2F1/NFKB1/KRAS/TGFBR1/TGFB3/CBLC |
| Peroxisome | 75 | -0.317741801 | -1.410166062 | 0.024302634440054 | 0.024302634440054 | 0.178091146189494 | 3158 | tags=29%, list=20%, signal=24% | XDH/CROT/SLC27A2/DECR2/ABCD4/PEX11G/FAR2/HSD17B4/ABCD2/PEX16/PEX11B/AMACR/PAOX/PEX1/MVK/PEX7/NOS2/EPHX2/PEX14/ACSL6/PEX12/DDO |
| Amino sugar and nucleotide sugar metabolism | 43 | -0.375372042 | -1.468437584 | 0.0250481983987404 | 0.0250481983987404 | 0.178091146189494 | 4876 | tags=42%, list=30%, signal=29% | GMDS/GALE/UAP1/NANP/FPGT/PMM1/GNPNAT1/CMAS/HK3/NPL/AMDHD2/GCK/GNE/MPI/GALK1/PMM2/UGDH/CHIT1 |
| Intestinal immune network for IgA production | 41 | -0.379793723 | -1.466014021 | 0.025433278066931 | 0.025433278066931 | 0.178091146189494 | 3839 | tags=51%, list=24%, signal=39% | HLA-DPB1/IL15/MAP3K14/HLA-DRA/HLA-DOA/TGFB1/TNFRSF13B/MADCAM1/ICOS/TNFSF13B/CXCL12/HLA-DQA2/LTBR/CD80/ICOSLG/IL10/CD40/HLA-DMA/TNFRSF13C/CXCR4/ITGB7 |
| Aminoacyl-tRNA biosynthesis | 41 | -0.371400193 | -1.433614771 | 0.0314175016943858 | 0.0314175016943858 | 0.210827971896536 | 3134 | tags=32%, list=19%, signal=26% | YARS1/RARS1/NARS1/RARS2/GARS1/DARS2/YARS2/CARS2/NARS2/VARS1/SEPSECS/WARS1/WARS2 |
| Steroid biosynthesis | 17 | -0.497104018 | -1.558879054 | 0.0336698811170448 | 0.0336698811170448 | 0.216904918354015 | 4805 | tags=59%, list=30%, signal=41% | SOAT1/FDFT1/CYP51A1/MSMO1/HSD17B7/DHCR7/LIPA/NSDHL/LSS/CYP27B1 |
| Regulation of actin cytoskeleton | 192 | -0.243045105 | -1.251124257 | 0.0382946612764548 | 0.0382946612764548 | 0.237209845153748 | 4181 | tags=43%, list=26%, signal=32% | PPP1CB/ARHGAP35/GSN/RAC3/FGD1/PDGFB/FGF10/PIK3CA/FGF7/APC/MAPK3/ABI2/ITGA10/MAP2K1/FGF1/ITGA5/DOCK1/LIMK1/ROCK2/TMSB4X/PIK3R2/ACTN4/NCKAP1L/DIAPH2/ITGA1/CDC42/RRAS/CRKL/ARHGEF4/FGFR1/WAS/PIP5K1B/RHOA/ITGB1/PIK3CB/ITGAX/PAK3/WASF1/ARHGEF7/PIK3CG/VCL/RAC1/HRAS/MYL12A/ITGAE/MSN/ITGAM/MYH9/FGF2/RDX/EGF/SSH1/EGFR/CSK/ACTN1/ARPC5L/FGFR3/SSH2/DIAPH3/PAK1/PDGFC/SLC9A1/SOS2/PDGFD/PIK3R5/MYL7/ITGB4/VAV2/MYLK3/VAV1/ITGA7/SSH3/FGD3/VAV3/KRAS/ITGAL/GIT1/PIP5K1A/MYL5/FGFR2/ITGB7/SCIN |
| Leishmania infection | 68 | -0.316361677 | -1.368132916 | 0.0422087702956758 | 0.0422087702956758 | 0.239651885972836 | 2848 | tags=29%, list=18%, signal=24% | HLA-DQA2/IFNG/ITGAM/IRAK1/TLR4/MAPK14/NFKBIA/IL10/NCF4/CR1/FCGR3A/TNF/FCGR2A/JAK1/HLA-DMA/C3/TLR2/NFKB1/NOS2/TGFB3 |
| Porphyrin and chlorophyll metabolism | 25 | 0.452025024129743 | 1.50320509182595 | 0.0440763063554658 | 0.0440763063554658 | 0.239651885972836 | 3052 | tags=32%, list=19%, signal=26% | CP/COX15/CPOX/FECH/UROS/UGT2B7/GUSB/HMOX2 |
| Graft-versus-host disease | 33 | -0.406958482 | -1.506066158 | 0.0450039728480792 | 0.0450039728480792 | 0.239651885972836 | 4810 | tags=48%, list=30%, signal=34% | HLA-DRA/HLA-DOA/HLA-E/HLA-C/HLA-G/GZMB/HLA-DQA2/IFNG/HLA-B/HLA-A/CD80/TNF/KLRC1/HLA-DMA/KLRD1/FASLG |
| Antigen processing and presentation | 63 | -0.324195641 | -1.366204847 | 0.0457890685836662 | 0.0457890685836662 | 0.239651885972836 | 4990 | tags=41%, list=31%, signal=29% | KLRC4/CTSL/CTSS/HLA-DRA/HLA-DOA/CD8B/HLA-E/HSPA1A/HLA-C/HLA-G/CTSB/CREB1/RFX5/HLA-DQA2/HLA-B/TAP2/KLRC2/HLA-A/NFYA/HSPA1L/HSPA8/KLRC1/HLA-DMA/LGMN/KLRD1/KLRC3 |
| Allograft rejection | 32 | -0.397983756 | -1.447196412 | 0.0461290721692811 | 0.0461290721692811 | 0.239651885972836 | 4810 | tags=53%, list=30%, signal=37% | HLA-DRA/HLA-DOA/HLA-E/HLA-C/HLA-G/IL12B/GZMB/HLA-DQA2/IFNG/HLA-B/HLA-A/CD80/IL10/TNF/CD40/HLA-DMA/FASLG |
| Oxidative phosphorylation | 123 | 0.287626089380607 | 1.33438725851507 | 0.0494233937397035 | 0.0494233937397035 | 0.248742738229429 | 3207 | tags=28%, list=20%, signal=23% | COX5A/ATP5ME/ATP6V1C1/ATP5MF/NDUFA11/COX15/ATP5F1C/NDUFS5/NDUFA4/UQCRHL/NDUFA6/NDUFV2/ATP5PF/SDHC/MT-CYB/NDUFA3/COX7B/ATP5PD/NDUFA10/ATP5MC3/COX4I1/COX6C/COX7A2L/UQCRFS1/UQCRH/ATP12A/UQCRC1/MT-ND3/COX8A/NDUFS2/ATP6V1E2/NDUFB1/NDUFS7/ATP5F1E/ATP5MG |

**Supplementary Table 9.** GSEA of MELK in the LSIL vs. HSIL group

| Description | Set Size | Enrichment Score | NES | *p*-value | *p*.adjust | *q*-value | Rank | Leading edge | Core enrichment |
| --- | --- | --- | --- | --- | --- | --- | --- | --- | --- |
| Ribosome | 85 | 0.412360148709044 | 1.9769215002778 | 1.87129894935118e-05 | 1.87129894935118e-05 | 0.0032895465741226 | 3638 | tags=44%, list=22%, signal=34% | RPL36A/RPS15A/RPL22/RPS20/RPL30/RPL36AL/RPL29/RPL13/RPL9/RPSA/RPL32/RPL17/RPS6/RPL7A/RPL8/RPL41/RPL11/RPL18/RPL26/RPL27A/RPS11/RPS15/RPL3L/RPS19/RPLP2/RPL13A/RPS27/RPL37/RPS25/RPL27/RPS29/RPS18/RPS9/RPL21/RPL15/RPS17/RPL5 |
| Axon guidance | 125 | -0.373238087 | -1.661804246 | 0.000752160073929087 | 0.000752160073929087 | 0.0661109117611355 | 2943 | tags=33%, list=18%, signal=27% | SEMA3G/EFNA1/EPHA3/EFNA2/PPP3R1/NFAT5/DCC/NFATC3/MAPK3/EFNB2/NTNG1/SEMA4F/MAPK1/RHOD/RGS3/SEMA3F/EPHA1/PLXNA3/HRAS/UNC5B/SEMA3A/NTN4/ROBO2/PPP3CA/PPP3CB/NGEF/ABLIM1/EPHA6/SEMA4G/DPYSL2/ABL1/CDC42/PLXNA2/GSK3B/SEMA7A/EFNA5/SEMA6A/PAK6/SEMA5B/L1CAM/PLXNB3 |
| Parkinson's disease | 121 | 0.311566953683187 | 1.60552315147576 | 0.00158275752424454 | 0.00158275752424454 | 0.092744037385557 | 3878 | tags=37%, list=24%, signal=28% | UQCRQ/NDUFS4/SLC25A6/NDUFA8/ATP5PF/NDUFB6/NDUFV2/SDHB/SLC6A3/COX7C/UBE2L6/ATP5PO/NDUFB4/VDAC2/PARK7/NDUFB10/NDUFA3/UQCRHL/CASP3/MT-ATP8/UCHL1/SLC25A4/UQCRFS1/NDUFA4/NDUFC2/COX5A/NDUFB8/PRKN/ATP5MC2/NDUFA2/ATP5MC3/UQCRB/UQCR11/COX6B1/UBA7/NDUFB1/COX4I1/NDUFS8/ATP5PB/ATP5F1A/NDUFB7/NDUFA1/ATP5MC1/NDUFB5/MT-ND6 |
| Other glycan degradation | 14 | -0.621702747 | -1.727798136 | 0.00703657538832373 | 0.00703657538832373 | 0.309238971013175 | 2886 | tags=64%, list=18%, signal=53% | FUCA1/ENGASE/MAN2B2/MAN2B1/HEXA/GLB1/MANBA/NEU4/FUCA2 |
| Oxidative phosphorylation | 123 | 0.273855862325102 | 1.41733333146249 | 0.0102522648835599 | 0.0102522648835599 | 0.316833443784109 | 4517 | tags=41%, list=28%, signal=30% | UQCRQ/NDUFS4/NDUFA8/ATP5PF/ATP5MF/NDUFB6/NDUFV2/SDHB/COX7C/ATP5PO/NDUFB4/NDUFB10/NDUFA3/UQCRHL/TCIRG1/MT-ATP8/UQCRFS1/NDUFA4/NDUFC2/COX5A/PPA1/NDUFB8/ATP6V0C/ATP5MC2/NDUFA2/ATP5MC3/ATP6V1A/UQCRB/UQCR11/COX6B1/NDUFB1/COX4I1/NDUFS8/ATP5PB/ATP5F1A/NDUFB7/ATP6V0A4/NDUFA1/ATP5MC1/NDUFB5/MT-ND6/NDUFA9/ATP6V1C1/ATP5MG/COX17/ATP6V0A2/ATP6V0E1/NDUFS2/ATP6V1B1/COX11 |
| Butanoate metabolism | 30 | -0.489937802 | -1.667228915 | 0.0108140756261642 | 0.0108140756261642 | 0.316833443784109 | 4750 | tags=57%, list=29%, signal=40% | AKR1B10/ACADS/ACAT2/BDH2/ACSM3/L2HGDH/PDHA1/ACAT1/GAD1/ALDH3A2/AACS/OXCT1/ALDH2/ALDH5A1/ACSM5/OXCT2/ACSM1 |
| Tyrosine metabolism | 38 | -0.428550885 | -1.548426266 | 0.0130509194453072 | 0.0130509194453072 | 0.327744894340797 | 4715 | tags=53%, list=29%, signal=37% | LCMT2/GOT2/TPO/ALDH3A1/GSTZ1/TAT/TRMT11/HPD/FAH/TYRP1/DBH/HEMK1/ADH6/DCT/METTL6/ADH1B/COMT/AOC3/ADH1A/MAOB |
| Systemic lupus erythematosus | 111 | 0.277164053412867 | 1.40705557449958 | 0.0151367531410449 | 0.0151367531410449 | 0.332610233494013 | 3401 | tags=33%, list=21%, signal=27% | H2AC16/H4C4/H3C4/H2AC8/H2BC5/TNF/ACTN3/HLA-DQA1/H2AJ/H2AC14/C1QA/HLA-DMB/H2AX/C1S/CD40LG/HLA-DQB1/H3C12/H3C2/H4C8/C2/CD80/C4A/H3C15/HLA-DRB5/H2AC15/H2AB3/MACROH2A1/ELANE/SNRPD3/H2AC17/H3C1/H4C3/H3C14/H3C3/C1R/ACTN2/C1QC |
| Alzheimer's disease | 157 | 0.247245644887859 | 1.31477120208591 | 0.0176053593426977 | 0.0176053593426977 | 0.343870761430469 | 3899 | tags=35%, list=24%, signal=27% | UQCRQ/NDUFS4/NDUFA8/ATP5PF/PSEN2/NDUFB6/NDUFV2/APH1A/SDHB/COX7C/ATP5PO/NDUFB4/ADAM17/MME/TNF/NDUFB10/NDUFA3/UQCRHL/CASP3/GNAQ/CACNA1D/MT-ATP8/PPP3CC/UQCRFS1/BACE2/NDUFA4/NDUFC2/COX5A/NDUFB8/ATP2A1/CACNA1C/ATP5MC2/NDUFA2/BID/ATP5MC3/UQCRB/APOE/UQCR11/COX6B1/CALM2/NDUFB1/COX4I1/IDE/NDUFS8/ATP5PB/CDK5/ATP5F1A/NDUFB7/CACNA1F/CASP8/NDUFA1/CAPN2/ATP5MC1/NDUFB5/ERN1 |
| T cell receptor signaling pathway | 102 | -0.331007084 | -1.425942571 | 0.0279299805550866 | 0.0279299805550866 | 0.414888469182114 | 3918 | tags=43%, list=24%, signal=33% | AKT1/ZAP70/CD247/PIK3CB/RHOA/SOS2/NFATC4/AKT2/PAK3/AKT3/PTPRC/VAV3/NFKB1/NFATC1/MAPK11/PPP3R1/NFAT5/CHUK/NFATC3/MAPK3/RASGRP1/GRAP2/MAPK1/CD3E/CTLA4/DLG1/PRKCQ/HRAS/TEC/IFNG/MAP2K7/PPP3CA/PPP3CB/NFKBIA/CARD11/CDC42/FOS/MAP3K14/MALT1/GSK3B/PTPN6/SOS1/LAT/PAK6 |
| Retinol metabolism | 35 | -0.42353294 | -1.493374412 | 0.0281083029017942 | 0.0281083029017942 | 0.414888469182114 | 3627 | tags=37%, list=22%, signal=29% | DHRS3/CYP2A6/PNPLA4/DHRS9/RDH11/CYP3A7/ADH6/RDH16/ADH1B/UGT1A1/CYP26B1/ADH1A/BCO1 |
| Pentose phosphate pathway | 23 | 0.447949504198585 | 1.59093137480274 | 0.0283217278363839 | 0.0283217278363839 | 0.414888469182114 | 1990 | tags=30%, list=12%, signal=27% | ALDOB/ALDOA/RPEL1/TKT/FBP1/PGM2/RPIA |
| Adherens junction | 72 | -0.347034605 | -1.414426093 | 0.0426409903713893 | 0.0426409903713893 | 0.473297111968258 | 5497 | tags=56%, list=34%, signal=37% | EP300/SRC/ACTN1/WASL/IQGAP1/MET/CREBBP/PTPRM/PTPRB/SMAD3/SMAD2/CTNND1/RHOA/TGFBR1/CTNNA1/IGF1R/LMO7/SNAI1/PTPN1/SSX2IP/SORBS1/NECTIN4/LEF1/CSNK2B/MAPK3/BAIAP2/MAPK1/PARD3/TCF7/NLK/YES1/ACTB/AFDN/CDC42/FGFR1/WASF1/CSNK2A1/PTPN6/FARP2/WASF3 |
| Huntington's disease | 169 | 0.234353597060482 | 1.25791571483801 | 0.0445876523703922 | 0.0445876523703922 | 0.473297111968258 | 3770 | tags=31%, list=23%, signal=24% | UQCRQ/NDUFS4/SLC25A6/NDUFA8/ATP5PF/NDUFB6/NDUFV2/SDHB/COX7C/TBPL1/ATP5PO/NDUFB4/CREB3L1/VDAC2/NDUFB10/NDUFA3/UQCRHL/CASP3/GNAQ/MT-ATP8/SLC25A4/POLR2A/POLR2K/GPX1/UQCRFS1/DNAH1/NDUFA4/NDUFC2/COX5A/DCTN1/NDUFB8/POLR2I/POLR2J2/ATP5MC2/NDUFA2/SP1/ATP5MC3/IFT57/UQCRB/UQCR11/COX6B1/POLR2G/NDUFB1/COX4I1/NDUFS8/ATP5PB/HTT/ATP5F1A/NDUFB7/DCTN2/CASP8/NDUFA1/ATP5MC1 |
| Spliceosome | 126 | -0.303204743 | -1.350180259 | 0.0455136540962289 | 0.0455136540962289 | 0.473297111968258 | 4615 | tags=37%, list=29%, signal=26% | ACIN1/SRSF9/XAB2/PRPF6/TCERG1/CDC40/U2AF1/SNU13/CRNKL1/LSM5/DHX16/THOC2/SMNDC1/SF3A1/LSM7/U2SURP/SRSF5/SF3B6/HNRNPC/DDX5/DHX38/LSM4/SART1/SF3B1/PRPF38B/PPIE/HNRNPA3/SNW1/HSPA8/SRSF2/HSPA1A/THOC1/LSM2/DHX8/PPIH/HSPA1L/SNRPA/DHX15/RBM22/HSPA1B/CHERP/CDC5L/PRPF8/PHF5A/SRSF3/HSPA6 |
| Endocytosis | 179 | -0.28157827 | -1.317310717 | 0.0474452554744526 | 0.0474452554744526 | 0.473297111968258 | 4493 | tags=36%, list=28%, signal=27% | PRKCI/SH3GL3/NTRK1/SMURF2/ACAP1/RAB5C/CHMP2B/ARFGAP1/MDM2/CLTCL1/ASAP3/KDR/CLTC/AGAP2/PLD2/CHMP4C/RAB22A/GRK2/IGF1R/SH3GL2/USP8/TSG101/ITCH/PLD1/TFRC/EPN2/ARAP1/CHMP5/SMAP1/PARD6G/HLA-C/ACAP3/PARD3/PRKCZ/HRAS/GRK6/PIP5K1C/HSPA8/VPS37B/RUFY1/GRK3/HSPA1A/DNM1L/AP2B1/RAB11FIP3/VPS45/HSPA1L/FGFR2/IQSEC2/HSPA1B/CDC42/FGFR3/NEDD4/TRAF6/RBSN/STAM2/EHD1/GRK4/FLT1/GRK5/HLA-E/HLA-G/HSPA6/SMAP2/HLA-F |
| Small cell lung cancer | 84 | -0.330645358 | -1.387369353 | 0.0482573726541555 | 0.0482573726541555 | 0.473297111968258 | 5116 | tags=45%, list=32%, signal=31% | LAMB2/E2F2/MYC/AKT1/LAMA2/CYCS/PIK3CB/LAMC3/CDKN1B/CCNE2/PTEN/AKT2/AKT3/RB1/COL4A2/NFKB1/PIAS4/CASP9/LAMA4/RXRG/COL4A4/PIAS3/CHUK/RXRB/RARB/ITGA6/CDK6/PIAS2/PTGS2/TRAF5/NFKBIA/LAMB4/TRAF6/LAMA1/BCL2/TRAF4/ITGA2B/E2F3 |
| MAPK signaling pathway | 241 | -0.26459185 | -1.284376675 | 0.0484633569739953 | 0.0484633569739953 | 0.473297111968258 | 4939 | tags=38%, list=31%, signal=27% | MYC/AKT1/ELK1/NFKB2/MAP4K2/MAP3K1/PLA2G4B/MAPK10/TAOK1/FAS/TAOK3/SOS2/MAP3K11/NTRK1/MAP3K13/FGF2/NFATC4/DAXX/AKT2/CACNA1G/AKT3/TGFBR1/GADD45B/MAP3K3/MAPKAPK5/NFKB1/TGFB2/LAMTOR3/FGF13/MAP3K6/MAPK11/ATF2/SRF/RASGRP2/MECOM/PPP3R1/RPS6KA2/CHUK/ATF4/MAPT/MAPK3/RASGRP1/DUSP16/MAPK1/FGF22/MAP4K4/RAPGEF2/STK3/TNFRSF1A/HRAS/NLK/HSPA8/CDC25B/PLA2G6/FGF20/CACNA1E/HSPA1A/PLA2G3/DUSP8/MAP2K5/PDGFB/MAP2K7/PPP3CA/PPP3CB/HSPA1L/DUSP5/FGFR2/PRKX/BDNF/PPM1A/CACNG4/TAB1/HSPA1B/CDC42/FGFR3/MAP3K4/FGFR1/FOS/MAP3K14/TRAF6/RPS6KA6/CACNB1/PRKACA/DDIT3/MAPK8IP3/MEF2C/STK4/SOS1/HSPA6/GNG12/FGF10 |

**Supplementary Table 10.** GSEA of MELK in the HSIL vs. SCC group

| Description | Set Size | Enrichment Score | NES | *p*-value | *p*.adjust | *q*-value | Rank | Leading edge | Core enrichment |
| --- | --- | --- | --- | --- | --- | --- | --- | --- | --- |
| Antigen processing and presentation | 63 | 0.50660213688768 | 1.84971244118428 | 0.000132833555923253 | 0.000132833555923253 | 0.0230710912919334 | 2988 | tags=40%, list=18%, signal=32% | HLA-F/KIR3DL2/KIR2DL4/LTA/TAPBP/HLA-DPA1/HLA-DOA/HSPA1B/NFYC/HLA-C/HLA-B/CALR/PSME2/HSPA4/HSPA1A/HLA-DQA2/HLA-DQA1/KLRC3/CD74/HLA-DRA/HLA-DRB5/KLRC2/HLA-DMB/HSPA6/HLA-DPB1 |
| Leishmania infection | 68 | 0.461496599914396 | 1.69575451620332 | 0.000694549278717726 | 0.000694549278717726 | 0.0603161215728552 | 3913 | tags=44%, list=24%, signal=34% | TAB1/FCGR1A/TLR4/IL12A/HLA-DPA1/NFKBIA/FCGR3B/HLA-DOA/TRAF6/RELA/MARCKSL1/NCF4/HLA-DQA2/HLA-DQA1/IL10/C3/HLA-DRA/TLR2/FOS/HLA-DRB5/ITGAM/HLA-DMB/HLA-DPB1/PTPN6/JUN/MAPK11/IFNGR2/PRKCB/IRAK1/MAPK3 |
| Neurotrophin signaling pathway | 126 | 0.390589835285105 | 1.5606764555887 | 0.00134099614389027 | 0.00134099614389027 | 0.0615805407045258 | 4073 | tags=40%, list=25%, signal=30% | BCL2/PIK3R2/YWHAH/MAGED1/NFKBIA/PIK3CD/CAMK2D/CDC42/TRAF6/RELA/SHC4/SHC1/IRS4/SH2B3/FOXO3/CAMK2G/SOS2/MAP2K1/PIK3R5/PTPN11/SOS1/NFKBIE/RIPK2/BDNF/RPS6KA3/RPS6KA5/AKT1/TP53/ZNF274/ARHGDIB/CSK/CALM3/JUN/NTRK1/CALML5/MAPK11/IKBKB/BAD/CRK/SH2B1/PIK3R3/NTRK3/IRAK3/IRAK1/MAP2K5/MAP3K3/MAPK3/MAP2K2/RPS6KA4/AKT2 |
| Chronic myeloid leukemia | 73 | 0.438202686945659 | 1.63584946307134 | 0.00141821851319514 | 0.00141821851319514 | 0.0615805407045258 | 5515 | tags=56%, list=34%, signal=37% | PIK3R2/NFKBIA/PIK3CD/E2F1/CDKN1A/RELA/SHC4/SHC1/CDK6/IKBKG/SOS2/MAP2K1/PIK3R5/PTPN11/SOS1/CTBP2/TGFBR2/AKT1/TP53/RB1/IKBKB/BAD/CRK/PIK3R3/SMAD3/CBLC/MAPK3/MAP2K2/AKT2/GAB2/MDM2/MYC/KRAS/MAPK1/CTBP1/SMAD4/BCR/TGFB3/STAT5B/NFKB1/E2F3 |
| Glioma | 64 | 0.44661534289377 | 1.63099488573092 | 0.00341767501603931 | 0.00341767501603931 | 0.11871923739926 | 4168 | tags=47%, list=26%, signal=35% | IGF1R/PIK3R2/PDGFB/PTEN/MTOR/PIK3CD/CAMK2D/E2F1/CDKN1A/SHC4/SHC1/CDK6/CAMK2G/SOS2/MAP2K1/PIK3R5/SOS1/AKT1/TP53/EGFR/RB1/CALM3/CALML5/EGF/PIK3R3/PRKCB/MAPK3/MAP2K2/AKT2/MDM2 |
| T cell receptor signaling pathway | 102 | 0.382287971892344 | 1.48804517837588 | 0.00762729324636246 | 0.00762729324636246 | 0.197164799604792 | 4073 | tags=41%, list=25%, signal=31% | PIK3R2/CARD11/PAK6/PDCD1/CD3E/PPP3CA/NFKBIA/GRAP2/PIK3CD/TEC/PRKCQ/CDC42/RELA/MAP3K14/PAK4/IKBKG/SOS2/MAP2K1/PIK3R5/MALT1/SOS1/NFKBIE/PAK1/AKT1/IL10/FOS/PTPN6/JUN/NFATC2/ICOS/VAV3/MAPK11/IKBKB/LCK/PIK3R3/CBLC/NFAT5/BCL10/MAPK3/MAP2K2/VAV1/AKT2 |
| Hypertrophic cardiomyopathy (HCM) | 69 | -0.287302075 | -1.456855026 | 0.00946014176814317 | 0.00946014176814317 | 0.197164799604792 | 3680 | tags=39%, list=23%, signal=30% | CACNA2D3/SGCA/ITGB5/PRKAG1/CACNB2/CACNB4/MYBPC3/SGCB/TNNT2/EMD/SGCD/ITGB1/IGF1/CACNG4/TNF/TNNC1/ITGA5/CACNA1F/CACNA2D2/PRKAA2/ITGA3/ITGA2/CACNA2D4/ITGB6/MYL3/CACNA1C/CACNA1D |
| Systemic lupus erythematosus | 111 | 0.368307218859142 | 1.44652178614959 | 0.00984053284378777 | 0.00984053284378777 | 0.197164799604792 | 3099 | tags=31%, list=19%, signal=25% | H2BC18/H2BC13/H2AC21/FCGR1A/ELANE/GRIN2A/HLA-DPA1/FCGR3B/SNRPD3/HLA-DOA/H3C15/TRIM21/H4C15/H4C5/H3C8/H4C14/H3C14/H3-5/C4B/H3C12/C8G/H2BC11/HLA-DQA2/HLA-DQA1/C1QA/IL10/C3/HLA-DRA/H3C11/HLA-DRB5/HLA-DMB/C7/HLA-DPB1/H2BC21 |
| Allograft rejection | 32 | 0.484142342693745 | 1.57420510254186 | 0.0102167214340665 | 0.0102167214340665 | 0.197164799604792 | 3511 | tags=47%, list=22%, signal=37% | HLA-F/IL12A/HLA-DPA1/HLA-DOA/HLA-C/HLA-B/HLA-DQA2/HLA-DQA1/IL10/HLA-DRA/HLA-DRB5/HLA-DMB/HLA-DPB1/GZMB/CD40 |
| Autoimmune thyroid disease | 32 | 0.464392167299365 | 1.5099867433111 | 0.0175015426312182 | 0.0175015426312182 | 0.283745556844358 | 3511 | tags=44%, list=22%, signal=34% | HLA-F/HLA-DPA1/HLA-DOA/HLA-C/HLA-B/HLA-DQA2/HLA-DQA1/IL10/HLA-DRA/HLA-DRB5/HLA-DMB/HLA-DPB1/GZMB/CD40 |
| Graft-versus-host disease | 33 | 0.47161291538687 | 1.54281563592069 | 0.017970551933476 | 0.017970551933476 | 0.283745556844358 | 3279 | tags=39%, list=20%, signal=31% | HLA-F/KIR3DL2/HLA-DPA1/HLA-DOA/HLA-C/HLA-B/HLA-DQA2/HLA-DQA1/HLA-DRA/HLA-DRB5/HLA-DMB/HLA-DPB1/GZMB |
| Toll-like receptor signaling pathway | 86 | 0.37712007531639 | 1.43862076327807 | 0.0207318584141029 | 0.0207318584141029 | 0.300066371783069 | 4073 | tags=41%, list=25%, signal=31% | TAB1/PIK3R2/TLR4/IRF7/IL12A/CXCL11/NFKBIA/PIK3CD/RIPK1/CXCL9/SPP1/CCL5/TRAF6/TLR5/RELA/TLR7/TLR1/IKBKG/MAP2K4/MAP2K1/PIK3R5/AKT1/TLR2/FOS/JUN/CD14/MAPK11/IKBKB/CD40/PIK3R3/IRAK1/TLR8/MAPK3/MAP2K2/AKT2 |
| Prostate cancer | 87 | 0.36481465676814 | 1.39496956520269 | 0.0284746304782041 | 0.0284746304782041 | 0.380430285741188 | 4765 | tags=45%, list=29%, signal=32% | IGF1R/BCL2/PIK3R2/PDGFB/PTEN/NFKBIA/MTOR/PIK3CD/E2F1/CDKN1A/RELA/IKBKG/SOS2/MAP2K1/PIK3R5/SOS1/PDGFC/AKT1/TP53/EGFR/RB1/CREB3L4/EGF/CTNNB1/IKBKB/BAD/PIK3R3/PDGFD/HSP90AB1/MAPK3/MAP2K2/ERBB2/AKT2/MDM2/LEF1/KRAS/FGFR1/NKX3-1/MAPK1 |
| Type I diabetes mellitus | 38 | 0.431747540380512 | 1.43656945098546 | 0.0385064177362894 | 0.0385064177362894 | 0.403230613339691 | 3279 | tags=37%, list=20%, signal=29% | HLA-F/IL12A/LTA/HLA-DPA1/HLA-DOA/HLA-C/HLA-B/HLA-DQA2/HLA-DQA1/HLA-DRA/HLA-DRB5/HLA-DMB/HLA-DPB1/GZMB |
| Pancreatic cancer | 70 | 0.374639233629072 | 1.38771368347809 | 0.0425531914893617 | 0.0425531914893617 | 0.403230613339691 | 4073 | tags=37%, list=25%, signal=28% | PIK3R2/PIK3CD/BRCA2/E2F1/CDC42/RELA/CDK6/IKBKG/MAP2K1/STAT3/PIK3R5/TGFBR2/AKT1/TP53/EGFR/RB1/VEGFB/EGF/IKBKB/BAD/ARHGEF6/PIK3R3/SMAD3/MAPK3/ERBB2/AKT2 |
| Olfactory transduction | 43 | 0.41587540107205 | 1.41688577938822 | 0.0425776754890679 | 0.0425776754890679 | 0.403230613339691 | 3205 | tags=33%, list=20%, signal=26% | GRK3/CAMK2D/OR2L2/ADCY3/OR1L8/PRKX/OR5K2/OR52N4/CAMK2G/PRKACB/OR2A1/OR2B6/CALM3/CALML5 |
| ErbB signaling pathway | 84 | 0.356117046908922 | 1.35192246245362 | 0.0437158469945355 | 0.0437158469945355 | 0.403230613339691 | 4255 | tags=42%, list=26%, signal=31% | PIK3R2/PAK6/MTOR/PIK3CD/CAMK2D/NRG4/CDKN1A/ABL2/SHC4/SHC1/PAK4/MAP2K4/CAMK2G/SOS2/MAP2K1/BTC/PIK3R5/SOS1/PAK1/AKT1/EGFR/JUN/EGF/BAD/CRK/PIK3R3/PRKCB/EREG/CBLC/RPS6KB2/MAPK3/MAP2K2/ERBB2/AKT2/MYC |
| Spliceosome | 126 | 0.329959532873135 | 1.3184164761386 | 0.043979057591623 | 0.043979057591623 | 0.403230613339691 | 4154 | tags=31%, list=26%, signal=23% | SNW1/SNRPD3/ACIN1/SNU13/HSPA1B/DHX38/LSM2/PCBP1/SF3B3/PHF5A/HSPA1A/PRPF8/HNRNPA3/CRNKL1/SNRPG/HNRNPA1/PPIH/SRSF10/SF3A1/SRSF5/HNRNPA1L2/SNRNP27/CCDC12/PRPF40A/HSPA6/SRSF2/DHX16/PLRG1/SNRPB2/DHX8/SF3B5/SNRNP70/LSM3/SNRPA1/SF3A3/THOC2/TXNL4A/PRPF19/SNRPA |
| Arrhythmogenic right ventricular cardiomyopathy (ARVC) | 64 | -0.279856498 | -1.386010713 | 0.0458334957382379 | 0.0458334957382379 | 0.403230613339691 | 3680 | tags=38%, list=23%, signal=29% | CACNA2D3/SGCA/ITGB5/CACNB2/CACNB4/DSG2/SGCB/EMD/SGCD/ITGB1/CACNG4/TCF7L1/TCF7L2/ITGA5/CACNA1F/CACNA2D2/ACTN3/ITGA3/ITGA2/CACNA2D4/ITGB6/CACNA1C/CACNA1D/GJA1 |
| Adipocytokine signaling pathway | 59 | 0.379279150095665 | 1.36308019663107 | 0.0464326160815402 | 0.0464326160815402 | 0.403230613339691 | 2497 | tags=31%, list=15%, signal=26% | ACSL5/CPT1B/NFKBIA/ADIPOR2/MTOR/PRKCQ/RELA/IRS4/RXRA/CAMKK1/IKBKG/ADIPOR1/STAT3/PTPN11/NFKBIE/AKT1/TNFRSF1A/LEPR |

**Supplementary Table 11**. ceRNA network analysis of hub genes

| Hub gene | miRNA | lncRNA |
| --- | --- | --- |
| BUB1B | hsa-miR-192-5p | XIST |
| BUB1B | hsa-miR-193b-3p | XIST |
| BUB1B | hsa-miR-215-5p | XIST |
| BUB1B | hsa-miR-192-5p | KCNQ1OT1 |
| BUB1B | hsa-miR-193b-3p | KCNQ1OT1 |
| BUB1B | hsa-miR-215-5p | KCNQ1OT1 |
| BUB1B | hsa-miR-193b-3p | NEAT1 |
| BUB1B | hsa-miR-22-3p | NEAT1 |
| BUB1B | hsa-miR-22-3p | SNHG14 |
| BUB1B | hsa-miR-22-3p | NORAD |
| BUB1B | hsa-miR-22-3p | OIP5-AS1 |
| BUB1B | hsa-miR-22-3p | FGD5-AS1 |
| BUB1B | hsa-miR-22-3p | SNHG16 |
| BUB1B | hsa-miR-22-3p | MIR34AHG |
| BUB1B | hsa-miR-22-3p | MALAT1 |
| BUB1B | hsa-miR-22-3p | H19 |
| BUB1B | hsa-miR-192-5p | LINC01089 |
| BUB1B | hsa-miR-193b-3p | LINC01089 |
| BUB1B | hsa-miR-215-5p | LINC01089 |
| BUB1B | hsa-miR-193b-3p | MIR4435-2HG |
| BUB1B | hsa-miR-193b-3p | SNHG7 |
| BUB1B | hsa-miR-192-5p | SOX2-OT |
| BUB1B | hsa-miR-215-5p | SOX2-OT |
| BUB1B | hsa-miR-192-5p | DNAJC27-AS1 |
| BUB1B | hsa-miR-215-5p | DNAJC27-AS1 |
| BUB1B | hsa-miR-192-5p | MIR122HG |
| BUB1B | hsa-miR-215-5p | MIR122HG |
| BUB1B | hsa-miR-193b-3p | MIR194-2HG |
| BUB1B | hsa-miR-215-5p | MIR194-2HG |
| BUB1B | hsa-miR-193b-3p | MIRLET7BHG |
| BUB1B | hsa-miR-22-3p | MIRLET7BHG |
| BUB1B | hsa-miR-22-3p | LINC00630 |
| BUB1B | hsa-miR-193b-3p | LINC01184 |
| BUB1B | hsa-miR-193b-3p | CYTOR |
| BUB1B | hsa-miR-193b-3p | ZFAS1 |
| BUB1B | hsa-miR-22-3p | LINC00963 |
| BUB1B | hsa-miR-22-3p | SLC25A25-AS1 |
| KIF14 | hsa-miR-1277-5p | XIST |
| KIF14 | hsa-miR-155-5p | XIST |
| KIF14 | hsa-miR-16-5p | XIST |
| KIF14 | hsa-miR-186-5p | XIST |
| KIF14 | hsa-miR-192-5p | XIST |
| KIF14 | hsa-miR-215-5p | XIST |
| KIF14 | hsa-miR-615-3p | XIST |
| KIF14 | hsa-miR-1277-5p | KCNQ1OT1 |
| KIF14 | hsa-miR-155-5p | KCNQ1OT1 |
| KIF14 | hsa-miR-16-5p | KCNQ1OT1 |
| KIF14 | hsa-miR-186-5p | KCNQ1OT1 |
| KIF14 | hsa-miR-192-5p | KCNQ1OT1 |
| KIF14 | hsa-miR-215-5p | KCNQ1OT1 |
| KIF14 | hsa-miR-1277-5p | NEAT1 |
| KIF14 | hsa-miR-16-5p | NEAT1 |
| KIF14 | hsa-miR-186-5p | NEAT1 |
| KIF14 | hsa-miR-615-3p | NEAT1 |
| KIF14 | hsa-miR-1277-5p | SNHG14 |
| KIF14 | hsa-miR-186-5p | SNHG14 |
| KIF14 | hsa-miR-1277-5p | NORAD |
| KIF14 | hsa-miR-155-5p | NORAD |
| KIF14 | hsa-miR-615-3p | NORAD |
| KIF14 | hsa-miR-1277-5p | OIP5-AS1 |
| KIF14 | hsa-miR-155-5p | OIP5-AS1 |
| KIF14 | hsa-miR-16-5p | FGD5-AS1 |
| KIF14 | hsa-miR-615-3p | FGD5-AS1 |
| KIF14 | hsa-miR-16-5p | SNHG16 |
| KIF14 | hsa-miR-1277-5p | MIR34AHG |
| KIF14 | hsa-miR-16-5p | MIR34AHG |
| KIF14 | hsa-miR-1277-5p | LINC01106 |
| KIF14 | hsa-miR-155-5p | MIR17HG |
| KIF14 | hsa-miR-1277-5p | EPB41L4A-AS1 |
| KIF14 | hsa-miR-155-5p | MALAT1 |
| KIF14 | hsa-miR-192-5p | LINC01089 |
| KIF14 | hsa-miR-215-5p | LINC01089 |
| KIF14 | hsa-miR-615-3p | LINC01089 |
| KIF14 | hsa-miR-16-5p | MIR4435-2HG |
| KIF14 | hsa-miR-186-5p | SNHG7 |
| KIF14 | hsa-miR-186-5p | SOX2-OT |
| KIF14 | hsa-miR-192-5p | SOX2-OT |
| KIF14 | hsa-miR-215-5p | SOX2-OT |
| KIF14 | hsa-miR-192-5p | DNAJC27-AS1 |
| KIF14 | hsa-miR-215-5p | DNAJC27-AS1 |
| KIF14 | hsa-miR-192-5p | MIR122HG |
| KIF14 | hsa-miR-215-5p | MIR122HG |
| KIF14 | hsa-miR-215-5p | MIR194-2HG |
| KIF14 | hsa-miR-186-5p | MIRLET7BHG |
| KIF14 | hsa-miR-1277-5p | LINC00630 |
| KIF14 | hsa-miR-186-5p | LINC00630 |
| KIF14 | hsa-miR-16-5p | LINC01184 |
| KIF14 | hsa-miR-16-5p | MIR29B2CHG |
| KIF14 | hsa-miR-186-5p | MIR600HG |
| KIF14 | hsa-miR-1277-5p | NUTM2B-AS1 |
| KIF14 | hsa-miR-186-5p | NUTM2B-AS1 |
| KIF14 | hsa-miR-1277-5p | OTUD6B-AS1 |
| KIF14 | hsa-miR-155-5p | OTUD6B-AS1 |
| KIF14 | hsa-miR-1277-5p | PSMA3-AS1 |
| KIF14 | hsa-miR-186-5p | PSMA3-AS1 |
| KIF14 | hsa-miR-16-5p | SNHG25 |
| KIF14 | hsa-miR-186-5p | SNHG3 |
| KIF14 | hsa-miR-155-5p | ARHGAP5-AS1 |
| KIF14 | hsa-miR-16-5p | C1RL-AS1 |
| KIF14 | hsa-miR-1277-5p | CTBP1-DT |
| KIF14 | hsa-miR-1277-5p | DANCR |
| KIF14 | hsa-miR-1277-5p | DLEU1 |
| KIF14 | hsa-miR-186-5p | FTX |
| KIF14 | hsa-miR-16-5p | GABPB1-AS1 |
| KIF14 | hsa-miR-615-3p | GNAS-AS1 |
| KIF14 | hsa-miR-155-5p | INE1 |
| KIF14 | hsa-miR-16-5p | LINC00662 |
| KIF14 | hsa-miR-16-5p | LINC00894 |
| KIF14 | hsa-miR-16-5p | LINC00943 |
| KIF14 | hsa-miR-1277-5p | LINC01123 |
| KIF14 | hsa-miR-16-5p | LINC02035 |
| KIF14 | hsa-miR-1277-5p | LINC02389 |
| KIF14 | hsa-miR-155-5p | LINC-PINT |
| KIF14 | hsa-miR-186-5p | MAGI1-IT1 |
| KIF14 | hsa-miR-186-5p | MANEA-DT |
| KIF14 | hsa-miR-1277-5p | MIR124-2HG |
| KIF14 | hsa-miR-1277-5p | MIR155HG |
| KIF14 | hsa-miR-1277-5p | MIR3142HG |
| KIF14 | hsa-miR-16-5p | MIR503HG |
| KIF14 | hsa-miR-16-5p | NUTM2A-AS1 |
| KIF14 | hsa-miR-615-3p | OVCH1-AS1 |
| KIF14 | hsa-miR-16-5p | PAX8-AS1 |
| KIF14 | hsa-miR-615-3p | PVT1 |
| KIF14 | hsa-miR-1277-5p | SLC26A4-AS1 |
| KIF14 | hsa-miR-16-5p | SNHG12 |
| KIF14 | hsa-miR-16-5p | STX17-AS1 |
| KIF14 | hsa-miR-16-5p | THUMPD3-AS1 |
| KIF14 | hsa-miR-186-5p | TRAF3IP2-AS1 |
| KIF14 | hsa-miR-615-3p | ZNF236-DT |
| MELK | hsa-miR-106a-5p | XIST |
| MELK | hsa-miR-106b-5p | XIST |
| MELK | hsa-miR-17-5p | XIST |
| MELK | hsa-miR-193b-3p | XIST |
| MELK | hsa-miR-20a-5p | XIST |
| MELK | hsa-miR-20b-5p | XIST |
| MELK | hsa-miR-302a-3p | XIST |
| MELK | hsa-miR-302b-3p | XIST |
| MELK | hsa-miR-302c-3p | XIST |
| MELK | hsa-miR-302d-3p | XIST |
| MELK | hsa-miR-302e | XIST |
| MELK | hsa-miR-371a-5p | XIST |
| MELK | hsa-miR-372-3p | XIST |
| MELK | hsa-miR-373-3p | XIST |
| MELK | hsa-miR-519d-3p | XIST |
| MELK | hsa-miR-520a-3p | XIST |
| MELK | hsa-miR-520b | XIST |
| MELK | hsa-miR-520c-3p | XIST |
| MELK | hsa-miR-520d-3p | XIST |
| MELK | hsa-miR-520e | XIST |
| MELK | hsa-miR-520g-3p | XIST |
| MELK | hsa-miR-520h | XIST |
| MELK | hsa-miR-526b-3p | XIST |
| MELK | hsa-miR-93-5p | XIST |
| MELK | hsa-miR-106a-5p | KCNQ1OT1 |
| MELK | hsa-miR-106b-5p | KCNQ1OT1 |
| MELK | hsa-miR-17-5p | KCNQ1OT1 |
| MELK | hsa-miR-193b-3p | KCNQ1OT1 |
| MELK | hsa-miR-20a-5p | KCNQ1OT1 |
| MELK | hsa-miR-20b-5p | KCNQ1OT1 |
| MELK | hsa-miR-302a-3p | KCNQ1OT1 |
| MELK | hsa-miR-302b-3p | KCNQ1OT1 |
| MELK | hsa-miR-302c-3p | KCNQ1OT1 |
| MELK | hsa-miR-302d-3p | KCNQ1OT1 |
| MELK | hsa-miR-302e | KCNQ1OT1 |
| MELK | hsa-miR-371a-5p | KCNQ1OT1 |
| MELK | hsa-miR-372-3p | KCNQ1OT1 |
| MELK | hsa-miR-373-3p | KCNQ1OT1 |
| MELK | hsa-miR-4712-5p | KCNQ1OT1 |
| MELK | hsa-miR-519d-3p | KCNQ1OT1 |
| MELK | hsa-miR-520a-3p | KCNQ1OT1 |
| MELK | hsa-miR-520b | KCNQ1OT1 |
| MELK | hsa-miR-520c-3p | KCNQ1OT1 |
| MELK | hsa-miR-520d-3p | KCNQ1OT1 |
| MELK | hsa-miR-520e | KCNQ1OT1 |
| MELK | hsa-miR-526b-3p | KCNQ1OT1 |
| MELK | hsa-miR-770-5p | KCNQ1OT1 |
| MELK | hsa-miR-93-5p | KCNQ1OT1 |
| MELK | hsa-miR-106a-5p | NEAT1 |
| MELK | hsa-miR-106b-5p | NEAT1 |
| MELK | hsa-miR-17-5p | NEAT1 |
| MELK | hsa-miR-193b-3p | NEAT1 |
| MELK | hsa-miR-20a-5p | NEAT1 |
| MELK | hsa-miR-20b-5p | NEAT1 |
| MELK | hsa-miR-302a-3p | NEAT1 |
| MELK | hsa-miR-302b-3p | NEAT1 |
| MELK | hsa-miR-302c-3p | NEAT1 |
| MELK | hsa-miR-302d-3p | NEAT1 |
| MELK | hsa-miR-302e | NEAT1 |
| MELK | hsa-miR-371a-5p | NEAT1 |
| MELK | hsa-miR-372-3p | NEAT1 |
| MELK | hsa-miR-373-3p | NEAT1 |
| MELK | hsa-miR-4712-5p | NEAT1 |
| MELK | hsa-miR-519d-3p | NEAT1 |
| MELK | hsa-miR-520a-3p | NEAT1 |
| MELK | hsa-miR-520b | NEAT1 |
| MELK | hsa-miR-520c-3p | NEAT1 |
| MELK | hsa-miR-520d-3p | NEAT1 |
| MELK | hsa-miR-520e | NEAT1 |
| MELK | hsa-miR-520g-3p | NEAT1 |
| MELK | hsa-miR-520h | NEAT1 |
| MELK | hsa-miR-526b-3p | NEAT1 |
| MELK | hsa-miR-770-5p | NEAT1 |
| MELK | hsa-miR-93-5p | NEAT1 |
| MELK | hsa-miR-106a-5p | SNHG14 |
| MELK | hsa-miR-106b-5p | SNHG14 |
| MELK | hsa-miR-17-5p | SNHG14 |
| MELK | hsa-miR-20a-5p | SNHG14 |
| MELK | hsa-miR-20b-5p | SNHG14 |
| MELK | hsa-miR-302a-3p | SNHG14 |
| MELK | hsa-miR-302b-3p | SNHG14 |
| MELK | hsa-miR-302c-3p | SNHG14 |
| MELK | hsa-miR-302d-3p | SNHG14 |
| MELK | hsa-miR-302e | SNHG14 |
| MELK | hsa-miR-372-3p | SNHG14 |
| MELK | hsa-miR-373-3p | SNHG14 |
| MELK | hsa-miR-4712-5p | SNHG14 |
| MELK | hsa-miR-519d-3p | SNHG14 |
| MELK | hsa-miR-520a-3p | SNHG14 |
| MELK | hsa-miR-520b | SNHG14 |
| MELK | hsa-miR-520c-3p | SNHG14 |
| MELK | hsa-miR-520d-3p | SNHG14 |
| MELK | hsa-miR-520e | SNHG14 |
| MELK | hsa-miR-520g-3p | SNHG14 |
| MELK | hsa-miR-520h | SNHG14 |
| MELK | hsa-miR-526b-3p | SNHG14 |
| MELK | hsa-miR-770-5p | SNHG14 |
| MELK | hsa-miR-93-5p | SNHG14 |
| MELK | hsa-miR-106a-5p | NORAD |
| MELK | hsa-miR-106b-5p | NORAD |
| MELK | hsa-miR-17-5p | NORAD |
| MELK | hsa-miR-20a-5p | NORAD |
| MELK | hsa-miR-20b-5p | NORAD |
| MELK | hsa-miR-302a-3p | NORAD |
| MELK | hsa-miR-302b-3p | NORAD |
| MELK | hsa-miR-302c-3p | NORAD |
| MELK | hsa-miR-302d-3p | NORAD |
| MELK | hsa-miR-302e | NORAD |
| MELK | hsa-miR-372-3p | NORAD |
| MELK | hsa-miR-373-3p | NORAD |
| MELK | hsa-miR-519d-3p | NORAD |
| MELK | hsa-miR-520a-3p | NORAD |
| MELK | hsa-miR-520b | NORAD |
| MELK | hsa-miR-520c-3p | NORAD |
| MELK | hsa-miR-520d-3p | NORAD |
| MELK | hsa-miR-520e | NORAD |
| MELK | hsa-miR-520g-3p | NORAD |
| MELK | hsa-miR-520h | NORAD |
| MELK | hsa-miR-526b-3p | NORAD |
| MELK | hsa-miR-93-5p | NORAD |
| MELK | hsa-miR-106a-5p | OIP5-AS1 |
| MELK | hsa-miR-106b-5p | OIP5-AS1 |
| MELK | hsa-miR-17-5p | OIP5-AS1 |
| MELK | hsa-miR-20a-5p | OIP5-AS1 |
| MELK | hsa-miR-20b-5p | OIP5-AS1 |
| MELK | hsa-miR-302a-3p | OIP5-AS1 |
| MELK | hsa-miR-302b-3p | OIP5-AS1 |
| MELK | hsa-miR-302c-3p | OIP5-AS1 |
| MELK | hsa-miR-302d-3p | OIP5-AS1 |
| MELK | hsa-miR-302e | OIP5-AS1 |
| MELK | hsa-miR-371a-5p | OIP5-AS1 |
| MELK | hsa-miR-372-3p | OIP5-AS1 |
| MELK | hsa-miR-373-3p | OIP5-AS1 |
| MELK | hsa-miR-519d-3p | OIP5-AS1 |
| MELK | hsa-miR-520a-3p | OIP5-AS1 |
| MELK | hsa-miR-520b | OIP5-AS1 |
| MELK | hsa-miR-520c-3p | OIP5-AS1 |
| MELK | hsa-miR-520d-3p | OIP5-AS1 |
| MELK | hsa-miR-520e | OIP5-AS1 |
| MELK | hsa-miR-520g-3p | OIP5-AS1 |
| MELK | hsa-miR-520h | OIP5-AS1 |
| MELK | hsa-miR-526b-3p | OIP5-AS1 |
| MELK | hsa-miR-93-5p | OIP5-AS1 |
| MELK | hsa-miR-106a-5p | FGD5-AS1 |
| MELK | hsa-miR-106b-5p | FGD5-AS1 |
| MELK | hsa-miR-17-5p | FGD5-AS1 |
| MELK | hsa-miR-20a-5p | FGD5-AS1 |
| MELK | hsa-miR-20b-5p | FGD5-AS1 |
| MELK | hsa-miR-302a-3p | FGD5-AS1 |
| MELK | hsa-miR-302b-3p | FGD5-AS1 |
| MELK | hsa-miR-302c-3p | FGD5-AS1 |
| MELK | hsa-miR-302d-3p | FGD5-AS1 |
| MELK | hsa-miR-302e | FGD5-AS1 |
| MELK | hsa-miR-372-3p | FGD5-AS1 |
| MELK | hsa-miR-373-3p | FGD5-AS1 |
| MELK | hsa-miR-519d-3p | FGD5-AS1 |
| MELK | hsa-miR-520a-3p | FGD5-AS1 |
| MELK | hsa-miR-520b | FGD5-AS1 |
| MELK | hsa-miR-520c-3p | FGD5-AS1 |
| MELK | hsa-miR-520d-3p | FGD5-AS1 |
| MELK | hsa-miR-520e | FGD5-AS1 |
| MELK | hsa-miR-526b-3p | FGD5-AS1 |
| MELK | hsa-miR-93-5p | FGD5-AS1 |
| MELK | hsa-miR-106a-5p | MANCR |
| MELK | hsa-miR-106b-5p | MANCR |
| MELK | hsa-miR-17-5p | MANCR |
| MELK | hsa-miR-20a-5p | MANCR |
| MELK | hsa-miR-20b-5p | MANCR |
| MELK | hsa-miR-302a-3p | MANCR |
| MELK | hsa-miR-302b-3p | MANCR |
| MELK | hsa-miR-302c-3p | MANCR |
| MELK | hsa-miR-302d-3p | MANCR |
| MELK | hsa-miR-302e | MANCR |
| MELK | hsa-miR-372-3p | MANCR |
| MELK | hsa-miR-373-3p | MANCR |
| MELK | hsa-miR-519d-3p | MANCR |
| MELK | hsa-miR-520a-3p | MANCR |
| MELK | hsa-miR-520b | MANCR |
| MELK | hsa-miR-520c-3p | MANCR |
| MELK | hsa-miR-520d-3p | MANCR |
| MELK | hsa-miR-520e | MANCR |
| MELK | hsa-miR-520g-3p | MANCR |
| MELK | hsa-miR-520h | MANCR |
| MELK | hsa-miR-526b-3p | MANCR |
| MELK | hsa-miR-93-5p | MANCR |
| MELK | hsa-miR-106a-5p | PCAT19 |
| MELK | hsa-miR-106b-5p | PCAT19 |
| MELK | hsa-miR-17-5p | PCAT19 |
| MELK | hsa-miR-20a-5p | PCAT19 |
| MELK | hsa-miR-20b-5p | PCAT19 |
| MELK | hsa-miR-302a-3p | PCAT19 |
| MELK | hsa-miR-302b-3p | PCAT19 |
| MELK | hsa-miR-302c-3p | PCAT19 |
| MELK | hsa-miR-302d-3p | PCAT19 |
| MELK | hsa-miR-302e | PCAT19 |
| MELK | hsa-miR-372-3p | PCAT19 |
| MELK | hsa-miR-373-3p | PCAT19 |
| MELK | hsa-miR-519d-3p | PCAT19 |
| MELK | hsa-miR-520a-3p | PCAT19 |
| MELK | hsa-miR-520b | PCAT19 |
| MELK | hsa-miR-520c-3p | PCAT19 |
| MELK | hsa-miR-520d-3p | PCAT19 |
| MELK | hsa-miR-520e | PCAT19 |
| MELK | hsa-miR-520g-3p | PCAT19 |
| MELK | hsa-miR-520h | PCAT19 |
| MELK | hsa-miR-526b-3p | PCAT19 |
| MELK | hsa-miR-93-5p | PCAT19 |
| MELK | hsa-miR-106a-5p | SNHG16 |
| MELK | hsa-miR-106b-5p | SNHG16 |
| MELK | hsa-miR-17-5p | SNHG16 |
| MELK | hsa-miR-20a-5p | SNHG16 |
| MELK | hsa-miR-20b-5p | SNHG16 |
| MELK | hsa-miR-302a-3p | SNHG16 |
| MELK | hsa-miR-302b-3p | SNHG16 |
| MELK | hsa-miR-302c-3p | SNHG16 |
| MELK | hsa-miR-302d-3p | SNHG16 |
| MELK | hsa-miR-302e | SNHG16 |
| MELK | hsa-miR-372-3p | SNHG16 |
| MELK | hsa-miR-373-3p | SNHG16 |
| MELK | hsa-miR-519d-3p | SNHG16 |
| MELK | hsa-miR-520a-3p | SNHG16 |
| MELK | hsa-miR-520b | SNHG16 |
| MELK | hsa-miR-520c-3p | SNHG16 |
| MELK | hsa-miR-520d-3p | SNHG16 |
| MELK | hsa-miR-520e | SNHG16 |
| MELK | hsa-miR-526b-3p | SNHG16 |
| MELK | hsa-miR-93-5p | SNHG16 |
| MELK | hsa-miR-106a-5p | ZFHX2-AS1 |
| MELK | hsa-miR-106b-5p | ZFHX2-AS1 |
| MELK | hsa-miR-17-5p | ZFHX2-AS1 |
| MELK | hsa-miR-20a-5p | ZFHX2-AS1 |
| MELK | hsa-miR-20b-5p | ZFHX2-AS1 |
| MELK | hsa-miR-302a-3p | ZFHX2-AS1 |
| MELK | hsa-miR-302b-3p | ZFHX2-AS1 |
| MELK | hsa-miR-302c-3p | ZFHX2-AS1 |
| MELK | hsa-miR-302d-3p | ZFHX2-AS1 |
| MELK | hsa-miR-302e | ZFHX2-AS1 |
| MELK | hsa-miR-372-3p | ZFHX2-AS1 |
| MELK | hsa-miR-373-3p | ZFHX2-AS1 |
| MELK | hsa-miR-519d-3p | ZFHX2-AS1 |
| MELK | hsa-miR-520a-3p | ZFHX2-AS1 |
| MELK | hsa-miR-520b | ZFHX2-AS1 |
| MELK | hsa-miR-520c-3p | ZFHX2-AS1 |
| MELK | hsa-miR-520d-3p | ZFHX2-AS1 |
| MELK | hsa-miR-520e | ZFHX2-AS1 |
| MELK | hsa-miR-520g-3p | ZFHX2-AS1 |
| MELK | hsa-miR-520h | ZFHX2-AS1 |
| MELK | hsa-miR-526b-3p | ZFHX2-AS1 |
| MELK | hsa-miR-93-5p | ZFHX2-AS1 |
| MELK | hsa-miR-106a-5p | ERC2-IT1 |
| MELK | hsa-miR-106b-5p | ERC2-IT1 |
| MELK | hsa-miR-17-5p | ERC2-IT1 |
| MELK | hsa-miR-20a-5p | ERC2-IT1 |
| MELK | hsa-miR-20b-5p | ERC2-IT1 |
| MELK | hsa-miR-302a-3p | ERC2-IT1 |
| MELK | hsa-miR-302b-3p | ERC2-IT1 |
| MELK | hsa-miR-302c-3p | ERC2-IT1 |
| MELK | hsa-miR-302d-3p | ERC2-IT1 |
| MELK | hsa-miR-302e | ERC2-IT1 |
| MELK | hsa-miR-372-3p | ERC2-IT1 |
| MELK | hsa-miR-373-3p | ERC2-IT1 |
| MELK | hsa-miR-519d-3p | ERC2-IT1 |
| MELK | hsa-miR-520a-3p | ERC2-IT1 |
| MELK | hsa-miR-520b | ERC2-IT1 |
| MELK | hsa-miR-520c-3p | ERC2-IT1 |
| MELK | hsa-miR-520d-3p | ERC2-IT1 |
| MELK | hsa-miR-520e | ERC2-IT1 |
| MELK | hsa-miR-526b-3p | ERC2-IT1 |
| MELK | hsa-miR-93-5p | ERC2-IT1 |
| MELK | hsa-miR-106a-5p | SNHG20 |
| MELK | hsa-miR-106b-5p | SNHG20 |
| MELK | hsa-miR-17-5p | SNHG20 |
| MELK | hsa-miR-20a-5p | SNHG20 |
| MELK | hsa-miR-20b-5p | SNHG20 |
| MELK | hsa-miR-302a-3p | SNHG20 |
| MELK | hsa-miR-302b-3p | SNHG20 |
| MELK | hsa-miR-302c-3p | SNHG20 |
| MELK | hsa-miR-302d-3p | SNHG20 |
| MELK | hsa-miR-302e | SNHG20 |
| MELK | hsa-miR-372-3p | SNHG20 |
| MELK | hsa-miR-373-3p | SNHG20 |
| MELK | hsa-miR-519d-3p | SNHG20 |
| MELK | hsa-miR-520a-3p | SNHG20 |
| MELK | hsa-miR-520b | SNHG20 |
| MELK | hsa-miR-520c-3p | SNHG20 |
| MELK | hsa-miR-520d-3p | SNHG20 |
| MELK | hsa-miR-520e | SNHG20 |
| MELK | hsa-miR-526b-3p | SNHG20 |
| MELK | hsa-miR-93-5p | SNHG20 |
| MELK | hsa-miR-106a-5p | LINC00632 |
| MELK | hsa-miR-17-5p | LINC00632 |
| MELK | hsa-miR-20a-5p | LINC00632 |
| MELK | hsa-miR-20b-5p | LINC00632 |
| MELK | hsa-miR-302a-3p | LINC00632 |
| MELK | hsa-miR-302b-3p | LINC00632 |
| MELK | hsa-miR-302c-3p | LINC00632 |
| MELK | hsa-miR-302d-3p | LINC00632 |
| MELK | hsa-miR-372-3p | LINC00632 |
| MELK | hsa-miR-373-3p | LINC00632 |
| MELK | hsa-miR-519d-3p | LINC00632 |
| MELK | hsa-miR-520a-3p | LINC00632 |
| MELK | hsa-miR-520b | LINC00632 |
| MELK | hsa-miR-520c-3p | LINC00632 |
| MELK | hsa-miR-520d-3p | LINC00632 |
| MELK | hsa-miR-520e | LINC00632 |
| MELK | hsa-miR-520g-3p | LINC00632 |
| MELK | hsa-miR-526b-3p | LINC00632 |
| MELK | hsa-miR-93-5p | LINC00632 |
| MELK | hsa-miR-302a-3p | MIR34AHG |
| MELK | hsa-miR-302b-3p | MIR34AHG |
| MELK | hsa-miR-302c-3p | MIR34AHG |
| MELK | hsa-miR-302d-3p | MIR34AHG |
| MELK | hsa-miR-302e | MIR34AHG |
| MELK | hsa-miR-372-3p | MIR34AHG |
| MELK | hsa-miR-373-3p | MIR34AHG |
| MELK | hsa-miR-520a-3p | MIR34AHG |
| MELK | hsa-miR-520b | MIR34AHG |
| MELK | hsa-miR-520c-3p | MIR34AHG |
| MELK | hsa-miR-520d-3p | MIR34AHG |
| MELK | hsa-miR-520e | MIR34AHG |
| MELK | hsa-miR-1307-3p | LINC00472 |
| MELK | hsa-miR-302a-3p | LINC00472 |
| MELK | hsa-miR-302b-3p | LINC00472 |
| MELK | hsa-miR-302c-3p | LINC00472 |
| MELK | hsa-miR-302d-3p | LINC00472 |
| MELK | hsa-miR-302e | LINC00472 |
| MELK | hsa-miR-372-3p | LINC00472 |
| MELK | hsa-miR-373-3p | LINC00472 |
| MELK | hsa-miR-520a-3p | LINC00472 |
| MELK | hsa-miR-520b | LINC00472 |
| MELK | hsa-miR-520c-3p | LINC00472 |
| MELK | hsa-miR-520d-3p | LINC00472 |
| MELK | hsa-miR-520e | LINC00472 |
| MELK | hsa-miR-302a-3p | LINC01106 |
| MELK | hsa-miR-302b-3p | LINC01106 |
| MELK | hsa-miR-302c-3p | LINC01106 |
| MELK | hsa-miR-302d-3p | LINC01106 |
| MELK | hsa-miR-302e | LINC01106 |
| MELK | hsa-miR-372-3p | LINC01106 |
| MELK | hsa-miR-373-3p | LINC01106 |
| MELK | hsa-miR-520a-3p | LINC01106 |
| MELK | hsa-miR-520b | LINC01106 |
| MELK | hsa-miR-520c-3p | LINC01106 |
| MELK | hsa-miR-520d-3p | LINC01106 |
| MELK | hsa-miR-520e | LINC01106 |
| MELK | hsa-miR-302a-3p | MIR17HG |
| MELK | hsa-miR-302b-3p | MIR17HG |
| MELK | hsa-miR-302c-3p | MIR17HG |
| MELK | hsa-miR-302d-3p | MIR17HG |
| MELK | hsa-miR-302e | MIR17HG |
| MELK | hsa-miR-372-3p | MIR17HG |
| MELK | hsa-miR-373-3p | MIR17HG |
| MELK | hsa-miR-520a-3p | MIR17HG |
| MELK | hsa-miR-520b | MIR17HG |
| MELK | hsa-miR-520c-3p | MIR17HG |
| MELK | hsa-miR-520d-3p | MIR17HG |
| MELK | hsa-miR-520e | MIR17HG |
| MELK | hsa-miR-302a-3p | NNT-AS1 |
| MELK | hsa-miR-302b-3p | NNT-AS1 |
| MELK | hsa-miR-302c-3p | NNT-AS1 |
| MELK | hsa-miR-302d-3p | NNT-AS1 |
| MELK | hsa-miR-302e | NNT-AS1 |
| MELK | hsa-miR-372-3p | NNT-AS1 |
| MELK | hsa-miR-373-3p | NNT-AS1 |
| MELK | hsa-miR-520a-3p | NNT-AS1 |
| MELK | hsa-miR-520b | NNT-AS1 |
| MELK | hsa-miR-520c-3p | NNT-AS1 |
| MELK | hsa-miR-520d-3p | NNT-AS1 |
| MELK | hsa-miR-520e | NNT-AS1 |
| MELK | hsa-miR-302a-3p | TAPT1-AS1 |
| MELK | hsa-miR-302b-3p | TAPT1-AS1 |
| MELK | hsa-miR-302c-3p | TAPT1-AS1 |
| MELK | hsa-miR-302d-3p | TAPT1-AS1 |
| MELK | hsa-miR-302e | TAPT1-AS1 |
| MELK | hsa-miR-372-3p | TAPT1-AS1 |
| MELK | hsa-miR-373-3p | TAPT1-AS1 |
| MELK | hsa-miR-520a-3p | TAPT1-AS1 |
| MELK | hsa-miR-520b | TAPT1-AS1 |
| MELK | hsa-miR-520c-3p | TAPT1-AS1 |
| MELK | hsa-miR-520d-3p | TAPT1-AS1 |
| MELK | hsa-miR-520e | TAPT1-AS1 |
| MELK | hsa-miR-302a-3p | ZNF667-AS1 |
| MELK | hsa-miR-302b-3p | ZNF667-AS1 |
| MELK | hsa-miR-302c-3p | ZNF667-AS1 |
| MELK | hsa-miR-302d-3p | ZNF667-AS1 |
| MELK | hsa-miR-302e | ZNF667-AS1 |
| MELK | hsa-miR-372-3p | ZNF667-AS1 |
| MELK | hsa-miR-373-3p | ZNF667-AS1 |
| MELK | hsa-miR-520a-3p | ZNF667-AS1 |
| MELK | hsa-miR-520b | ZNF667-AS1 |
| MELK | hsa-miR-520c-3p | ZNF667-AS1 |
| MELK | hsa-miR-520d-3p | ZNF667-AS1 |
| MELK | hsa-miR-520e | ZNF667-AS1 |
| MELK | hsa-miR-106a-5p | EPB41L4A-AS1 |
| MELK | hsa-miR-106b-5p | EPB41L4A-AS1 |
| MELK | hsa-miR-17-5p | EPB41L4A-AS1 |
| MELK | hsa-miR-20a-5p | EPB41L4A-AS1 |
| MELK | hsa-miR-20b-5p | EPB41L4A-AS1 |
| MELK | hsa-miR-519d-3p | EPB41L4A-AS1 |
| MELK | hsa-miR-520g-3p | EPB41L4A-AS1 |
| MELK | hsa-miR-520h | EPB41L4A-AS1 |
| MELK | hsa-miR-526b-3p | EPB41L4A-AS1 |
| MELK | hsa-miR-93-5p | EPB41L4A-AS1 |
| MELK | hsa-miR-302a-3p | LINC01748 |
| MELK | hsa-miR-302b-3p | LINC01748 |
| MELK | hsa-miR-302c-3p | LINC01748 |
| MELK | hsa-miR-302d-3p | LINC01748 |
| MELK | hsa-miR-372-3p | LINC01748 |
| MELK | hsa-miR-373-3p | LINC01748 |
| MELK | hsa-miR-520a-3p | LINC01748 |
| MELK | hsa-miR-520b | LINC01748 |
| MELK | hsa-miR-520c-3p | LINC01748 |
| MELK | hsa-miR-520d-3p | LINC01748 |
| MELK | hsa-miR-520e | LINC01748 |
| MELK | hsa-miR-106a-5p | ATP6V1B1-AS1 |
| MELK | hsa-miR-106b-5p | ATP6V1B1-AS1 |
| MELK | hsa-miR-17-5p | ATP6V1B1-AS1 |
| MELK | hsa-miR-20a-5p | ATP6V1B1-AS1 |
| MELK | hsa-miR-20b-5p | ATP6V1B1-AS1 |
| MELK | hsa-miR-519d-3p | ATP6V1B1-AS1 |
| MELK | hsa-miR-520g-3p | ATP6V1B1-AS1 |
| MELK | hsa-miR-520h | ATP6V1B1-AS1 |
| MELK | hsa-miR-526b-3p | ATP6V1B1-AS1 |
| MELK | hsa-miR-93-5p | ATP6V1B1-AS1 |
| MELK | hsa-miR-106a-5p | HOTAIR |
| MELK | hsa-miR-106b-5p | HOTAIR |
| MELK | hsa-miR-17-5p | HOTAIR |
| MELK | hsa-miR-20a-5p | HOTAIR |
| MELK | hsa-miR-20b-5p | HOTAIR |
| MELK | hsa-miR-519d-3p | HOTAIR |
| MELK | hsa-miR-520g-3p | HOTAIR |
| MELK | hsa-miR-520h | HOTAIR |
| MELK | hsa-miR-526b-3p | HOTAIR |
| MELK | hsa-miR-93-5p | HOTAIR |
| MELK | hsa-miR-106a-5p | LINC00839 |
| MELK | hsa-miR-106b-5p | LINC00839 |
| MELK | hsa-miR-17-5p | LINC00839 |
| MELK | hsa-miR-20a-5p | LINC00839 |
| MELK | hsa-miR-20b-5p | LINC00839 |
| MELK | hsa-miR-519d-3p | LINC00839 |
| MELK | hsa-miR-520g-3p | LINC00839 |
| MELK | hsa-miR-520h | LINC00839 |
| MELK | hsa-miR-526b-3p | LINC00839 |
| MELK | hsa-miR-93-5p | LINC00839 |
| MELK | hsa-miR-106a-5p | MALAT1 |
| MELK | hsa-miR-106b-5p | MALAT1 |
| MELK | hsa-miR-17-5p | MALAT1 |
| MELK | hsa-miR-20a-5p | MALAT1 |
| MELK | hsa-miR-20b-5p | MALAT1 |
| MELK | hsa-miR-519d-3p | MALAT1 |
| MELK | hsa-miR-526b-3p | MALAT1 |
| MELK | hsa-miR-93-5p | MALAT1 |
| MELK | hsa-miR-106a-5p | H19 |
| MELK | hsa-miR-106b-5p | H19 |
| MELK | hsa-miR-17-5p | H19 |
| MELK | hsa-miR-20a-5p | H19 |
| MELK | hsa-miR-20b-5p | H19 |
| MELK | hsa-miR-519d-3p | H19 |
| MELK | hsa-miR-526b-3p | H19 |
| MELK | hsa-miR-93-5p | H19 |
| MELK | hsa-miR-106a-5p | CKMT2-AS1 |
| MELK | hsa-miR-106b-5p | CKMT2-AS1 |
| MELK | hsa-miR-17-5p | CKMT2-AS1 |
| MELK | hsa-miR-20a-5p | CKMT2-AS1 |
| MELK | hsa-miR-20b-5p | CKMT2-AS1 |
| MELK | hsa-miR-519d-3p | CKMT2-AS1 |
| MELK | hsa-miR-526b-3p | CKMT2-AS1 |
| MELK | hsa-miR-93-5p | CKMT2-AS1 |
| MELK | hsa-miR-106a-5p | ERICD |
| MELK | hsa-miR-106b-5p | ERICD |
| MELK | hsa-miR-17-5p | ERICD |
| MELK | hsa-miR-20a-5p | ERICD |
| MELK | hsa-miR-20b-5p | ERICD |
| MELK | hsa-miR-519d-3p | ERICD |
| MELK | hsa-miR-526b-3p | ERICD |
| MELK | hsa-miR-93-5p | ERICD |
| MELK | hsa-miR-106a-5p | LINC01003 |
| MELK | hsa-miR-106b-5p | LINC01003 |
| MELK | hsa-miR-17-5p | LINC01003 |
| MELK | hsa-miR-20a-5p | LINC01003 |
| MELK | hsa-miR-20b-5p | LINC01003 |
| MELK | hsa-miR-519d-3p | LINC01003 |
| MELK | hsa-miR-526b-3p | LINC01003 |
| MELK | hsa-miR-93-5p | LINC01003 |
| MELK | hsa-miR-106a-5p | LINC01618 |
| MELK | hsa-miR-106b-5p | LINC01618 |
| MELK | hsa-miR-17-5p | LINC01618 |
| MELK | hsa-miR-20a-5p | LINC01618 |
| MELK | hsa-miR-20b-5p | LINC01618 |
| MELK | hsa-miR-519d-3p | LINC01618 |
| MELK | hsa-miR-526b-3p | LINC01618 |
| MELK | hsa-miR-93-5p | LINC01618 |
| MELK | hsa-miR-193b-3p | LINC01089 |
| MELK | hsa-miR-302a-3p | LINC01694 |
| MELK | hsa-miR-302b-3p | LINC01694 |
| MELK | hsa-miR-302c-3p | LINC01694 |
| MELK | hsa-miR-302d-3p | LINC01694 |
| MELK | hsa-miR-372-3p | LINC01694 |
| MELK | hsa-miR-373-3p | LINC01694 |
| MELK | hsa-miR-193b-3p | MIR4435-2HG |
| MELK | hsa-miR-371a-5p | MIR4435-2HG |
| MELK | hsa-miR-520g-3p | MIR4435-2HG |
| MELK | hsa-miR-520h | MIR4435-2HG |
| MELK | hsa-miR-302c-3p | CARD8-AS1 |
| MELK | hsa-miR-302d-3p | CARD8-AS1 |
| MELK | hsa-miR-372-3p | CARD8-AS1 |
| MELK | hsa-miR-373-3p | CARD8-AS1 |
| MELK | hsa-miR-520a-3p | CARD8-AS1 |
| MELK | hsa-miR-193b-3p | SNHG7 |
| MELK | hsa-miR-520g-3p | SNHG7 |
| MELK | hsa-miR-520h | SNHG7 |
| MELK | hsa-miR-193b-3p | MIR194-2HG |
| MELK | hsa-miR-193b-3p | MIRLET7BHG |
| MELK | hsa-miR-193b-3p | LINC01184 |
| MELK | hsa-miR-371a-5p | PART1 |
| MELK | hsa-miR-4712-5p | PART1 |
| MELK | hsa-miR-770-5p | PART1 |
| MELK | hsa-miR-520g-3p | ADAMTSL4-AS1 |
| MELK | hsa-miR-520h | ADAMTSL4-AS1 |
| MELK | hsa-miR-4712-5p | AFDN-DT |
| MELK | hsa-miR-770-5p | AFDN-DT |
| MELK | hsa-miR-520g-3p | BACH1-IT2 |
| MELK | hsa-miR-520h | BACH1-IT2 |
| MELK | hsa-miR-193b-3p | CYTOR |
| MELK | hsa-miR-520g-3p | HCG11 |
| MELK | hsa-miR-520h | HCG11 |
| MELK | hsa-miR-371a-5p | MIR29B2CHG |
| MELK | hsa-miR-1307-3p | MIR600HG |
| MELK | hsa-miR-4712-5p | MIR9-3HG |
| MELK | hsa-miR-770-5p | MIR9-3HG |
| MELK | hsa-miR-4712-5p | RPARP-AS1 |
| MELK | hsa-miR-770-5p | RPARP-AS1 |
| MELK | hsa-miR-1307-3p | SNHG25 |
| MELK | hsa-miR-371a-5p | SNHG3 |
| MELK | hsa-miR-520g-3p | THAP9-AS1 |
| MELK | hsa-miR-520h | THAP9-AS1 |
| MELK | hsa-miR-193b-3p | ZFAS1 |
| MELK | hsa-miR-371a-5p | CCDC18-AS1 |
| MELK | hsa-miR-371a-5p | ILF3-DT |
| MELK | hsa-miR-371a-5p | KCNIP4-IT1 |
| MELK | hsa-miR-371a-5p | PRNCR1 |
| MELK | hsa-miR-371a-5p | SNHG15 |

**Supplementary Table 12.** Integrated regulatory network of hub genes, miRNAs, and TFs

| miRNA/TF | BUB1B | KIF14 | MELK |
| --- | --- | --- | --- |
| hsa-miR-106a-5p |  |  | 1 |
| hsa-miR-106b-5p |  |  | 1 |
| hsa-miR-1277-5p |  | 1 |  |
| hsa-miR-1307-3p |  |  | 1 |
| hsa-miR-155-5p |  | 1 |  |
| hsa-miR-16-5p |  | 1 |  |
| hsa-miR-17-5p |  |  | 1 |
| hsa-miR-186-5p |  | 1 |  |
| hsa-miR-192-5p | 1 | 1 |  |
| hsa-miR-193b-3p | 1 |  | 1 |
| hsa-miR-20a-5p |  |  | 1 |
| hsa-miR-20b-5p |  |  | 1 |
| hsa-miR-215-5p | 1 | 1 |  |
| hsa-miR-22-3p | 1 |  |  |
| hsa-miR-302a-3p |  |  | 1 |
| hsa-miR-302b-3p |  |  | 1 |
| hsa-miR-302c-3p |  |  | 1 |
| hsa-miR-302d-3p |  |  | 1 |
| hsa-miR-302e |  |  | 1 |
| hsa-miR-371a-5p |  |  | 1 |
| hsa-miR-372-3p |  |  | 1 |
| hsa-miR-373-3p |  |  | 1 |
| hsa-miR-4712-5p |  |  | 1 |
| hsa-miR-519d-3p |  |  | 1 |
| hsa-miR-520a-3p |  |  | 1 |
| hsa-miR-520b |  |  | 1 |
| hsa-miR-520c-3p |  |  | 1 |
| hsa-miR-520d-3p |  |  | 1 |
| hsa-miR-520e |  |  | 1 |
| hsa-miR-520g-3p |  |  | 1 |
| hsa-miR-520h |  |  | 1 |
| hsa-miR-526b-3p |  |  | 1 |
| hsa-miR-615-3p |  | 1 |  |
| hsa-miR-770-5p |  |  | 1 |
| hsa-miR-93-5p |  |  | 1 |
| JUN | 1 |  |  |
| AF4 | 1 | 1 | 1 |
| AP1S2 | 1 |  |  |
| AR |  |  | 1 |
| ASH2L |  |  | 1 |
| BCL6 |  | 1 |  |
| BCOR |  | 1 |  |
| BRD4 | 1 |  | 1 |
| CHD1 |  | 1 |  |
| CREB1 | 1 |  |  |
| CREM |  | 1 |  |
| CTCF | 1 | 1 |  |
| DACH1 |  |  | 1 |
| E2F1 | 1 | 1 | 1 |
| E2F4 | 1 | 1 |  |
| ENL |  |  | 1 |
| ERG |  |  | 1 |
| ESRRB | 1 |  |  |
| ETS1 | 1 |  |  |
| ETV1 |  | 1 |  |
| FLI1 |  | 1 | 1 |
| FOSL1 |  | 1 |  |
| FOXA1 | 1 | 1 | 1 |
| FOXA2 | 1 |  |  |
| FOXM1 | 1 | 1 |  |
| FOXO3 |  | 1 |  |
| FOXP1 |  | 1 |  |
| FOXP2 | 1 |  |  |
| FOXP3 |  | 1 |  |
| GATA2 |  | 1 | 1 |
| GATA3 |  | 1 |  |
| GATA6 |  | 1 |  |
| HNF4A | 1 |  |  |
| HOXB13 |  | 1 |  |
| HOXC9 | 1 |  |  |
| JUND | 1 |  |  |
| KDM5B | 1 | 1 | 1 |
| KDM6A | 1 | 1 |  |
| KLF1 |  |  | 1 |
| KLF4 |  | 1 |  |
| KLF6 |  | 1 |  |
| MAF |  | 1 |  |
| MYB |  | 1 |  |
| MYBL2 | 1 |  |  |
| MYC | 1 |  | 1 |
| NACC1 | 1 |  |  |
| NANOG | 1 | 1 |  |
| NCOR1 | 1 |  |  |
| NFKB1 |  |  | 1 |
| NFYA |  | 1 |  |
| NFYB |  | 1 |  |
| NOTCH1 | 1 |  |  |
| NR1H3 |  |  | 1 |
| NR1I2 |  | 1 |  |
| NR3C1 |  |  | 1 |
| NRF2 |  | 1 |  |
| P300 |  | 1 |  |
| PCGF4 |  | 1 |  |
| PHF8 |  | 1 | 1 |
| PHOX2B |  | 1 |  |
| POU5F1 | 1 | 1 |  |
| PPARD |  |  | 1 |
| PPARG | 1 |  |  |
| PRDM14 | 1 |  |  |
| PRDM5 |  |  | 1 |
| RAC3 |  | 1 |  |
| RBPJ |  | 1 |  |
| RCOR3 |  | 1 | 1 |
| REST |  |  | 1 |
| RUNX1 | 1 |  |  |
| RUNX2 |  | 1 |  |
| SETDB1 |  | 1 |  |
| SIN3B |  | 1 | 1 |
| SMAD3 | 1 |  |  |
| SMAD4 |  | 1 |  |
| SMRT |  |  | 1 |
| SOX17 | 1 |  |  |
| SOX2 | 1 | 1 |  |
| SPI1 | 1 | 1 |  |
| SREBF1 | 1 |  |  |
| SRF | 1 |  |  |
| STAT1 |  |  | 1 |
| STAT3 |  | 1 |  |
| TBL1 |  | 1 |  |
| TBX2 |  |  | 1 |
| TEAD4 |  | 1 |  |
| TET1 | 1 |  |  |
| TP63 | 1 |  |  |
| UTX |  | 1 |  |
| WT1 | 1 |  |  |
| YAP1 |  | 1 | 1 |
| YY1 | 1 | 1 |  |
| ZBTB16 | 1 |  |  |
| ZNF281 | 1 |  |  |
| Total connections | 45 | 58 | 54 |

Note: “1” indicates a connection exists.
